# ILC2 regulate a fibroblast progenitor niche in the pancreas

**DOI:** 10.1126/science.aea5113

**Published:** 2026-07-02

**Authors:** Thomas Yip, Julie Stockis, Charlotte Simpson, Erika E. McCartney, Shwetha Raghunathan, Martha M. Rangel-Sosa, Sydney N. Hummel, Julia Moreno-Vicente, Gianmarco Raddi, Celine Garcia, Rugile Linkute, Silvain Pinaud, Maye F. Cheng, Lesley A. Hill, T. Michael Underhill, Hans-Reimer Rodewald, Christoph Schneider, Claus Jørgensen, Andrew N.J. McKenzie, Sophie E. Acton, Patrick Seale, Menna R. Clatworthy, Matthew B. Buechler, Timotheus Y.F. Halim

**Affiliations:** 1 University of Cambridge, CRUK Cambridge Institute, Cambridge, UK; 2 University of Toronto, Department of Immunology, Toronto, Canada; 3 Department of Cellular and Physiological Sciences and Biomedical Research Centre, University of British Columbia, Vancouver, BC, Canada; 4 Division of Cellular Immunology, German Cancer Research Center; Heidelberg, Germany; 5 Department of Physiology, University of Zurich, Zurich, Switzerland; 6 University of Manchester, Cancer Research UK Manchester Institute, Manchester, UK; 7 MRC laboratory of Molecular Biology, Cambridge, UK; 8 Laboratory for Molecular Cell Biology, University College London, London, UK; 9 Department of Cell and Developmental Biology, Perelman School of Medicine at the University of Pennsylvania, Philadelphia, USA; 10 Institute for Diabetes, Obesity and Metabolism, Perelman School of Medicine at the University of Pennsylvania, Philadelphia, USA; 11 Cambridge Institute for Therapeutic Immunology and Infectious Diseases, University of Cambridge, Department of Medicine, Cambridge, UK; 12 Schroeder Arthritis Institute, University Health Network, Toronto, Ontario Canada

## Abstract

Local fibroblast development and densities influence organ health and disease, although it remains unclear how tissue fibroblast topography is controlled *in situ*. Here we defined group 2 innate lymphoid cells (ILC2) as key regulators of fibroblast homeostasis in the pancreas. ILC2 co-localised with fibroblasts expressing the genes *Pi16*^*+*^*Dpp4*^*+*^*Ly6c*^*+*^ in an interstitial niche of the exocrine pancreas, which encapsulates the organ parenchyma. ILC2 specifically regulated the expansion of *Pi16*^*+*^*Dpp4*^*+*^*Ly6c*^*+*^ fibroblasts, which have progenitor capacity, while restraining differentiated intra-parenchymal *Col15a1*^*+*^ fibroblasts during inflammation. These circuits reinforced fibroblast numbers after injury and set an inflammatory threshold. The ILC2 and *Pi16*^*+*^*Dpp4*^*+*^*Ly6c*^*+*^ fibroblast progenitor niche expanded around tumours and controlled cancer associated fibroblast ontogeny and density. Hence, ILC2-fibroblast dialogue represents a regulatory node that locally orchestrates tissue homeostasis and pathology.

## Introduction

Fibroblasts represent a heterogeneous population of tissue-resident cells found in almost all organs (1) with roles in tissue structure, organ homeostasis, inflammation and tissue repair (2). Conversely, their dysregulation drives fibrotic diseases and tumourigenesis (2, 3). The stromal niche is attuned to both specific organ sites, and to diverse physiological or pathological cues. An increased appreciation of stromal cell diversity under both homeostatic and pathological conditions has renewed questions about fibroblast ontogeny (4). A fibroblast subset marked by expression of the genes *Pi16, Dpp4* and *Ly6c1* is present in multiple organs at rest or in disease states (1); yet how this population is locally maintained, and its biological or pathological functions remains unknown.

Tissue-resident lymphocytes, including Group 2 innate lymphoid cells (ILC2), are important mediators of tissue homeostasis (5–7). ILC2 inhabit epithelial stem cell niches at mucosal barrier sites, where they directly influence epithelial cell biology (8, 9). Moreover, ILC2-derived interleukin (IL)-13 promotes fibroblast activation and fibrosis; in addition, ILC2 are directly activated by epithelial or fibroblast-derived cytokines, including the damage-associated alarmin IL-33 (10–14). Hence, local fibroblast-ILC2 interactions are proposed to drive a feed-forward loop that underpins tissue fibrosis. However, much remains unknown about the function of ILC2 during steady-state, resolution of inflammation, and cancer.

The exocrine pancreas produces digestive enzymes and is comprised of acinar and ductal epithelial cells that make up the bulk of this organ. Fibroblast subsets are locally maintained (1) and participate in injury-driven inflammation (15). Stressed or necrotic fibroblasts release IL-33 (16) which implies the concomitant liberation of other damage-associated molecular patterns (DAMPs). Nevertheless, how local fibroblast composition dictates the acute injury response remains uncertain. Moreover, molecularly and functionally diverse cancer associated fibroblasts (CAF) subtypes play crucial roles in pancreatic ductal adenocarcinoma (PDAC), however, many questions remain about their ontogeny (17, 18).

## Results

### ILC2 co-localised with IL-33^+^ fibroblasts in an interstitial niche of the pancreas

**Fig. 1:**
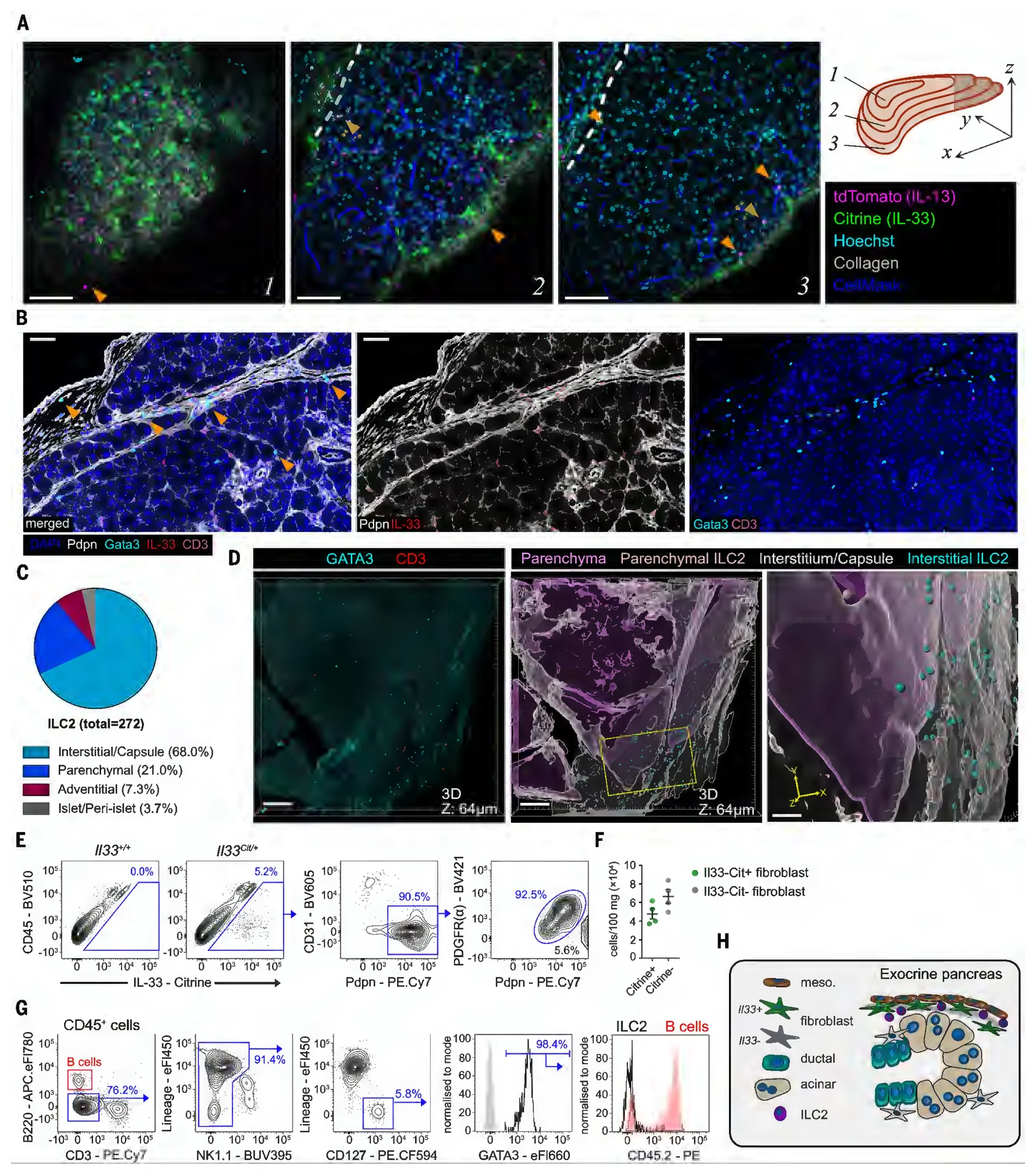
ILC2 co-localise with IL-33^+^ fibroblast in an interstitial niche of the pancreas. (**A**) Pancreas explants from *Il13*^*tdTom/+*^*Il33*^*Cit/+*^ mice pre-treated with IL-33 on days -3 and -2 were imaged by 2-photon microscopy immediately after dissection at different depths (see z-stack orientation, right). Dotted line indicates interstitial septae, arrows highlight representative tdTom^+^ cells. CellMask was used to stain cell membranes of vasculature and parenchymal acinar cells. Scale bar: 100 μm. (**B-C**) Formalin fixed paraffin embedded (FFPE) pancreata from C57Bl/6 mice pre-treated as in (A) were sectioned and analysed using multiplex immunofluorescence (IF) imaging. A representative region is shown in (**B**), indicating localisation of Gata3^+^CD3^-^ ILC2 (arrow highlights representative ILC2), Podoplanin (Pdpn) and IL-33 staining. HALO image analysis on tissue sections determined the proportion of ILC2 in the indicated anatomical regions (**C**). Scale bar: 50 μm. (**D**) Maximum intensity projection of 3D images of an optically clear thick section from an untreated C57Bl/6 mouse pancreas stained for GATA3 and CD3 (left). GATA3^+^CD3^-^ cells were reconstructed as ILC2s and stratified based on their location as parenchymal (light pink) and interstitial (cyan); parenchyma (purple) and interstitium (white) were reconstructed based on tissue autofluorescence (middle). The indicated ROI (yellow) was magnified and rotated to provide a clearer view of interstitial ILC2s (right). Scale bar: 100 μm (*left* *and* *middle*), 30 μm (*right*). (**E-F**) Pancreata from untreated *Il33*^*Cit/+*^ and C57Bl/6 (*Il33*^*+/+*^) control mice were analysed by flow cytometry to determine the phenotype of IL-33^Cit+^ cells (**E**); the absolute number of fibroblasts was calculated (**F**). Datapoints represent individual mice. (**G**) Untreated C57Bl/6 mice were injected with anti-CD45.2-PE antibody before sacrifice to label circulating cells; pancreata were analysed by flow cytometry for CD45.2 expression on the indicated immune cell-types *(related to Fig. S1I)*. (**H**) Diagram of pancreatic ILC2-fibroblast niche. Dot plots show representative gating strategy; numbers indicate percent of gated cells. Additional gating strategy for (**F**) shown in Fig. S1F. Imaging data is representative of 2 (**A**), 3 (**B**), and 2 (**D**) individual mice. Bar graphs indicate mean (±SEM) and show data representative of two independent experiment (n=4 per group, **F**).

We used 2-photon microscopy of *Il13*^*tdTom/+*^*Il33*^*Cit/+*^ mouse pancreata to visualise the anatomical location of ILC2 in relation to IL-33-producing cells (Fig. 1A, S1A). Mice were pre-treated with IL-33 to enhance fluorescent Il13-tdTomato signal, required for ILC2 visualisation. While ILC2 are reported in the islets of Langerhans and surrounding large blood vessels (13, 19), our data indicated that IL-13^tdTom+^ cells were enriched in the interstitial septae and capsule that surround the organ parenchyma. Moreover, we found that IL-33^Cit+^ cells inhabited the same collagen-rich niche and co-localised with IL-13^tdTom+^ cells (Fig. S1B). Given the limitations in imaging depth of 2-photon microscopy we also performed multiplex immunofluorescence (IF) imaging of larger tissue sections. We identified ILC2 by expression of the transcription factor Gata3 and exclusion of the T cell marker CD3 (Fig. 1B). Corroborating our 2-photon microscopy data, we observed that most pancreatic ILC2 positioned closely with IL-33^+^ cells, which also expressed podoplanin (Pdpn), a marker for fibroblasts (Fig. S1C) (1, 20), in the septal/capsular space (Fig. 1C, S1D). A smaller proportion of ILC2 could be detected in the adventitial cuff, parenchyma, or near the islets of Langerhans. Lastly, using 3D imaging of clarified pancreas, we similarly observed ILC2 in the capsular regions of the exocrine pancreas in untreated mice (Fig. 1D, S1E, Movie S1).

Flow cytometry profiling of untreated pancreata indicated that most IL-33^Cit+^ cells co-expressed Pdpn and the pan-fibroblast marker PDGFRα (CD140a) (Fig. 1E, 1F, S1F), thus confirming their fibroblast identity. A smaller percentage of IL-33^Cit+^ cells were CD45^-^CD31^-^Pdpn^high^CD140a^-^ mesothelial-like cells. IL-33 protein was largely sequestered to the nuclei of Pdpn^+^ cells, in line with its role as alarmin cytokine (Fig. S1G). We intravenously administered mice with anti-CD45.2-PE to profile the pancreatic tissue-resident immune compartment by flow cytometry (Fig. 1G, S1H). CD45^+^CD3^-^B220^-^NK1.1^-^ Lineage^-^CD127^+^Gata3^+^ ILC2 were almost exclusively tissue-resident. We also found a small fraction of tissue-resident Gata3^+^ Th2 cells (Fig. S1I); similarly, ILC2 were the major population of *Il13*^*tdTom+*^ cells in PBS-treated and IL-33-treated mice (Fig. S1J). Conversely, other circulating immune cells such as B cells readily stained for CD45.2. In terms of myeloid cells, we identified tissue-resident macrophages and dendritic cells (Fig. S1H); multiplex IF indicated that F4/80^+^Lyve-1^+^ macrophages (21) also inhabited the interstitium (Fig. S1K). Lastly, RORγt^+^ ILC3 were present in the pancreas-draining lymph nodes but almost absent in the pancreata of both PBS-treated and IL-33-treated mice (Fig. S1L). In summary, ILC2 comprise a tissue-resident immune population that co-localises with IL-33^+^ fibroblasts in a distinct anatomical niche of the exocrine pancreas at both steady-state or IL-33-driven inflammation (Fig. 1H).

### Topographical and transcriptomic analysis of the pancreas inferred niche-specific interactions between ILC2 and fibroblast subsets

**Fig. 2:**
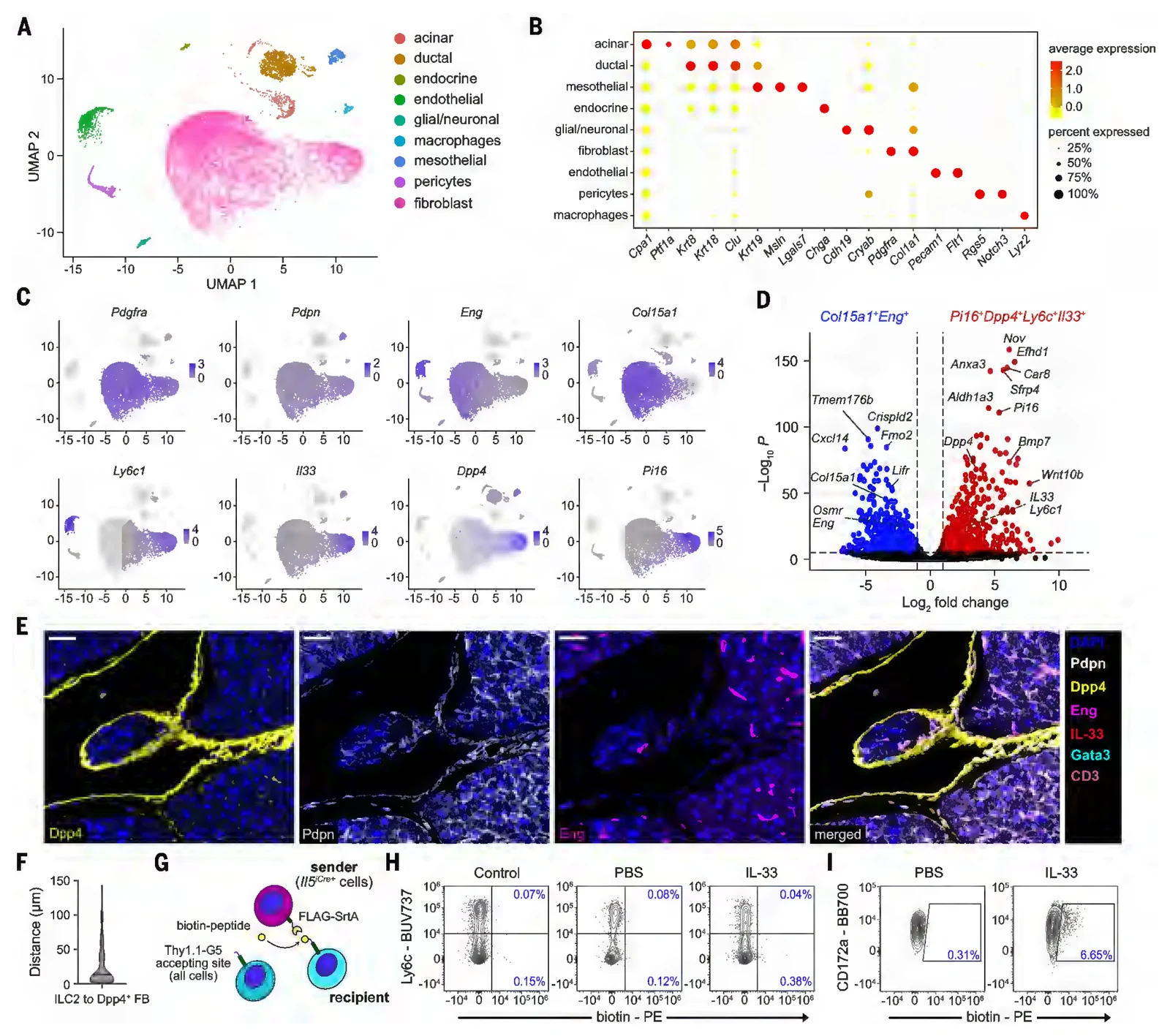
Topographical, cell-proximity, and transcriptomic analysis of the pancreas infer niche-specific interactions between ILC2 and fibroblast subsets. (**A-B**) Untreated C57Bl/6 mouse exocrine pancreata were flow-enriched for non-immune cells followed by droplet-based sc-RNA-seq (*17358 cells from 3 mice*). Uniform manifold approximation and projection (UMAP) dimensionality reduction revealed clusters of cells (**A**) that were annotated based on expression of lineage-specific genes (**B**). (**C-D**) Pancreatic fibroblasts universally expressed some genes (*Pdpn* and *Pdgfra*) but also revealed two sub-clusters that expressed *Pi16* and *Col15a1* respectively (**C**), amongst other differentially expressed genes (**D**). (**E-F**) Antibodies towards the protein products of differentially (*Dpp4, Il33* and *Eng*) and mutually (*Pdpn*) expressed genes were used to visualise both fibroblast subsets in FFPE sections of untreated pancreas using multiplex IF imaging; ILC2 (and T cells) were simultaneously detected by staining for Gata3 and CD3 (**E**). Distance between ILC2 and nearest Dpp4^+^Pdpn^+^ fibroblasts was quantified (**F**). (**G-I**) *Il5*^*tdTom-iCre/+*^R26^uLIPSTIC^ mice enable *in vivo* biotinylation of physically interacting cells in the pancreas which can be detected by flow cytometry (**G**). Control mice (R26^uLIPSTIC^), or PBS- or IL-33-treated *Il5*^*tdTom-iCre/+*^R26^uLIPSTIC^ mice were pulsed with biotin-peptide substrate, followed by flow analysis of pancreatic Pdpn^+^Pdgfra^+^ fibroblasts (**H**) or MHCII^+^CD11c^+^ dendritic cells (**I**) for biotin signal (*gating strategy in* Fig. S2H). Violin plots show data pooled from two independent experiments, lines represent quartiles (n=4 mice, **F**). Imaging data is representative of 2 independent experiments (**E**). Data is representative of 4 mice from 2 independent experiments (**H** and **I**). Scale bar: 50 μm (**E**).

To understand more about the potential symbiotic relationship between fibroblasts and ILC2 we performed single-cell RNA-sequencing (scRNA-seq) on non-immune cells enriched from the exocrine pancreas of untreated C57Bl/6 mice (Fig. S2A). Using unsupervised clustering we found 9 discrete populations (Fig. 2A). We annotated all known exocrine pancreatic non-immune cell-types, including epithelial acinar and ductal cells, endothelial cells, neuronal cells, mesothelial cells, pericytes, and fibroblast clusters (Fig. 2B). In line with our exocrine enrichment, we detected few endocrine cells. Focussing on *Pdpn*^*+*^*Pdgfra*^*+*^ fibroblasts, we observed two distinct sub-clusters that segregated based on the expression of *Pi16* and *Col15a1*, amongst other genes (Fig. 2C, Data S1). The *Pi16*^+^ fibroblast cluster is described in a pan-tissue fibroblast atlas as a putative precursor for more differentiated *Col15a1*^+^ fibroblasts (1). Our dataset indicated that *Pi16*^+^ fibroblasts also selectively expressed *Il33, Ly6c* and *Dpp4* (Fig. 2D). Conversely, we found that *Col15a1*^+^ fibroblasts co-expressed *Eng*, which encodes the co-receptor Endoglin (CD105), amongst other genes. Pdpn^+^Eng^+^ cells were observed throughout the parenchyma of the exocrine pancreas in untreated C57Bl/6 mice, whereas Pdpn^+^Dpp4^+^IL-33^+^ cells localised in the capsule and interstitium in close proximity to ILC2 (mean distance 20-30 μm) (Fig. 2E, 2F, S2B-S2D).

*Col15a1*^+^ and *Pi16*^+^ fibroblast subsets were both enriched in genes encoding cell-adhesion, ECM processes, and cell-migration signatures. *Pi16*^+^ fibroblasts showed specific associations with protein translation, tissue-repair and axonogenesis pathways, while *Col15a1*^+^ fibroblasts-specific pathways included ossification and angiogenesis (Fig. S2E). Moreover, *Col15a1*^+^ fibroblast expressed some genes related to retinol metabolism and storage (Fig. S2F), in line with recognised properties of pancreatic stellate cells (22); nevertheless, other stellate cell markers such as alpha smooth muscle actin (*Acta2*) and fatty acid binding protein 4 (*Fabp4*) were not highly expressed by fibroblasts in the pancreas at steady-state. Thus, *Col15a1*^+^ fibroblast likely represent mature fibroblasts of the pancreas while *Pi16*^*+*^ fibroblasts constitute a putative progenitor that co-localises with tissue-resident ILC2 in a discrete anatomical niche.

We next performed scRNA-seq on flow cytometry enriched CD45^+^CD3^-^B220^-^NK1.1^-^Lineage^-^CD127^+^ pancreatic ILCs from untreated C57Bl/6 mice (Fig. S2A). This ILC-enriched dataset also revealed ILC2 as the major ILC subset at steady-state. Using CellChat we inferred two-way communication networks between ILC2 and different cell-types of the exocrine pancreas (Fig. S3, S4A-S4C). Focussing on *Pi16*^+^ and *Col15a1*^+^ fibroblast subsets we found that ILC2 might engage both subsets in the healthy pancreas via numerous juxtracrine or paracrine mechanisms, including IL-6-family ligands (*Lif, Osm*), the EGFR-ligand amphiregulin (*Areg*), TGF-β superfamily ligands (*Tgfb1, Bmp7*), and macrophage inhibitory factor (*Mif*). Stereotypical ILC2-derived cytokine networks, such as *Il13*, were relatively weak in the steady-state pancreas. Conversely, *Pi16*^+^ fibroblasts were predicted to signal to ILC2 through extracellular matrix (ECM) components including fibronectin 1 (*Fn1*) and different collagens, as well as *Il33*, chemokines and other factors.

Multiplex IF analysis indicated that Fn1 was enriched in the interstitial extracellular matrix and localised in the same region as Dpp4^+^ fibroblasts, in physical association with ILC2 (Fig. S2G). We utilized *Il5*^*tdTom-iCre/+*^R26^uLIPSTIC^ mice (23) where *Il5*^*tdTom-iCre+*^ cells, which are primarily ILC2 in healthy control mice, expressed a bacterial sortase (SrtA) which enabled them to covalently transfer a biotinylated peptide tag to physically interacting recipient cells (Fig. 2G, S2H, S2I). We found that pancreatic ILC2 did not label many Ly6c^+^ or Ly6c^-^ fibroblasts, or other cell-types in PBS-treated control mice (Fig. 2H, S2H). Even during acute IL-33-driven inflammation, we observed no direct ILC2-fibroblast interactions, although CD172a^+^CD11b^+^ cDC2 acquired biotin signal (Fig. 2I, S2H).

### ILC2 influenced fibroblast homeostasis and promoted the *in-situ* expansion of putative *Pi16*^*+*^*Dpp4*^*+*^*Ly6c*^*+*^ fibroblast progenitors

**Fig. 3:**
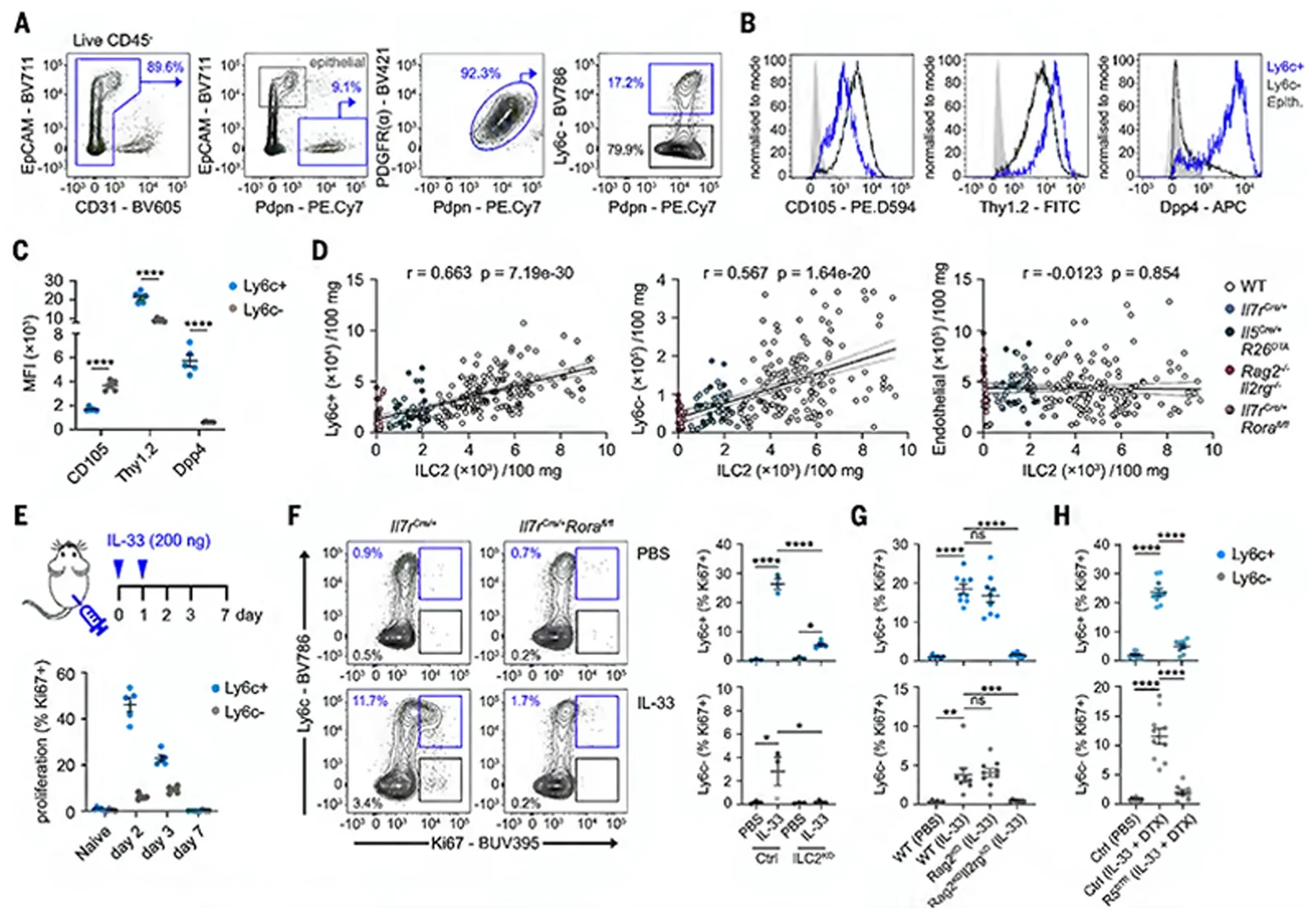
ILC2 influence steady-state fibroblast homeostasis and promote *in situ* expansion of putative Dpp4^+^Ly6c^+^ fibroblast progenitors. (**A-C**) Pancreata from uninflamed C57Bl/6 mice were analysed by flow cytometry to identify Ly6c^+^ and Ly6c^-^ fibroblast subsets (**A**), which were subsequently analysed for expression of the indicated surface markers. (**B**). Ductal epithelial cells were used as internal negative-staining representation (solid grey). Mean-fluorescence intensity (MFI) of these markers was measured on both subsets (**C**). **(D)** Uninflamed pancreata from the indicated mouse genotypes were analysed by flow cytometry to determine absolute numbers of ILC2, fibroblast subsets and endothelial cells. Correlation between ILC2 and non-immune cells was analysed as indicated. **(E)** C57Bl/6 mice were injected with PBS or IL-33 as indicated, followed by flow cytometry analysis of pancreatic fibroblast subsets for intracellular expression of Ki67. **(F)** Mice of the indicated genotypes were injected with PBS or IL-33, followed by flow cytometry analysis of Ly6c^+^ and Ly6c^-^ fibroblasts for intracellular Ki67 expression on day 3; representative dot plot shown left (percent of gated events), combined data for specific fibroblast subsets shown right. **(G)** Mice of the indicated genotypes were injected with PBS or IL-33, followed by flow cytometry analysis of Ly6c^+^ and Ly6c^-^ fibroblasts for intracellular Ki67 expression on day 3. **(H)** C57Bl/6 (Ctrl) and *Il5*^*tdTom-iCre/tdTom-iCre*^*Rosa26*^*lsl-DTR*^ (R5^DTR^) mice were injected with PBS or Diphtheria toxin (DTX) plus IL-33, followed by flow cytometry analysis of Ly6c^+^ and Ly6c^-^ fibroblasts for intracellular Ki67 expression on day 3. Dot plots show representative gating strategy; numbers indicate percent of gated cells. Additional gating strategy for (**A**) shown in Fig. S1F. Datapoints represent individual mice (**C**-**H**). Bar graphs indicate mean (±SEM). **C**. n=5 mice. Two-tailed student’s t-test. **D**. C57Bl/6 mice (white circles, n=150), *Il7r*^*cre/+*^ mice (light blue circles, n=29), *Il7r*^*cre/+*^*Rora*^*fl/fl*^ (light red circles, n=22), *Il5*^*tdTom-iCre*/+^*Rosa26*^*lsl-DTA/+*^ (dark blue circles, n=14), *Rag2*^*-/-*^*Il2rg*^*-/-*^ (dark red circles, n=10). Pearson correlation. Line of least-squares linear regression is overlaid (solid lines) with 95% confidence interval (dashed lines). **F**. n=3,3,3,3; data is representative of 2 independent experiments. Two-way ANOVA with Šidák’s multiple comparison test. **G**. n=6,9,9,9; data pooled from 2 independent experiments. **H**. n=9,10,8; data pooled from 2 independent experiments. One-way ANOVA with Tukey’s multiple comparison test. ns = not, * = p ≤ 0.05, ** = p ≤ 0.01, *** = p ≤ 0.001, **** = p ≤ 0.0001.

Based on our proximity labelling experiments, we hypothesized that tissue-resident ILC2 regulated a *Pi16*^*+*^*Dpp4*^*+*^*Ly6c*^*+*^ fibroblast progenitor niche not by cell-contacts, but primarily via secreted factors in the pancreas. We refined our flow cytometry panel to distinguish and investigate the different fibroblast subsets (Fig. 3A-C). Staining for additional surface markers, we identified both Ly6c^+^Dpp4^+^Thy1.2^high^ (*Pi16*^*+*^*Dpp4*^*+*^*Ly6c*^*+*^) and Ly6c^-^Dpp4^low^Thy1.2^+^CD105^high^ (*Col15a1*^*+*^*Eng*^*+*^*Ly6c*^*-*^) clusters in untreated C57Bl/6 mice; Ly6c^+^ fibroblasts also highly expressed IL-33^Cit+^ in reporter mice (Fig. S5A), while *Pi16* and *Col15a1* were expressed by flow sorted Ly6c^+^Dpp4^+^ or Ly6c^-^Dpp4^-^ subsets, respectively (Fig. S5B). Moreover, unsupervised clustering of flow cytometry data clearly distinguished the two fibroblast populations (Fig. S5C). Hence, we could accurately identify and interrogate pancreatic *Pi16*^*+*^*Dpp4*^*+*^*Ly6c*^*+*^ and *Col15a1*^*+*^*Eng*^*+*^*Ly6c*^*-*^ fibroblasts in mice using flow cytometry.

We quantified pancreatic ILC2 and fibroblasts in C57Bl/6 (wildtype), *Il7r*^*Cre/+*^*Rora*^*fl/fl*^ (ILC2-deficient) and *Il7r*^*Cre/+*^ (Cre-only control) mice (Fig. 3D). Pancreatic ILC2 were nearly absent in *Il7r*^*Cre/+*^*Rora*^*fl/fl*^ mice and reduced in *Il7r*^*Cre/+*^ compared to C57Bl/6 mice, likely due to reduced surface IL-7Rα expression (Fig. S5D), which is required for the maintenance of lymphocytes (24). We observed a strong positive correlation between the numbers of ILC2 and both *Pi16*^*+*^*Dpp4*^*+*^*Ly6c*^*+*^ (Ly6c^+^) and *Col15a1*^*+*^*Eng*^*+*^*Ly6c*^*-*^ (Ly6c^-^) fibroblasts in the pancreas at steady-state; conversely, there was no correlation between ILC2 and endothelial cell numbers (Fig. 3D, S5E). These findings were supported using alternative ILC2-deficient *Rag2*^*-/-*^*Il2rg*^*-/-*^ mice or ILC2-hypomorphic *Il5*^*tdTom-Cre/+*^*Rosa26*^*lsl-DTA*^ mice (Fig. 3D, S5E), indicating that ILC2 deficiency correlated with reduced fibroblasts densities. When considering myeloid cells in a correlation matrix, we also found effects of ILC2 numbers on dendritic cells and macrophages, as previously reported in other tissues (25, 26) (Fig. S5F, S5G). Overall, this suggested that ILC2 influenced steady-state fibroblast density.

We administered IL-33 to mice to activate ILC2. Pancreatic ILC2 expressed the IL-33 receptor (*Il1rl1*) (Fig. S2A) and upregulated IL-13 production after activation (Fig. S5H). Fibroblasts, which do not express *Il1rl1* (Fig. S5I), increased Ki67 staining, indicative of proliferation, after IL-33 administration (Fig. 3E). While Ki67 staining was observed in both Ly6c^-^ and Ly6c^+^ fibroblasts after IL-33 administration, there was more in the Ly6c^+^ fibroblast subset, especially at early time points. We did not observe Ki67 staining in endothelial cells but detected some staining in acinar and ductal cells (Fig. S5J). To assess if ILC2 were important for fibroblast cycling, we stimulated *Il7r*^*Cre/+*^*Rora*^*fl/fl*^ or *Il7r*^*Cre/+*^ mice with IL-33 (Fig. 3F). While IL-33 induced Ly6c^+^ fibroblast cycling in control mice, this effect was ablated in the absence of ILC2. To validate these results in different mouse models we used C57Bl/6 mice, *Rag2*^*-/-*^ mice, which lack adaptive lymphocytes but retain IL-33-responsive ILC2, and alymphoid *Rag2*^*-/-*^*Il2rg*^*-/-*^ mice (Fig. 3G); here, IL-33 administration rapidly induced similar cycling of Ly6c^+^ fibroblasts in C57Bl/6 and *Rag2*^*-/-*^ mice, while *Rag2*^*-/-*^*Il2rg*^*-/-*^ mice failed to respond. Lastly, Diphtheria toxin-mediated depletion of ILC2 in *Il5*^*tdTom-iCre/tdTom-iCre*^*Rosa26*^*lsl-DTR*^ mice also impaired fibroblast cycling compared to IL-33 treated controls (Fig. 3H, S5K).

Overall, these data indicated that ILC2 control fibroblast homeostasis, and mediate the acute effect of IL-33 on *Pi16*^*+*^*Dpp4*^*+*^*Ly6c*^*+*^ fibroblast proliferation in the exocrine pancreas.

### *Pi16*^*+*^*Dpp4*^*+*^*Ly6c*^*+*^ fibroblasts with progenitor capacity could trans-differentiate and repopulate the exocrine pancreatic fibroblast compartment

**Fig. 4:**
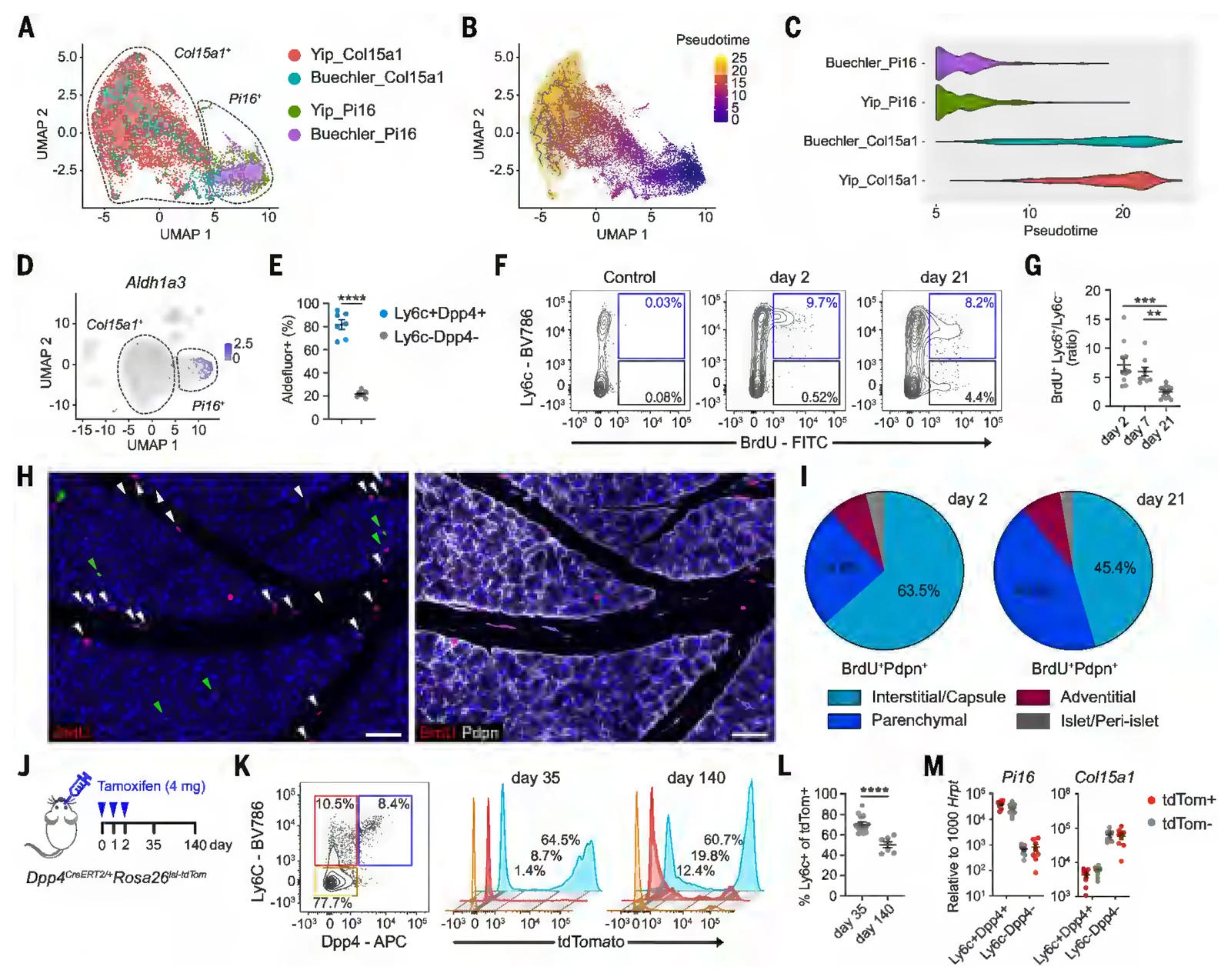
ILC2-induced Dpp4^+^Ly6c^+^ fibroblast progenitors differentiate and repopulate the intra-parenchymal fibroblast compartment. (**A-C**) Untreated pancreas fibroblast datasets from ourselves and Buechler et al. (1) were integrated and visualised using UMAP; previously identified *Pi16*^+^ and *Col15a1*^+^ sub-clusters in both datasets are indicated (**A**, *15946 cells*). Monocle pseudotime analysis was performed on the integrated dataset, showing trajectories in UMAP space (**B**), or in individual clusters in violin plots (**C**). (**D-E**) Aldehyde dehydrogenase 1 family member a3 (*Aldh1a3*) expression in the untreated pancreas (**D**); Aldehyde dehydrogenase activity was measured in different pancreatic fibroblast populations C57Bl/6 mice using Aldefluor assay (**E**). (**F-I**) C57Bl/6 mice were injected with IL-33 or PBS (Ctrl) and BrdU as indicated in Fig. S6C, followed by analysis of BrdU incorporation into Ly6c^+^ and Ly6c^-^ fibroblast subsets at the indicated time points (*representative dot plots shown*) (**F**). The ratio of BrdU^+^ subsets (Ly6c^+^ vs Ly6c^-^) was measured at the indicated time points (**G**). FFPE sections of pancreas from the day 2 and day 21 time point were stained for the indicated markers (*representative day 2 image shown; white - Interstitial/Capsular, green – Parenchymal BrdU*^*+*^*Pdpn*^*+*^ *cells*, **H**), followed by automated image analysis to quantify localisation of BrdU^+^Pdpn^+^ cells (**I**). (**J-M**) *Dpp4*^*CreERT2/+*^*Rosa26*^*lsl-tdTom*^ mice were treated with tamoxifen and sacrificed as indicated (**J**); the percent of tdTomato^+^ cells within the indicated pancreatic fibroblast subsets (**K**), or the percent of Ly6c^+^ cells amongst all tdTomato^+^ cells (**L**), was measured on days 35 and 140 *(Day 35 control groups were performed in the same batches as Fig. 5K)*. The indicated tdTomato^+^ and tdTomato^-^ fibroblast subpopulations were flow sorted from mice on day 35, followed by qRT-PCR for the indicated transcripts (**M**). Dot plots show representative gating strategy; numbers indicate percent of gated cells. Bar graphs indicate mean (±SEM). Data in (**F** and **K**) was pre-gated on live, CD45^-^Epcam^-^CD31^-^Pdpn^+^CD140a^+^ cells as in Fig 3A. Datapoints represent individual mice (**E, G, L** and **M**). **E**: n=7,7; data pooled from 2 independent experiments. Two-tailed Student’s t-test. **G**. n=11, 9, 13 pooled from 4 independent experiments. **L**. n=14, 7; data pooled from 6 independent experiments using male mice. Two-tailed Student’s t-test. **M**. n=8,8; data pooled from 2 independent experiments. * = p ≤ 0.05, ** = p ≤ 0.01, *** = p ≤ 0.001, **** = p ≤ 0.0001. Scale bar: 50 μm (**H**).

We next integrated our pancreatic fibroblast dataset with published data from a pan-organ atlas that identified putative *Pi16*^+^ ‘universal’ fibroblasts (1) (Fig. 4A). Our data closely overlapped, while pseudotime analysis also revealed that pancreatic *Pi16*^*+*^*Dpp4*^*+*^*Ly6c*^*+*^ fibroblasts may serve as precursors for *Col15a1*^*+*^*Eng*^*+*^*Ly6c*^*-*^ fibroblasts *in silico* (Fig. 4B, 4C). In our dataset, *Pi16*^*+*^*Dpp4*^*+*^*Ly6c*^*+*^ fibroblasts co-expressed the stem/progenitor cell maker aldehyde dehydrogenase 1 family member a3 (*Aldh1a3*) (27) and were preferentially labelled by fluorescent ALDH substrate *in vivo* over Ly6c^-^Dpp4^-^ fibroblasts (Fig. 4D, 4E, S6A, S6B). We used BrdU pulse-chase experiments to determine the fate of Ly6c^+^ fibroblasts, which co-expressed Pdpn and Pdgfra, after IL-33-induced proliferation (Fig. 4F, S6C, S6D). Mirroring Ki67 expression, we found that Ly6c^+^ fibroblasts selectively incorporated BrdU in response to IL-33 administration. At later time points, we found that BrdU^+^ fibroblasts downregulated Ly6c, indicative of transition towards *Col15a1*^*+*^*Eng*^*+*^*Ly6c*^*-*^ fibroblasts. After 3 weeks, a fraction of BrdU^+^ fibroblasts had transitioned from Ly6c^+^ to Ly6c^-^ (Fig. 4G). Using multiplex IF we found that BrdU mainly stained interstitial Pdpn^+^ fibroblasts at day 2. Moreover, we observed the appearance of BrdU^+^Pdpn^+^ fibroblasts in the exocrine pancreas parenchyma by day 21 (Fig. 4H, 4I); reduced BrdU^+^ fibroblasts ratios suggested their migration from the interstitium to the parenchyma over time (Fig. S6E).

We used *Dpp4*^*CreERT2/+*^*Rosa26*^*lsl-tdTom*^ mice to lineage-trace Dpp4^+^ fibroblasts; tamoxifen administration induced expression of tdTom in fibroblasts, which were primarily Dpp4^+^Ly6c^+^ at early time points (Fig. 4J, 4K, S6F). The percentage of tdTom^+^ Dpp4^-^Ly6c^-^ fibroblasts increased after 140 days and decreased the Ly6c^+^-to-Ly6c^-^ ratio (Fig. 4K, 4L). Moreover, *Pi16* expression decreased as cells transitioned from *Ly6c*^*+*^*Dpp4*^*+*^ into *Ly6c*^*-*^*Dpp4*^*-*^ cells, which instead expressed higher levels of *Col15a1* (Fig. 4M). Hence, we concluded that *Pi16*^*+*^*Dpp4*^*+*^*Ly6c*^*+*^ fibroblasts can act as bona fide fibroblast progenitors in the pancreas.

### ILC2 and macrophage-dependent mechanisms regulated *Pi16*^*+*^*Dpp4*^*+*^*Ly6c*^*+*^ fibroblast homeostasis

Given the known and inferred role of the ILC2-derived cytokine IL-13 on fibroblast activation, we tested *Il13*^*-/-*^ mice for IL-33-driven cell cycling (Fig. S7A). IL-13-deficiency partially reduced fibroblast proliferation for both Ly6c^+^ and Ly6c^-^ subsets, suggesting that other ILC2-derived factors could contribute. We excluded a role for IL-4 on fibroblast proliferation after IL-33 administration using a neutralising antibody (Fig. S7B). We found a similar reduction in fibroblast proliferation in *Nmur1*^*iCre*^*Il4/13*^*fl/fl*^ mice, in which ILC2s are deficient in IL-4 and IL-13, (Fig. S7C), implying an important role for ILC2-derived IL-13. We assessed fibroblast subsets under homeostatic conditions in *Il13*^*-/-*^ and C57Bl/6 mice, which revealed a reduction in both Ly6c^+^ and Ly6c^-^ fibroblast numbers, while endothelial cell numbers were comparable in both genotypes (Fig. S7D, S7E).

We used co-cultures of *ex vivo* purified pancreatic ILC2 and fibroblasts to interrogate direct crosstalk mechanisms (Fig. S7F, S7G). The addition of C57Bl/6-derived wildtype ILC2 resulted in an increase in cell-density over fibroblast monoculture, while co-culture with *Il13*^*-/-*^ ILC2 yielded an intermediate phenotype (Fig. S7G). We performed bulk-RNA-seq on purified fibroblasts to understand how ILC2 influenced fibroblast differentiation states via IL-13-dependent and -independent mechanisms and performed gene set enrichment analysis (GSEA) using published and our own signatures of *Pi16*^*+*^*Dpp4*^*+*^*Ly6c*^*+*^ fibroblasts (Fig. S7G-S7K, Data S2). The presence of ILC2 enriched for progenitor signatures, while monoculture skewed gene expression towards differentiated *Col15a1*^*+*^*Eng*^*+*^*Ly6c*^*-*^ fibroblast signatures. Comparison of wildtype and *Il13*^*-/-*^ ILC2 co-cultured fibroblasts indicated that IL-13 helped promote a *Pi16*^*+*^*Dpp4*^*+*^*Ly6c*^*+*^ fibroblast cell-state in *ex vivo* cultures (Fig. S7L).

Given that IL-33-mediated ILC2 activation induced proliferation of *Pi16*^*+*^*Dpp4*^*+*^*Ly6c*^*+*^ fibroblasts, we asked if chronic stimulation would result in fibrosis. Long-term dosing with IL-33 did not result in fibrosis of the exocrine pancreas, while flow cytometry revealed a reduction in absolute Ly6c^-^ fibroblast numbers (Fig. S7M, S7N). Indeed, we observed progressive reductions in absolute Ly6c^-^ fibroblast numbers on day 3 and 5 after IL-33 administration, while Ly6c^+^ fibroblast numbers were unchanged, or even slightly reduced, despite the large fraction of cycling cells (Fig. S7O). Hence, negative feedback mechanisms could locally counterbalance the pro-fibrotic effects of IL-33.

We used *Pdgfra*^*mGFP-CreERT2/+*^ Rosa26^uLIPSTIC^ mice to identify other cells that interacted with fibroblasts. Like *Il5-uLIPSTIC* mice, Pdgfra^Flag-SrtA+^ fibroblasts did not biotinylate ILC2 (Fig. S7P) but did biotinylate F4/80^+^MHCII^+/low^ macrophages in the pancreas after IL-33 administration (Fig. S7P, S7Q). Moreover, biotin-tagged F4/80^+^ macrophages increased GFP signal, which suggested the uptake of Pdgfra-GFP^+^ cells (Fig. S7R). More precise analysis in *Pdgfra*^*mGFP-CreERT2/+*^ mice indicated that Tim4^+^MHCII^+/low^ tissue-resident macrophages acquired more GFP signal (Fig. S7S). Depletion of tissue-resident Tim4^+^MHCII^+/low^ macrophages in the pancreas increased the number of proliferating and total Ly6c^+^ fibroblasts after IL-33 administration (Fig. S7T-S7V). This indicated that tissue-resident macrophages functioned to limit *Pi16*^*+*^*Ly6c*^*+*^*Dpp4*^*+*^ fibroblast numbers post-inflammation.

### IL-33 activated ILC2 indirectly constrained *Col15a1*^*+*^*Eng*^*+*^*Ly6c*^*-*^ fibroblast numbers

We next asked if ILC2 were involved in the effect of IL-33 on Ly6c^-^ fibroblasts. Three different ILC2-deficient mouse models, *Il7r*^*Cre/+*^*Rora*^*fl/fl*^, *Rag2*^*-/-*^*Il2rg*^*-/-*^, and *Il5*^*tdTom-iCre/tdTom-iCre*^*Rosa26*^*lsl-DTR*^ mice, did not show specific reduction in Ly6c^-^ fibroblasts following IL-33 treatment compared to their respective controls (Fig. S8A-S8C). Hence, while ILC2-derived IL-13 promoted *Pi16*^*+*^*Dpp4*^*+*^*Ly6c*^*+*^ fibroblasts, additional ILC2-dependent mechanisms constrained *Col15a1*^*+*^*Eng*^*+*^*Ly6c*^*-*^ fibroblasts under inflammatory conditions.

We used computational approaches to predict how ILC2 communicate with fibroblasts (Fig. S8D, S9). In addition to IL-13, we identified IL-6-family, TGF-β superfamily, CGRP, and EGFR cytokines as candidates. Exogenous IL-13 promoted growth in *ex vivo* pancreatic fibroblast monocultures; however, Areg, BMP2 or CGRP had no effect *in vitro* or *in vivo* (Fig. S8E-S8H). Also, LIGHT (*Tnfsf14*), which controls PI16^+^ reticular cells (28), did not influence fibroblast growth or expression of *Pi16* (Fig. S8F, S8I). Pancreatic ILC2 also secreted leukaemia inhibitory factor (*Lif*), oncostatin M (*Osm*) and IL-13 when stimulated with IL-33 and IL-7; notably, we detected low IL-13 production with only IL-7 stimulation (Fig. S8J). Recombinant OSM inhibited pancreatic fibroblast proliferation in monocultures and promoted apoptosis (Fig. S8E, S8K). Moreover, OSM receptor (*Osmr*) and LIF receptor (*Lifr*) transcripts were selectively expressed by *Col15a1*^*+*^*Eng*^*+*^*Ly6c*^*-*^ fibroblasts in the normal pancreas (Fig. S8L), and OSM or LIF administration reduced pancreatic Ly6c^-^ fibroblasts, while not affecting the Ly6c^+^ subset (Fig. S8M, S8N).

ILC2-specific deletion of *Lif* or germline *Osm* deletion failed to rescue IL-33-mediated reductions in Ly6c^-^ fibroblast numbers (Fig. S8O). Fibroblast-specific deletion of gp130 (required for OSM and LIF signalling) also did not rescue IL-33-mediated fibroblast reduction (Fig. S8P), although treatment with Tofacitinib (Jak-inhibitor) or SC144 (gp130 inhibitor) partially rescued Ly6c^-^ fibroblast reduction (Fig. S8Q). Moreover, unlike OSM, addition of LIF to *ex vivo* fibroblast cultures had no effect (Fig. S8R). From these data we reasoned that the effect of IL-33 and ILC2 on Ly6c^-^ fibroblasts is likely mediated by other cell-types that rely on gp130.

### ILC2 promoted the expansion of *Pi16*^*+*^*Dpp4*^*+*^*Ly6c*^*+*^ fibroblasts with progenitor potential in acute pancreatitis

**Fig. 5:**
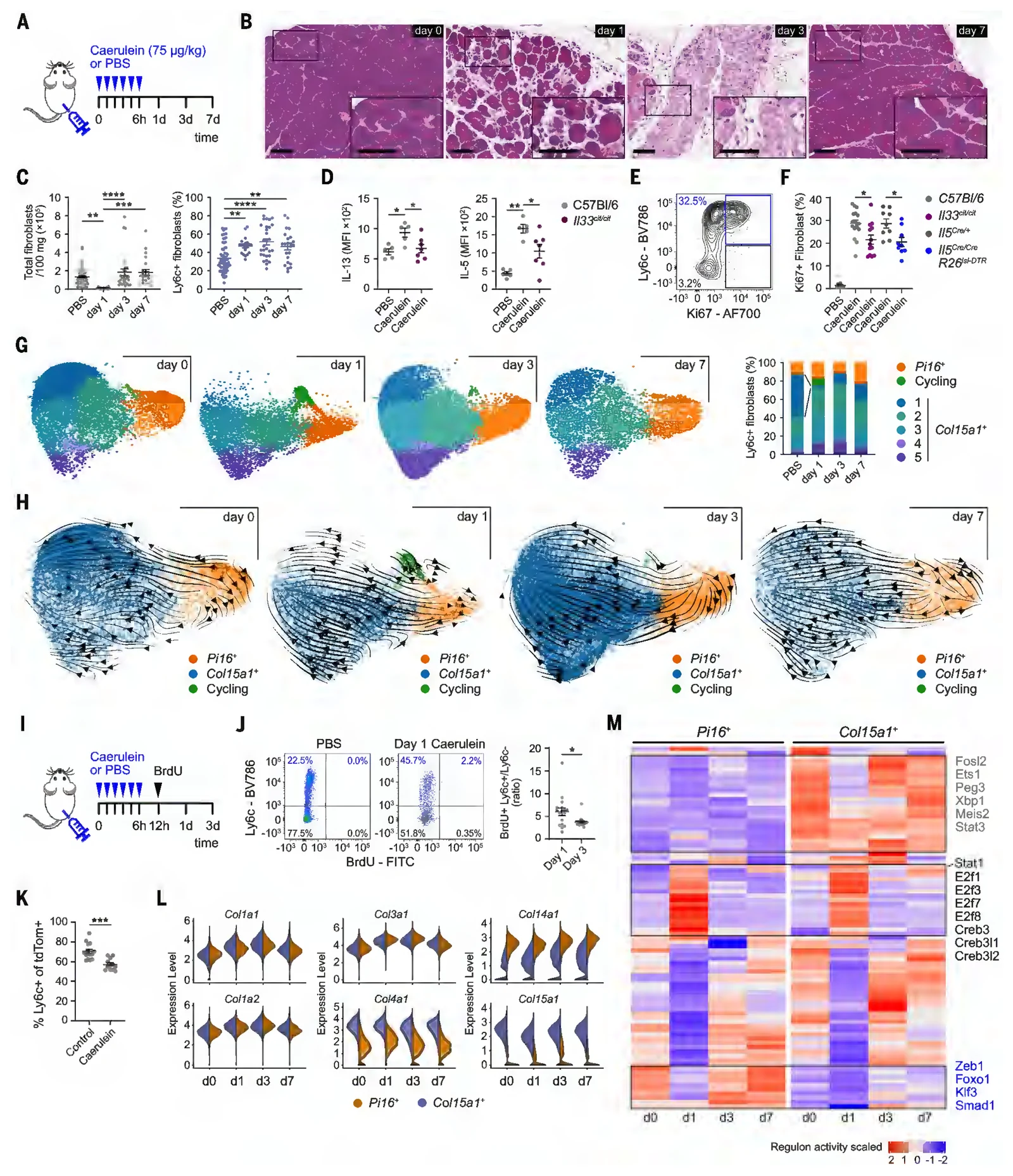
ILC2 promote the expansion of fibroblast progenitors in acute pancreatitis. (**A-C**) Mouse pancreas analysis at different time points after induction of acute pancreatitis (**A**). Representative haematoxylin and eosin (H&E) stained FFPE sections representing steady-state (0h), acinar cell necrosis and acute injury (day 1), acinar-to-ductal metaplasia (day 3), and resolution of tissue architecture (day 7) (**B**). Flow cytometry quantification of the absolute number of pancreatic fibroblast subsets at the indicated time points (**C**). (**D-F**) C57Bl/6 or *Il33*^*-/-*^ mice were injected with PBS or caerulein followed by detection of IL-13 and IL-5 mean fluorescence intensity (MFI) in ILC2 using intracellular flow cytometry at the 6 hour time point (**D**); similarly, at 24 hours we measured fibroblast proliferation by intracellular staining for Ki67 (**E**) which was quantified in the indicated genotypes and conditions (**F**). (**G-H**) Untreated or caerulein-treated C57Bl/6 mice were sacrificed at the indicated time points, followed by flow cytometry enrichment of non-immune cells and subsequent droplet based single cell transcriptomic analysis (*13779, 6572, 21627, 5424 cells respectively)*. RNA velocity analysis was performed on datasets from the indicated time points (**H**); similarly, with major cluster identities colour coded (**G**). (**I**-**J**) C57Bl/6 mice were treated with caerulein followed by administration of BrdU and sacrifice at the indicated timepoints (**I**). BrdU^+^ fibroblasts (*pre-gated as in* Fig. 6D) were identified by flow cytometry analysis (**J**); representative dot plots (left) indicating BrdU expression in Ly6c^+^ and Ly6c^-^ fibroblasts, which allowed quantification of ratios at day 1 and 3 (*right*). **(K)** *Dpp4*^*CreERT2/+*^*Rosa26*^*lsl-tdTom*^ mice were treated with tamoxifen on days 0, 1, and 2, with or without repeated caerulein administration on day 20 to induce acute pancreatitis and subsequent recovery for flow cytometry analysis of the percentage of Ly6c^+^ cells among tdTom^+^ fibroblasts on day 35 (*see Fig. S9G)*. **(L)** Expression of the indicated genes by *Pi16*^*+*^ and *Col15a1*^*+*^ fibroblast clusters were visualised at the indicated time points. **(M)** Regulome activities within the *Pi16*^*+*^ and *Col15a1*^*+*^ fibroblast clusters at the indicated time points were assessed using SCENIC analysis. Dot plots show representative gating strategy; numbers indicate percent of gated cells. Data in (**E** and **J**) was pre-gated on live, CD45^-^Epcam^-^CD31^-^Pdpn^+^CD140a^+^ cells as in Fig 3A. Datapoints represent individual mice (**C, D, F, J** and **K**). Bar graphs indicate mean (±SEM). **C**. n=61,20,29,23; data pooled from 15 independent experiments. One-way ANOVA with Tukey’s multiple comparisons test. **D**. n=5,5,7; data pooled from 2 independent experiments. One-way ANOVA with Tukey’s multiple comparisons test. **F**. n=11,17,13,9,9; data pooled from 5 independent experiments. One-way ANOVA with Tukey’s multiple comparisons test. **J**. n=17,18; pooled from 3 independent experiments. Two-tailed Student’s t-test. **K**. n=14,12; data pooled from 6 independent experiments using male mice. Two-tailed Student’s t-test. * = p ≤ 0.05, ** = p ≤ 0.01, *** = p ≤ 0.001, **** = p ≤ 0.0001. Scale bar: 50 μm.

IL-33 is sequestered in nuclei and is released in bioactive form upon non-apoptotic cell death (14). We hypothesized that IL-33 released following injury promotes ILC2-driven proliferation of the Ly6c^+^ fibroblast progenitor niche. Acute pancreatitis is a common and serious disease that can be modelled in mice by administration of the cholecystokinin analogue caerulein, causing overproduction and ectopic activation of digestive enzymes by acinar cells, and profound tissue necrosis on day 1 (Fig. 5A, 5B, S10A). Fibroblasts were ablated rapidly, but recovered as the exocrine pancreas underwent epithelial repair, marked by acinar-to-ductal metaplasia (day 3) and organ regeneration by day 7 (Fig. 5C, S10B). The fraction of Ly6c^+^ fibroblasts was higher during acute pancreatitis. We observed ILC2 activation 6 hours after induction of acute pancreatitis in C57Bl/6 mice, as determined by intracellular straining for IL-13 and IL-5, which was reduced in *Il33*^*-/-*^ mice (Fig. 5D). Fibroblast proliferation, observed largely in the Ly6c^+^ fraction on day 1, was also reduced in *Il33*^*-/-*^ compared to C57Bl/6 mice after caerulein administration (Fig. 5E, 5F). Similarly, temporal ILC2 depletion using *Il5*^*tdTom-iCre/tdTom-iCre*^*Rosa26*^*lsl-DTR*^ mice also impaired fibroblast proliferation (Fig. 5F, S10C).

scRNA-seq at different time points during acute pancreatitis damage, tissue-repair and resolution identified 7 fibroblast clusters in the integrated dataset, 5 of which represented subsets of *Col15a1*^*+*^*Eng*^*+*^*Ly6c*^*-*^ fibroblasts (Fig. 5G, S10D). The major *Col15a1*^*+*^*Eng*^*+*^*Ly6c*^*-*^ cluster rapidly contracted during acute pancreatitis. RNA velocity on fibroblasts at individual time points during acute pancreatitis revealed dividing *Pi16*^*+*^*Dpp4*^*+*^*Ly6c*^*+*^ fibroblasts that repopulated both the progenitor niche and *Col15a1*^*+*^*Eng*^*+*^*Ly6c*^*-*^ fibroblasts immediately after injury (Fig. 5H). Cell-cycling diminished by day 3, and *Col15a1*^*+*^*Eng*^*+*^*Ly6c*^*-*^ fibroblasts-cluster 2 represented a likely transitory state, as also indicated by intermediate expression of *Ly6c* (Fig. S10E). Pulsing mice with BrdU 12 hrs after caerulein administration labelled primarily Ly6c^+^ fibroblasts on day 1 (Fig. 5I, 5J). Subsequent analysis of BrdU^+^ fibroblasts on day 3 indicated an increase in the proportion of BrdU^+^ Ly6c^-^ fibroblasts (Fig. 5J); notably BrdU staining intensity decreased only amongst the Ly6c^+^ fibroblast population, suggesting that their differentiation into Ly6c^-^ fibroblasts likely accounted for the change in ratio (Fig. S10F). We obtained similar results using an alternative *Dpp4*^*CreERT2/+*^*Rosa26*^*lsl-RFP*^ lineage-trace model (Fig. 5K, S10G).

We observed dynamic induction of fibrosis-associated genes, including fibril forming Type I/III collagens, and *Acta2* (alpha smooth muscle actin, αSMA) in the *Col15a1*^*+*^*Eng*^*+*^*Ly6c*^*-*^ cluster on day 1 and 3 (Fig. 5L, S10E). Other collagens were enriched in *Pi16*^*+*^*Dpp4*^*+*^*Ly6c*^*+*^ fibroblasts, including *Col14a1*, which is involved in fibrillogenesis (29). We also observed a substantial increase in *Ccl2* and *Ccl7* transcript on day 1 only, while other chemokines such as *Ccl11, Cxcl1* and *Cxcl12* were constitutively expressed at all time points (Fig. S10E). These data suggested that an inflammatory, myofibroblast phenotype was dynamically regulated during tissue repair.

We performed SCENIC (30) transcriptional network analysis on both *Pi16*^*+*^*Dpp4*^*+*^*Ly6c*^*+*^ and *Col15a1*^*+*^*Eng*^*+*^*Ly6c*^*-*^ clusters at each time point (Fig. 5M, S10H). At steady-state *Pi16*^*+*^*Dpp4*^*+*^*Ly6c*^*+*^ fibroblast were enriched in stem/progenitor associated transcriptional networks (Zeb1, Foxo1, and Klf3) (31–34) and Smad1 (downstream of BMP signalling). Conversely, *Col15a1*^*+*^*Eng*^*+*^*Ly6c*^*-*^ fibroblasts were enriched in networks driven by Fosl2, Ets1 and Peg3 (fibroblast function (35–37)), Xbp1 (negative regulator of Foxo1 (38)), Meis2 (inverse relationship with BMPs (39)) and Stat3 (downstream of EGF- and IL-6-family cytokines). Both subsets rapidly downregulated their distinct regulatory networks early during acute pancreatitis and induced a shared transcriptional programme enriched in E2F-family members (regulation of cell-cycle, senescence, and apoptosis) (40), Creb3-family (ER-stress, protein secretion) (41), and Stat1 (downstream of interferons, IL-6-family, and IL-13Rα1/IL-4Rα (42) signalling). Both subsets largely pivoted back to their original regulatory networks by day 3, although Stat1-driven signatures were elevated in both subsets on day 7.

### ILC2-enforced fibroblast density established a set-point for acute injury-induced damage

**Fig. 6:**
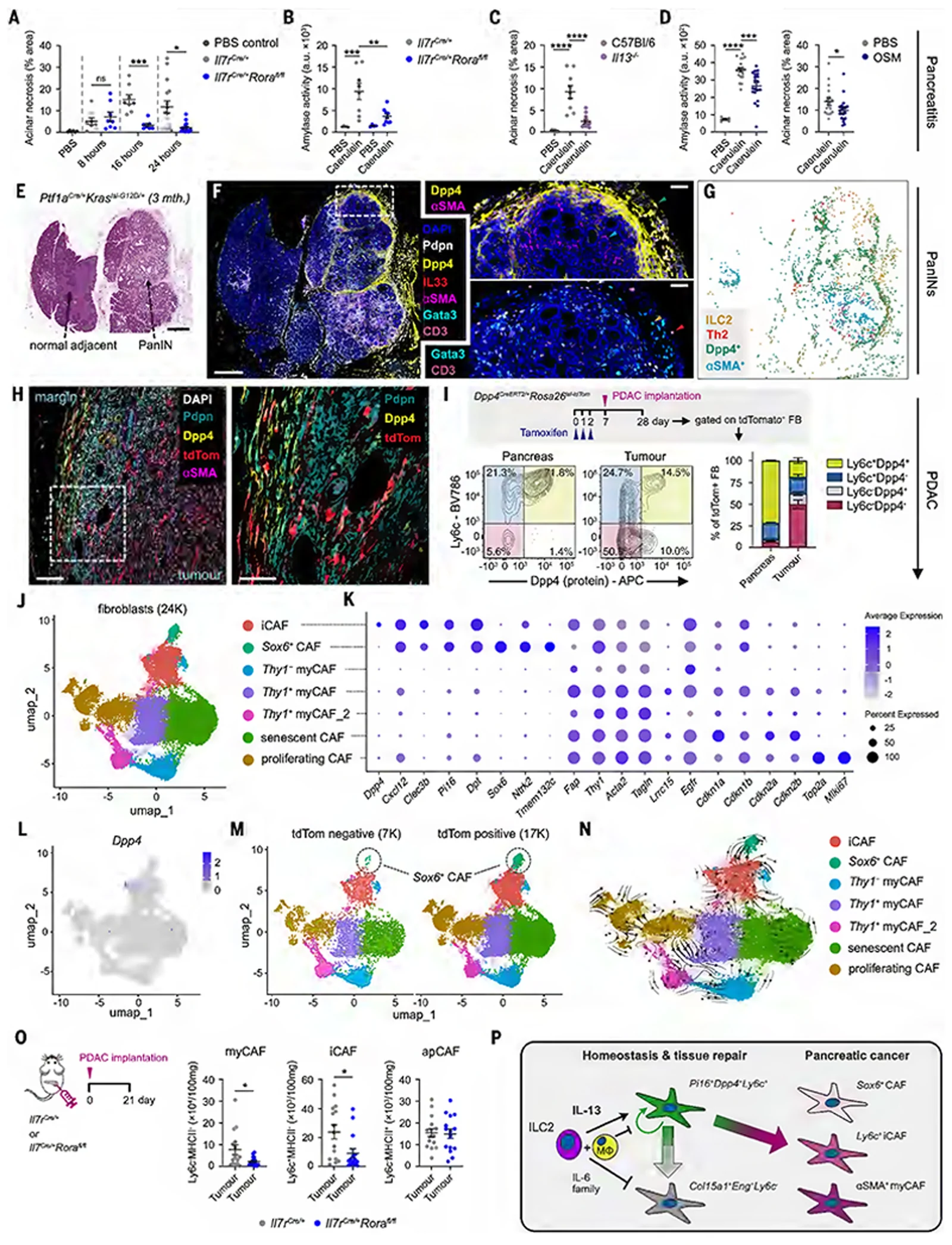
Steady-state fibroblast density set an inflammatory threshold for acute injury and serve as precursors for specific CAF subsets in pancreatic cancer. (**A-B**) Mice of the indicated genotypes were injected with PBS or caerulein, followed by histological assessment of acinar cell necrosis at the indicated time points (**A**) or analysis of serum amylase activity at 24 hours (**B**). **(C)** Mice of the indicated genotypes were injected with PBS or caerulein, followed by histological assessment of acinar cell necrosis at 24 hours. **(D)** C57Bl/6 mice were pre-treated with OSM or PBS at the indicated times (*See Fig. S11O)*, followed by administration of caerulein and assessment of pancreatic damage by serum amylase activity (*left*) or acinar necrosis by histological analysis (*right*). (**E-G**) Three-month-old *Ptf1a*^*Cre/+*^*Kras*^*lsl-G12D/+*^ (KC) mouse pancreas serial sections were stained for H&E (**E**), or for the indicated multiplex IF markers (**F**), which allowed identification of the indicated cell types (**G**). Scale bar: 200 μm (*left*), 50 μm (*right*). **(H)** *Dpp4*^*CreERT2/+*^*Rosa26*^*lsl-tdTom*^ mice were orthotopically implanted with KPC cells on day 0 and pulsed with tamoxifen on days 7, 8 and 9, followed by collection of tumour-bearing pancreata on day 21; tissues were assessed by multiplex IF microscopy for the indicated markers. Scale bar: 100 μm (*left*), 50 μm (*right*). **(I)** *Dpp4*^*CreERT2/+*^*Rosa26*^*lsl-tdTom*^ mice were tamoxifen-pulsed and implanted with KPC cells as indicated, followed by dissection of tumour and surrounding pancreas; tissues were assessed by flow cytometry and pre-gated for tdTomato-positive Pdpn^+^Pdgfra^+^ fibroblasts (FB, *gating strategy in* Fig. 3A). The indicated populations (*left*) and their relative proportions (*right*) are shown. (**J-N**) Tumours from orthotopically implanted, tamoxifen-pulsed *Dpp4*^*CreERT2/+*^*Rosa26*^*lsl-tdTom*^ mice were collected on day 21; droplet-based scRNA-seq was performed on flow enriched tdTomato-positive (*17384 cells from 4 hashed tumours*) and negative (*7044 cells from 4 hashed tumours*) fibroblast fractions. Uniform manifold approximation and projection (UMAP) dimensionality reduction on the combined fractions revealed clusters of cells (**J**) that were annotated based on expression of lineage-specific genes (**K**). *Dpp4* expression FeaturePlot (**L**). UMAP projections for tdTomato-positive and negative fractions (**M**). RNA velocity analysis was performed on the entire datasets (**N**). **(O)** Mice of the indicated genotypes were orthotopically implanted with KPC cells on day 0, followed by analysis of total numbers of CAF subsets by flow cytometry on day 21. **(P)** Graphical abstract. Bar graphs indicate mean (±SEM). Datapoints represent individual mice (**A-D** and **O**). **A**. n=6,8,8,8,7,12,11; data pooled from 2 independent experiments. Two-tailed Student’s t-test. **B**. n=5,8,4,9; data pooled from 2 independent experiments. Two-way ANOVA with Šidák’s multiple comparison test. **C**. n=9,9,10; data pooled from 2 independent experiments. One-way ANOVA with Tukey’s multiple comparisons test. **D**. n=5,20,20 (left), n=20,20 (right); data pooled from 3 independent experiments. One-way ANOVA with Tukey’s multiple comparisons test (left), two-tailed Student’s t-test (right). **O**. n=15,15; data pooled from 3 independent experiments. Two-tailed Student’s t-test. ns = not significant, * = p ≤ 0.05, ** = p ≤ 0.01, *** = p ≤ 0.001, **** = p ≤ 0.0001.

ILC2-deficient mice, which had fewer fibroblasts at baseline, showed less acinar cell necrosis and serum amylase activity (disease biomarker) compared to controls (Fig. 6A, 6B, S11A). Acute ILC2-depletion in *Il5*^*tdTom-iCre/tdTom-iCre*^*Rosa26*^*lsl-DTR*^ mice (which also lack expression of *Il5)* did not reduce acinar cell necrosis (Fig. S11B), indicating that acute ILC2 activation was disconnected from the tissue necrosis phenotype. ILC2 were important for early eosinophilia and monocyte recruitment after induction of acute pancreatitis. However, using antibody-depletion or eosinophil deficient mice we excluded a substantial role for these immune cells on acinar cell necrosis during acute pancreatitis (Fig. S11C-S11F). Macrophages, which as producers of IL-6, TNFα and IL-1β in the pancreas, could contribute to acinar cell necrosis (43). Nevertheless, *in vivo* neutralization of these cytokines individually, or TNFα and IL-1β combined, had no effect on disease severity (Fig. S11G, S11H). Granulocyte-macrophage colony stimulating factor (GM-CSF) deficient (*Csf2*^*-/-*^) mice presented with similar acinar cell necrosis compared to control animals, excluding potential ILC2-derived GM-CSF (Fig. S11I). Moreover, neutrophil depletion using anti-Gr-1 or anti-Ly6G, or depletion of T or NK cells was also ineffective at reducing acinar cell necrosis (Fig. S11F). Collectively, these data suggested that ILC2 influenced AP-dependent acinar cell necrosis via a mechanism unassociated with their canonical inflammatory role.

Besides IL-33, other DAMPs such as HMGB1 (*Hmgb1*) were highly expressed by pancreatic fibroblasts at rest (Fig. S11J); these or pathogen associated molecular patterns (PAMPs) are critical amplifiers of acinar cell necrosis during acute pancreatitis (43). Co-administration of bacterial lipopolysaccharide (LPS) with caerulein increased disease severity (Fig. S11K). Sparse detection of cleaved caspase 3 amongst necrotic acini indicated a disposition towards non-apoptotic cell death (44) (Fig. S11L). Acinar cells express Toll-like receptors, including *Tlr4* (45), and SCENIC analysis of acinar scRNA-seq data revealed induction of multiple NF-κB regulons early during acute pancreatitis (Fig. S11M, S11N, S12). This suggested that non-apoptotic fibroblast death can exacerbate early sterile inflammation-induced tissue necrosis in acute pancreatitis.

Hypothesizing that steady-state fibroblast densities could influence inflammation, we used *Il13*^*-/-*^ mice that also have reduced fibroblast numbers at baseline (Fig. S6D). Like ILC2-deficient mice, *Il13*^*-/-*^ mice were protected against acinar cell necrosis (Fig. 6C). We used OSM to specifically induce apoptotic depletion of Ly6c^-^ fibroblasts before administering caerulein to wildtype mice, which reduced both serum biomarkers and acinar cell necrosis (Fig. 6D, S11O). We used *Fap*^*DTR*^ mice, where Diphtheria toxin-induced reductions in total pancreatic fibroblasts before caerulein administration also reduced acinar cell necrosis (Fig. S11P). Conversely, *Il33*^*-/-*^ and wildtype mice had comparable baseline fibroblasts numbers, and similar levels of acinar necrosis during acute pancreatitis (Fig. S11Q, S11R). Notably, *Il33*^*-/-*^ mice showed impaired eosinophilia after caerulein administration (Fig. S11S), again discounting a substantial role for eosinophilia on acinar cell death. Thus, we concluded from complementary experimental approaches (Table S1) that fibroblast density at steady-state represented a sensor for sterile tissue damage.

### Pancreatic carcinogenesis triggered ILC2 - *Pi16*^*+*^*Dpp4*^*+*^*Ly6c*^*+*^ fibroblast niche expansion and differentiation into cancer associated fibroblasts.

Given that PDAC is associated with a highly fibrotic tumour microenvironment, we asked if ILC2 and *Pi16*^*+*^*Dpp4*^*+*^*Ly6c*^*+*^ fibroblasts were engaged during pancreatic tumourigenesis. Using *Ptf1a*^*Cre/+*^*Kras*^*lsl-G12D/+*^ (KC) mice, which develop pancreatic intraepithelial neoplastic (PanIN) lesions (46), we detected low-grade PanINs amongst normal adjacent pancreatic parenchyma by histological analysis (Fig. 6E, S13A). PanINs were encapsulated by a dense network of Dpp4^+^IL33^+^Pdpn^+^ fibroblasts, while αSMA^+^Pdpn^+^ fibroblasts, reminiscent of myCAFs, infiltrated the PanIN cores (Fig. 6F, 6G). ILC2 densities increased in the expanded Dpp4^+^ fibroblast niches that enveloped PanIN lesions, while Th2 cells were also readily detected (Fig. 6G). These data indicated that early tumourigenesis in KC mice was marked by a profound expansion of Dpp4^+^IL33^+^Pdpn^+^ fibroblasts and ILC2, while the presence of Th2 cells suggested the establishment of an adaptive type-2 immune polarised niche as has been reported in inflamed neonatal skin (47).

While antigen presenting (ap)CAFs in PDAC can arise from mesothelial cells, the origin of myCAFs and inflammatory (i)CAFs remains unclear (18). Hypothesizing that steady-state *Pi16*^*+*^*Dpp4*^*+*^*Ly6c*^*+*^ fibroblasts contribute to CAF ontogeny, we orthotopically implanted *Pdx1*^*Cre*^*Kras*^*lsl-G12D/+*^*Trp53*^*lsl-R172H/+*^ (KPC) mouse-derived pancreatic tumour cells (48) into *Dpp4*^*CreERT2/+*^*Rosa26*^*lsl-tdTom*^ mice, which were pulsed with tamoxifen early after surgery to label Dpp4^+^ fibroblasts (Fig. S13B). Histological and IHC analysis of the resultant tumours revealed that Dpp4 lineage-derived tdTom^+^ cells formed dense networks that enveloped and infiltrated the tumour mass (Fig. S13C, S13D). Using multiplex IF staining, we found that tdTom^+^ cells expressed Pdpn while Dpp4 protein was mainly co-expressed at the edge of the tumour margin, or in surrounding unaffected interstitial or capsular niches, but lost on tdTom^+^ cells within the tumour core (Fig. 6H, S13E). Moreover, enhanced expression of αSMA in tumours suggested the conversion into myCAFs.

We found, by flow cytometry, that while most Dpp4 lineage-traced Pdpn^+^Pdgfra^+^ fibroblasts retained Dpp4 and Ly6c protein expression in the surrounding pancreas, the majority of tumour-resident cells downregulated these markers (Fig. 6I, S13F). We also traced *Pi16*-expressing fibroblasts by subcutaneously implanting KPC-derived tumour cells into *Pi16*^*iCre/+*^*Rosa26*^*lsl-YFP*^ mice (Fig. S14A-S14J). While cutaneous YFP^+^ cells predominantly expressed Dpp4 and Ly6c under steady state conditions, these cells gave rise to the majority of LRRC15^+^ myCAFs (49), which also exhibited transcriptional changes associated with trans-differentiation from *Pi16*^*+*^*Dpp4*^*+*^*Ly6c*^*+*^ fibroblasts (Fig. S14K, S14L). Hence, we concluded that *Pi16*^*+*^*Dpp4*^*+*^*Ly6c*^*+*^ fibroblasts served as a local progenitor pool for specific CAF subsets.

We performed scRNA-seq on tdTom-positive and negative fibroblast fractions from orthotopic tumours in *Dpp4*^*CreERT2/+*^*Rosa26*^*lsl-tdTom*^ mice (Fig. S13G). Unsupervised clustering of the integrated dataset revealed different CAF subsets, including previously described iCAFs, myCAFs, and a distinct mesothelium-derived apCAF cluster (17) (Fig. S13G, 6J, 6K). We also found two CAF clusters that expressed transcripts associated with senescence or high levels of the transcription factor *Sox6*, amongst other genes. tdTom-positive cells downregulated *Dpp4* transcript and could differentiate into iCAF and myCAF lineages (Fig. 6L, 6M). Moreover, *Sox6* CAFs derived selectively from Dpp4 lineage-traced fibroblasts (Fig. 6M, S13H). We computationally inferred differentiation trajectories using RNA velocity (Fig. 6N) and CellRank (Fig. S13I) (50), a method that incorporates both RNA processing and cell-state dynamics, which indicated that *Dpp4*^*+*^ fibroblasts were a prominent precursor of iCAF, myCAF and *Sox6*^+^ CAFs (Fig. S13J).

Given the relationship between ILC2 and *Pi16*^*+*^*Dpp4*^*+*^*Ly6c*^*+*^ fibroblasts, we investigated how ILC2 influenced CAF biology in pancreatic cancer. Orthotopic implantation of KPC cells into *Il7r*^*Cre/+*^*Rora*^*fl/fl*^ mice, lacking ILC2, showed a reduction in myCAF and iCAF numbers compared to those in *Il7r*^*Cre/+*^ mice (Fig. 6O). Mesothelial-derived apCAF numbers were unchanged. These data indicate that the ILC2-*Pi16*^*+*^*Dpp4*^*+*^*Ly6c*^*+*^ fibroblast niche is locally engaged early during pancreatic tumourigenesis, and both differentiates and contributes to the CAF compartment in PDAC.

## Discussion

We found that *Pi16*^*+*^*Dpp4*^*+*^*Ly6c*^*+*^ fibroblast can differentiate into both *Col15a1*^*+*^*Eng*^*+*^*Ly6c*^*-*^ fibroblasts, which resemble pancreatic stellate cells at steady-state, and distinct CAF lineages in pancreatic cancer. As with other ‘stem like’ compartments *Pi16*^*+*^*Dpp4*^*+*^*Ly6c*^*+*^ fibroblasts inhabited a defined anatomical niche that was also enriched in ILC2. We found that ILC2 were important for maintaining steady-state fibroblast numbers in the pancreas. While tonic production of IL-13 contributed to this function in an IL-33-independent manner, ILC2 also served as sensors of insults to the tissue fibroblast compartment. IL-33-dependent activation of ILC2 reinforced the *Pi16*^*+*^*Dpp4*^*+*^*Ly6c*^*+*^ fibroblast progenitor niche to replenish tissue fibroblast numbers, representing a prototypical homeostatic circuit. The ILC2 - *Pi16*^*+*^*Dpp4*^*+*^*Ly6c*^*+*^ fibroblasts niche expanded profoundly around PanIN lesions, indicating a capacity to sense and respond to early pancreatic carcinogenesis. Delineation of bona fide differentiation relative to trans-differentiation and phenotypic elasticity of *Pi16*^*+*^*Dpp4*^*+*^*Ly6c*^*+*^ fibroblasts remains to be fully understood. Moreover, the functions and mechanistic underpinning(s) of *Pi16*^*+*^*Dpp4*^*+*^*Ly6c*^*+*^ fibroblasts in other organs and contexts is of substantive interest.

DAMPs alert surrounding cells of tissue damage, exemplified by necrotic cell death driven sterile inflammation (51); our data now posits that necrosis of tissue fibroblasts represents a potent early source of DAMPs that are sensed by both immune and non-immune cells. As previously shown with adipocytes (52), our data are consistent with fibroblast density setting an important tissue-tone for inflammation. Indeed, obesity and fibrosis are risk factors, or amplifiers, of tissue damage (53).

Dialogue within tissue niches is greatly influenced by cell proximity and contact. Although ILC2 and *Pi16*^*+*^*Dpp4*^*+*^*Ly6c*^*+*^ fibroblasts did not physically interact, Tim4^+^ tissue-resident macrophages contacted fibroblasts during inflammation and constrained the number of rapidly dividing *Pi16*^*+*^*Dpp4*^*+*^*Ly6c*^*+*^ fibroblasts under inflammatory conditions, likely via phagocytosis. While ILC2-derived IL-13 promoted early *Pi16*^*+*^*Dpp4*^*+*^*Ly6c*^*+*^ fibroblast proliferation, ILC2 constrained *Col15a1*^*+*^*Eng*^*+*^*Ly6c*^*-*^ fibroblast numbers, possibly through indirect mechanisms involving IL-6-family cytokines and tissue-resident macrophages. This functional dualism further illustrates the central homeostatic role of ILC2 on the pancreatic fibroblast niche, with important ramifications on acute tissue injury and tissue homeostasis.

Pancreatic ILC2 are described in the anatomically distinct islets of Langerhans (19, 54) and perivascular niche (13), although these represent a fraction of total pancreatic ILC2. While pancreatic ILC2 represent a relatively minor population of immune cells in untreated mice, we find that the ILC2-*Pi16*^*+*^*Dpp4*^*+*^*Ly6c*^*+*^ fibroblast niche expanded around pre-neoplastic lesions. Notably, ILC2 are reported to have pro- or anti-tumour roles in pancreatic cancer (55, 56), indicating our incomplete understanding of ILC2 in the pancreas. Nevertheless, pathologies of the exocrine pancreas, like chronic pancreatitis and pancreatic ductal adenocarcinoma are marked by stromal reactions that promote disease progression (17, 18, 49). We find that *Pi16*^*+*^*Dpp4*^*+*^*Ly6c*^*+*^ fibroblasts contribute to a substantial fraction and subsets of CAFs in PDAC and determining how the ILC2-fibroblast circuit is engaged throughout disease progression is of outstanding interest.

## Materials and Methods

### Animals

*Dpp4*^*CreERT2*^ mice (57) (provided by Prof. Seale), *Pdgfra*^*mGFP-CreERT2*^ mice (58) (provided by Prof. Acton), *Osm*^*-/-*^ mice (59) (provided by Prof. Jorgensen), *Il6st*^*fl/fl*^ mice (60) (provided by Prof. Muller), *Il5*^*tdTom-iCre*^ mice (61) (JAX, 030926), *Nmur1*^*iCre-eGFP*^ mice (62) (JAX, 038197), *FAP*^*DTR*^ mice (63) (provided by Prof. Fearon), *Rosa26*^*lsl-DTR*^ mice (JAX, 008040) *Rosa26*^*lsl-DTA*^ mice (JAX, 009669), *Rosa26*^*fl-G5-Thy1.1-fl-FLAG-SrtA*^ ‘universal LIPSTIC’ mice (23) (JAX, 038221), *Rosa26*^*lsl-tdTom*^ mice (JAX, 007914), *Ptf1a*^*Cre/+*^*Kras*^*lsl-G12D*^ mice (provided by Prof. Brindle), *Lif*^*fl/fl*^ mice (64), *Il33*^Cit/Cit^ (65) and *Il13*^tom/tom^ mice (66) (provided by Prof. McKenzie), *Areg*^-/-^ mice (provided by Prof. Clatworthy), *Il7r*^Cre/+^ mice (67) (provided by Prof. Rodewald), *Il7r*^Cre/+^*Rora*^fl/fl^ mice (*Rora*^fl/fl^ provided by Prof. McKenzie (68)), *Rag2*^-/-^ mice (Charles River), and *Rag2*^-/-^*Il2rg*^-/-^ mice (69) (provided by Prof. DiSanto), *Gata1*^*tm6Sho/J*^ (JAX, 005653) mice, all on the C57Bl/6 background, were maintained in the Cancer Research UK – Cambridge Institute (CRUK-CI) animal facility under specific-pathogen-free conditions. *Pi16*^*IresCre*^*Rosa26*^*lsl-eYFP*^ mice were maintained at the University of Toronto under specific-pathogen-free conditions (see below). C57Bl/6 background *Csf2*^*-/-*^ mice were maintained at University of Zurich Laboratory Animal Sciences Center under specific-pathogen-free conditions. Wild-type C57Bl/6J mice were purchased from Charles River Laboratories. Animal work was conducted under project licenses PD7484FB9 or PF7993249 at the CRUK-CI (with approval from the Cancer Research UK - Cambridge Institute, Animal Welfare Ethical Review Body) all in accordance with UK Home Office regulation, or with approval from the cantonal veterinary office of Zurich (ZH054/2022 and ZH111/2022), or approval from the Animal Care Committee at the Centre for Phenogenomics (TCP) and the University of Toronto. Mice were sex and age matched whenever possible, and most mice were used at 8-14 weeks of age. All mice were humanely euthanized by rising CO_2_ exposure and cervical dislocation as confirmation of death. Mice housed at the University of Toronto were kept in specific-pathogen free cages on a 13:11 hour light-dark cycle and were temperature controlled at ~22°C. Mice housed at the CRUK-CI were kept at in specific pathogen-free, individually ventilated cages on a 12h light/dark cycle, 55% ± 10% humidity, and were temperature controlled at ~21 ± 2 °C. Mice were kept on a standard diet with ad libitum water (unless indicated otherwise for specific procedures) with nesting material and cage enrichments provided. Mice were allowed to acclimate for at least 1 week in the animal facility before the beginning of any experiment.

### Generation of *Pi16*^*IresCre*^ knock-in mice

C2 ES cells were targeted using standard protocols to generate the Pi16<tm1(icre/ERT2)Tcp> allele (MGI:7466219) (67). The target vector included 3-kb 5’ (chr17:29543925 to 29546924, mm39) and 7-kb 3’ (chr17:29546925 to 29553924, mm39) homology arms with a knock-in sequence comprised of an internal ribosomal entry site (IRES) and the iCre/ERT2 CDS with an SV40 polyadenylation signal and an FRT-flanked PGK-neo selection cassette between rox sites located between the iCre open reading frame and the ERT2 open reading frame. This was designed so that prior to Dre-mediated recombination, a constitutively active iCre is expressed under control of the *Pi16* transcriptional regulatory elements. We refer to this cassette as CreX-neo-ERT2. Quality control of the ES cells was done by short- and long-range PCR and quantitative PCR to assess copy number of the targeting cassette (Neo, ThermoFisher Mr00299300_cn) and chromosomes 1 (ThermoFisher Mm00037405_cn), 8 (ThermoFisher Mm00256539_cn), 11 (ThermoFisher Mm00004107_cn) and Y (ThermoFisher Mm00527282_cn) to assess ploidy. Sequencing of homology arm-genome junctions of long-range PCR (5lr and 3lr) and full short-range PCR (sr1 and sr2) amplicons completed QC (Table S2).

Chimeras were generated by aggregation with CD-1 (70) diploid embryos and embryo transfer to in-house bred CD-1 pseudopregnant recipients (71) following published protocols. Chimeras were bred to C57BL/6NBrdCrl-Tyr<c-Brd> (Charles River 493) and black pups (evidence of ES cell genome transmission) were genotyped with short-range PCR and quality controlled using long-range PCR and neomycin copy number. To remove the neomycin selection cassette, sperm from quality-controlled B6N-Pi16<tm1(icre/ERT2)Tcp> was used for *in vitro* fertilization with C57BL/6NCrl oocytes. Zygotes with polar bodies were electroporated in 400 ng/μL Flp mRNA (Miltenyi BIotec Inc. 130-106-769), cultured overnight, and 2-cell embryos transferred into in-house bred pseudopregnant CD-1 mice. Born pups were genotyped by PCR to confirm the neo cassette was removed. The resulting allele Pi16<tm1.1(icre)Tcp> (MGI:7466221), nicknamed CreXERT2, has the IRES and iCre open reading frame followed by a rox-flanked SV40 polyA signal (STOP) after the stop codon of the *Pi16* open reading frame. This constitutively active Cre is expressed under control of the *Pi16* promoter without concern about interference from the ACTB promoter driving the neomycin resistance gene. Two founder lines were generated (E3 and D4) and crossed to *Rosa26*^lsl-YFP^ reporter animals. Both founders elicited robust YFP expression in the skin. All experiments solely used the E3 founder line.

### In vivo procedures

Recombinant (rm) IL-33 (0.2 μg, Biolegend), rmOSM (0.5 μg, Biolegend), rmAREG (0.5 μg, Biolegend), BrdU (2 mg, Thermo Scientific) were administered by intraperitoneal injection in 200 μl of PBS as indicated. Labelling of circulating leukocytes to evaluate tissue-residency was achieved by i.v. injection of 3 μg anti-CD45.2-PE (clone 104, Biolegend) antibody 3 min prior to sacrifice. Tamoxifen (Sigma-Aldrich) was prepared in 10% ethanol (Sigma-Aldrich) and 90% corn oil (Sigma-Aldrich) at 20mg/mL, dissolved by sonication, and administered at 4 mg by oral gavage at the indicated time points. Lineage tracing experiments of Dpp4^CreERT2^ reporter mice were performed in matched experimental batches. For acute pancreatitis experiments, mice were given subcutaneous analgesia of 50 μg/kg buprenorphine before hourly injections of 75 μg/kg Caerulein (Sigma) or endotoxin-free PBS for 6 hours. For experiments involving depletion of Tim4^+^ macrophages alongside IL-33 administration, mice were administered 250 μg anti-CSF1R antibody (clone AFS98; BioXCell #BE0213) via intraperitoneal injection on days -4, -2, and 1, and 200 ng rm-IL-33 on days 0 and 1, followed by sacrifice for flow cytometry analysis on day 3 as indicated. For experiments involving pharmacological inhibition of IL-6 family pathways, tofacitinib (Stratech) or SC144 (Selleck Chemicals) were administered at 200 μg daily from the start of IL-33 administration. For diphtheria-toxin mediated depletion experiments, diphtheria toxin (Sigma-Aldrich) was administered at the indicated doses.

### Subcutaneous tumour model

6–10-week-old *Pi16*^*IresCre/+*^*Rosa26*^*lsl-YFP*^ were inoculated subcutaneously in the left unilateral flank with 1×10^5^ KPC (a generous gift from T. McGaha) tumour cells in a 1:1 suspension of PBS and Geltrex™ Flex LDEV-Free Reduced Growth Factor Basement Membrane Matrix (Gibco, A4000046701). Tumours were removed 21-23 days after implantation.

### Orthotopic tumour model

For orthotopic allografts, mice were anaesthetized with isoflurane and an incision was made in the left abdominal area to expose the pancreas. 10 μl of 1:1 PBS and Matrigel cell suspension (2.5×10^4^ cells) was injected into the tail of the pancreas. PDAC cell-lines (6419c5 or 2838c3, (48)) were originally derived from C57Bl/6 background *Pdx1*^*Cre*^*Kras*^*lsl-G12D/+*^*Trp53*^*lsl-R172H/+*^*Rosa26*^*YFP/YFP*^ (KPCY) mice. The peritoneal membrane was sutured with absorbable Vicryl Rapide suture (Ethicon, W9913) and the skin was closed with tissue glue (GLUture, Zoetis) and wound clips (FST, 12022-09). Mice were given pre- and post-surgical analgesia of meloxicam (2 mg/ml) and buprenorphine (0.03 mg/ml), and wound clips were removed one-week post-surgery.

### LIPSTIC

For studying of physical cell-cell interactions by proximity labelling using *Il5*^*tdTom-icre/+*^*Rosa26*^*uLIPSTIC/+*^ and *Pdgfra*^*eGFP-CreERT2/+*^*Rosa26*^*uLIPSTIC/+*^ mice, 7-12-week-old mice were administered tamoxifen and/or IL-33 at the indicated time points. Mice were intraperitoneally administered with 100 μL 20 mM Biotin-Ahx-LPETGS-NH2 (LifeTein) every 20 minutes for 2 hours prior to sacrifice, and mice sacrificed 20 minutes after the final dose.

### Histology

Tissues were fixed in 10% neutral buffered formalin and embedded in paraffin before sectioning into 4 μm slices. Sample embedding, sectioning, and hematoxylin and eosin staining were conducted by the CRUK-CI Histology Core. Mouse CK19 (clone TROMA-III, DSHB) staining was performed on Leica’s automated Bond-III platform. Acinar necrosis was quantified on H&E sections using Halo Software (Indica Labs).

### Single cell preparation

#### Pancreas

Pancreata were weighed, then mechanically dissociated, followed by digest in 5 ml of HBSS containing collagenase I (375 U/ml), DNase I (0.15 mg/ml) and Soybean Trypsin inhibitor (Sigma, 0.05 mg/ml) for 30 minutes at 37°C on a shaker (220 rpm), followed by dissociation on a syringe and needle, filtration through a 70 μm strainer and RBC lysis.

#### Lymph node

Cell suspensions were prepared by mechanical dissociation, followed by digest in 0.5 ml of RPMI-1640 containing collagenase I (375 U/ml) and DNase I (0.15 mg/ml) for 45 min at 37°C on a shaker (1100 rpm), followed by red blood cell (RBC) lysis.

### Flow cytometry

Single cells were incubated with anti-mouse CD16/32 (Thermo Fisher) to block Fc receptors. For intracellular staining we used the Foxp3/Transcription Factor Kit (Thermo Fisher). For cytokine staining we used the BD Cytofix/Cytoperm kit (BD) after re-stimulation of single cell suspensions for 2h at 37C with PMA/ionomycin in the presence of protein transport inhibitors (eBioscience Cell Stimulation cocktail). Data was acquired on a BD Fortessa, BD Symphony, or Cytek Aurora instrument. Cells were quantified using CountBright beads. Data was analysed using FlowJo X (Tree Star).

The following antibodies were used in this study, with clones, venders, and fluorochrome as indicated: Thy1.2 (30-H12, eBioscience, FITC), CD140a (APA5, BV421 (BioLegend), PE (BioLegend), Super Bright 702 (Invitrogen)), CD31 (390, BV605 (BioLegend), Pacific Blue (BioLegend), BUV737 (eBioscience)), CD31 (MEC13.3, BioLegend, MEC13.3), EpCAM (G8.8, BioLegend, BV711, PE-Cy7), Ly6c (HK1.4, BV785 (BioLegend), BV421 (BioLegend), PE-Cy7 (eBioscience), BUV737 (BD)), Sca-1 (D7, BioLegend BV711), DPP4 (H194-112, APC (BioLegend), PE/Cy7 (BioLegend)), CD105 (MJ7/18, BioLegend, PE, PE/Dazzle 594), CD133 (315-2C11, BioLegend, PE/Dazzle 594), Podoplanin (8.1.1, PE/Cy7 (BioLegend), APC/Fire 750 (BioLegend), APC (BioLegend), Super Bright 600(Invitrogen)), Ki67 (B56, BD, BUV395), Ki67 (SolA15, eBioscience, AF700), BrdU (BD, FITC), Biotin (Bio3-18E7, Miltenyi Biotec, PE), CD8 (53-6.7, PerCP-eFluor710 (eBioscience), BUV395 (BD), BB700 (BD)), CD45 (30-F11, BV510 (BioLegend), PE-Cy7 (BioLegend), BUV395 (eBioscience)), NK1.1 (PK136, BUV395 (BD), BUV661 (Invitrogen)), CD4 (RM4-5, eBioscience, AF700), B220 (A3-6B2, eBioscience, APC-eFluor780), CD19 (6D5, BioLegend, Spark Blue 550), CD127 (SB/199, BD, PE-CF594, APC), KLRG1 (2F1, BioLegend, BV605), ST2 (RMST2-2, eBioscience, PE), Gata3 (TWAJ, eBioscience, eFluor660), Foxp3 (FJK-16s, eBioscience, AF488, APC, PerCP-eFluor 710), RorgT (Q31.378, BD, BV650), CD172α (P84, AF488 (BioLegend), BB700 (BD)), Siglec-F (1RNM44N, eBioscience, SB600), Siglec-F (S17007L, BioLegend, BV421), XCR1 (ZET, BioLegend, BV650), CD64 (X54-5/7.1, Biolegend, BV711), CD11b (M1/70, Biolegend, BV785, Spark NIR 685), I-A/I-E (CI2G9, BD, BUV395, BV480, BB700), CD11c (N418, AF700 (eBioscience), Spark UV 387 (BioLegend)), F4/80 (BM8, APC-eFluor780 (eBioscience), PE/Fire 810 (BioLegend)), Ly6G (1A8-Ly6g, PE-eFluor610 (eBioscience), BUV805 (BD)), Tim4 (RMT4-54, BB700 (BD), PerCP-eFluor710 (eBioscience)), Thy1.1 (OX-7, BioLegend, PerCP), LRRC15 (M25, Genentech, unconjugated). Lineage cocktail contained CD5 (53-7.3), CD19 (1D3), CD11b (M1/70), CD11c (N418), FcεR1α (MAR-1), F4/80 (BM8), Ly-6C/G (Rb6-8C5), and Ter119 (TER-119), all on eFluor450 (eBioscience). Dead cells were excluded with the fixable viability dye UV455, LIVE/DEAD Fixable Blue, or eFluor780 (eBioscience).

### ILC2s and fibroblast isolation and co-culture

Primary fibroblasts were isolated by fluorescence-activated cell sorting (FACS) on Live, CD45^-^EpCAM^-^CD31^-^Pdpn^+^Pdgfra^+^ fibroblast populations with a BD FACSAria II cell sorter. For ILC2 cultures, ILC2s were first expanded *in vivo* by intraperitoneal injection of 200 ng rmIL-33 on day 0, 1, 6, and 7, and pancreata harvested on day 9. ILC2s were isolated by FACS, gating on Live, CD45^+^CD3^-^ Lineage^-^CD127^+^KLRG1^+^ cells (Lineage = CD5, CD19, F4/80, Gr-1, CD11b, CD11c, Ter119, NK1.1, B220, FcεR1α).

For fibroblast monocultures *in vitro*, fibroblasts were seeded at an initial density of 20,000 cells/well in 48-well flat bottom plates in DMEM/F-12 supplemented with 10% FCS plus penicillin and streptomycin (P/S) and allowed to adhere for 48 hours. Subsequently, culture media was washed and replaced with DMEM supplemented with 10% FCS plus P/S. For cultures with cytokines, fibroblasts were cultured in cell culture media containing rmIL-13 (Peprotech), rmAREG (BioLegend), rmBMP-2 (BioLegend), recombinant rat CGRP (Cambridge Bioscience), rmLIGHT (BioLegend), rmLIF (BioLegend), and rmOSM (BioLegend), at concentrations indicated in respective figures. Growth kinetics were monitored on the Incucyte S3 System (Sartorius) using phase confluence as a readout, and growth normalised to initial phase confluence. Detection of phosphatidylserine exposure in apoptotic cell death was performed using the Incucyte Annexin V Red Dye (Sartorius). For ILC2 monocultures *in vitro*, ILC2s were isolated as described above and seeded at 10000 cells/well in 96-well U-bottom plates in RPMI supplemented with 10% FCS plus P/S.

For ILC2-fibroblast cocultures, fibroblasts were isolated as described above and allowed to adhere in DMEM-F12 supplemented with 10% FCS for 48 hours prior to coculture. Media was washed off, and ILC2s added to fibroblast cultures at a density of 2000 cells/well. Coculture media was supplemented with 10% FCS and 10 ng/ml rmIL-33 (BioLegend). Growth kinetics were monitored on the Incucyte S3 System (Sartorius) using phase confluence as a readout.

For culture of fibroblast obtained from *Pi16* reporter mice, tissues were pooled from 2-3 mice and digested in 15-minute intervals in 5 ml of digest mix, as described above. Cells from digested adipose (subcutaneous) and skin were plated in a 6-well plate at 6e5-1e6 cells per well (Corning, CLS3526). Cells from digested adipose (epididymal), lung, lymph node, and pancreas were plated at 5e6-20e6 cells per 100 mm dish (Greiner Bio-One, 07-001-017). All cells were plated in DMEM (Gibco, 11995065) supplemented with 10% FBS (D-10). Cells were washed with PBS after 24 hours to remove debris and non-adherent cells. D-10 was replenished every 3-5 days. Cells were detached using 0.25% Trypsin-EDTA (Gibco, 25200056) for 5 minutes at 37°C prior to passaging at 80% confluency or for flow cytometry at indicated time points.

### In vitro assays

ELISA was used for quantification of IL-13, LIF, and OSM concentration in cell culture supernatants (R&D Biosystems), in accordance with manufacturer’s instructions. Quantification of serum amylase activity was performed using a colorimetric amylase assay kit (Abcam).

### Microscopy

For imaging of whole pancreas explants, *Il13*^*tdTom/+*^*Il33*^*Citrine/+*^ mice were injected with 200 ng rmIL-33 intraperitoneally for 2 days and sacrificed 3 days after the first injection. One day prior to sacrifice, mice were injected intraperitoneally with Hoechst 33342 (100 μg, Thermo Scientific). CellMask Deep Red (10μg, Invitrogen) was injected intravenously 30 minutes prior to sacrifice to label membranes of vasculature and acinar cell parenchyma. Pancreas explants were imaged on an inverted laser-scanning confocal microscope (Stellaris 8, Leica Microsystems) with an attached pulsed Coherent Chameleon Ultra II laser, using a 25X 0.95NA water immersion objective lens. Hoechst, citrine, and tdTomato were excited at 2-photon excitation wavelengths of 800 nm, 950 nm, and 1040 nm respectively.

Multiplex immunofluorescence imaging was performed on formalin-fixed paraffin-embedded sections using Opal multiplex IHC kits (Akoya Biosciences), following manufacturer instructions. Paraffin-embedded sections were deparaffinised through xylenes and rehydrated through decreasing graded alcohol. Deparaffinised sections were then subject to antigen retrieval using 10 mM sodium citrate (pH 6) or TRIS-EDTA (pH9) buffers, depending on the primary antibody used. Before primary antibody incubation, tissue sections were blocked with blocking/antibody diluent (ARD1001EA) for 20 min at RT. Subsequently, primary antibodies were incubated at working dilutions, durations, and temperatures described in (Table S3). After washing, HRP-conjugated secondary antibody was then incubated for 15 minutes (anti-rabbit and anti-rat polymer-HRP) or 30 minutes (anti-Syrian Hamster-HRP). Sections were washed prior to addition of Opal reagents (1:100 in Opal amplification buffer).

Multiplex immunofluorescence sections were imaged on an inverted laser-scanning confocal microscope (Stellaris 8, Leica Microsystems) using a 25X 0.95NA water immersion objective lens. Large sections of tissue were imaged for downstream analysis as a maximum intensity projection (typically a z-stack of 10 μm, 40-60 tiles). Image analysis was performed using the HALO software (Indica Labs). Cell types were identified using the HighPlex-FL algorithm and fine-tuned for cell types of interest detailed in the main text. Regions of pancreatic tissue were manually annotated into interstitial, parenchymal, islet, and vascular territories based on histological appearance using DAPI and podoplanin channels to delineate structural boundaries.

For experiments involving immunofluorescence and imaging of *Pi16* reporter mice, mouse samples were submitted to TCP Pathology Core for histology and immunohistochemistry. The samples were processed in a TissueTek VIP 6 tissue processor (Sakura Finetek), paraffin-embedded and 5 μm-thick sections were collected onto charged slides (Assure, Epic Scientific). The sections were deparaffinized in a TissueTek Prisma instrument (Sakura Finetek), submitted to heat-induced epitope retrieval with citrate buffer pH 6.0 (10mM Sodium Citrate, 0.05% Tween-20) and incubated in a pressure cooker for 10 minutes, followed for 30 minutes at room temperature. The sections were incubated in Protein Block (Agilent, X090930-2) to minimize non-specific antibody binding before each primary antibody incubation.

Primary antibodies goat anti-GFP (1:500, Abcam, ab6673) and rabbit anti-CD31 (1:100, Abcam, ab28364) were diluted in Antibody Diluent solution (Agilent, S302283-2) and incubated sequentially. Slides were incubated overnight in anti-GFP primary antibody at 4°C. On the following day, the tissue sections were washed in 1x TRIS-buffered saline with 0.1% Tween-20 (TBST), then incubated in anti-goat IgG conjugated with Alexa Fluor 647 (Invitrogen, A32849) diluted at 1:200 in Antibody Diluent for 1 hour at room temperature. After rinsing in TBST, the sections were incubated in Protein Block prior to overnight incubation in anti-CD31 primary antibody overnight at 4°C. On the following day, tissue sections were washed in TBST, incubated in anti-rabbit IgG conjugated with Alexa Fluor 555 (Invitrogen, A32732) diluted at 1:200 in Antibody Diluent for 1 hour at room temperature, rinsed in TBST then counterstained with DAPI (Sigma, D9542). Tissue autofluorescence was quenched in Sudan black B saturated solution in 70% ethanol (Sigma, 199664) for 25 minutes at room temperature, then coverslips were mounted with Vectashield Vibrance anti-fade mounting medium (Vector, H-1700). Slides were scanned at 20X using an Olympus VS120 slide scanner equipped with a Hamamatsu ORCA-R2 C10600 digital camera (Evident).

### Cleared pancreatic thick slices and imaging analysis

Untreated C57BL/6 mice were intracardially perfused with cold PFA 4% in PBS. Digestive systems were collected and post-fixed in PFA 4% overnight followed by three washes in PBS; then pancreases were dissected. Organs were embedded in agarose 4% and cut as 500 μm slices in a vibrating blade microtome. Slices were dehydrated (10 min consecutive baths of 20%, 40%, 60%, 80%, 100% methanol in PBS), bleached (5% hydrogen peroxide in methanol) for 1 h and then hydrated (10 min consecutive baths of 100%, 80%, 60%, 40%, 20% methanol in PBS, followed by a bath of PBS). Slices were incubated for 1h in permeabilization solution (0.2% Triton X100, 23g/L glycine, 20% DMSO in PBS), blocked overnight in blocking buffer (0.2% Triton X100, 5% donkey serum, 5% goat serum, 10% DMSO in PBS) and incubated overnight in primary antibodies (Table S4) diluted in 5% DMSO, 3% donkey serum in PtwH solution (0.2% Tween-20, 10 mg/L heparin in PBS). Tissues were washed 4 times (30 min) in PTwH solution, incubated overnight in secondary antibodies diluted in 3% donkey serum in PTwH, followed by 4 more washes in PTwH solution. Permeabilization, blocking and antibody incubations were done at 37°C in agitation. For their clarification, slices were dehydrated, incubated in 66% dichloromethane diluted in methanol for 3h, and then incubated in 100% dichloromethane for 10 min for their delipidation. Slices were finally incubated in ethyl cinnamate overnight for refractive index adjustment. Cleared slices were mounted in a glass slide covered by a coverslip with two coverslips acting as spacers on each side; ethyl cinnamate was used as mounting media and the coverslips were sealed to the glass slide with a melted mix of paraffin:vaseline (50:50).

Sections were imaged as Z-stacks with a 20X multi-immersion objective (glycerol as immersion medium) in a confocal Stellaris 8 microscope (Leica). 3D volumes were analysed using the software Imaris (10.2.0). The parenchymal and interstitial volumes were automatically reconstructed based on tissue autofluorescence of the green and red channels by using the ‘surface’ tool. ‘Spots’ tool was used to automatically identify all GATA3^+^ cells; GATA3^+^ cells were then filtered based on CD3 expression as positive or negative by using the ‘intensity sum of square’ value for CD3 channel. GATA3^+^CD3^-^ cells were reconstructed as ILC2s, GATA3^+^CD3^+^ cells were not reconstructed. ILC2s were automatically stratified as parenchymal (light pink) or interstitial (cyan) based on whether their 3D location was within the reconstructed parenchymal or interstitial volumes.

### Bulk-RNA-seq

For bulk RNA-sequencing of fibroblasts recovered following coculture, fibroblasts and ILC2s were detached by incubation at 37°C with 0.25% trypsin. Fibroblasts were then sort-enriched, gating on Live, CD45-Pdpn+Pdgfra+ cells. RNA was extracted using RNeasy Plus Micro kit (Qiagen) and 10 ng RNA used as input material for library preparation. Libraries were generated with the NEBNext® Single Cell/Low Input RNA Library Prep Kit for Illumina (NEB) according to manufacturer’s instructions. The pooled libraries were quantified with KAPA Library Quantification Kit for Illumina (Kapa Biosystems) and sequenced (paired end 50nt) on an Illumina NovaSeq 6000 system (Illumina).

For bulk RNA-sequencing of pancreatic ILC2s isolated from untreated and IL-33-treated mice, pancreatic ILC2s were sorted as live, CD45+, CD3-, B220-, Lineage (CD5, CD11b, CD11c, Gr-1, Ter119, CD19, F4/80, FcεR1α)-, NK1.1-, CD127+, KLRG1+ cells. RNA was extracted using the RNeasy mini kit (Qiagen, 74104) and DNAse was eliminated using the Turbo DNase kit according to manufacturer’s instructions (ThemoFisher, AM1907). Libraries were generated with the NEBNext® Single Cell/Low Input RNA Library Prep Kit for Illumina (NEB) according to manufacturer’s instructions. Library quantification and quality check of the libraries were done using the Bioanalyser DNA 1000 kit (Agilent Technologies, 5067-1505) and the KAPA library quantification kit for Illumina (KAPA Biosystems, KK4828) respectively. The libraries were then pooled and sequenced on 2 lanes of the HiSeq 4000 system (single end 50nt).

The quality of raw sequence data was assessed using FastQC (v 0.11.9). Raw sequence was trimmed for adapter content using Trimmomatic (v 0.39) and aligned to the GRCm38.p6 genome using STAR (v 2.7.7a). Quantification of gene expression was performed using Salmon. Differential gene expression analysis was carried out using DESeq2175 (v 1.34.0) in R (v 4.1.2). 1 sample each from the wildtype and IL-13 coculture groups were excluded based on high expression of ILC2 transcripts, including *Il13*, indicating ILC2 contamination among sorted fibroblasts retrieved from coculture.

Subsequently, a heatmap of differentially expressed genes was created using the ComplexHeatmap package176 (v2.15.2), filtering on differentially expressed genes (padj < 0.05) with a minimum expression threshold of 200 DESeq2 normalised counts. Gene set enrichment analysis was performed using the fgsea package177 (v1.24.0). Gene signatures of Pi16+ and Col15a1+ fibroblasts were obtained from Buechler et al. (1) (as reported in the original publication in Supplementary table 4; Markers_Col15a1vsPi16 and Markers_Pi16vsCol15a1).

Predictions of receptor-ligand interactions from bulk RNAseq data were performed using the CellChat (72) package (v1.6.1) using default parameters except where otherwise described. As CellChat was developed for use in single-cell applications, over-expressed genes were defined as genes for which at least one sample has a normalised expression count exceeding 500. Computation of communication probability was also performed using the ‘truncatedMean’ method, with trim=0 and raw.use = TRUE, and filterCommunication set at min.cells=1.

For bulk RNA-sequencing of fibroblasts of subcutaneously implanted KPC tumour cells, tumours were isolated and digested using 0.1 mg/ml DNase I (Roche, 10104159001), 0.8 mg/ml Dispase II (Gibco, 17105041), and 0.2 mg/ml Collagenase P (Roche, 11249002001) in RPMI with 2% FBS. Tissues were digested in 3 ml of digestion mix for 8 minutes at 37°C after which cell suspension was removed and filtered through a 70 μM cell strainer (Miltenyi Biotec, 130-110-916) into cold PBS with 2% FBS and 2 mM EDTA. An additional 3ml of digestion mix was added to remaining tissue and incubated for 12 minutes before cell suspension was removed as before. A final 3 ml of digestion mix was added to tissue for a final 15-minute incubation prior to the removal and filtration of remaining cell suspension. ACK lysis buffer (Gibco, A1049201) was used to lyse red blood cells. Cells were counted using a ViCell XR (Beckman Coulter), prior to staining for flow cytometry or Fluorescent Activated Cell Sorting (FACS). Cells were gated as live, CD45-CD24-CD31-EpCAM-PDPN+ and then YFP+LRRC15+, YFP+LRRC15- or YFP-LRRC15+ and sorted by FACS. RNA was isolated using RNeasy Plus Mini Kit (Qiagen, 74136). Quantity and quality of RNA was assessed using an RNA nano chip on an Agilent Bioanalyzer (Agilent). Sequencing libraries were prepared using trueseq stranded total RNA library preparation kits with RiboZero as per the manufacturer’s instructions (Illumina). Samples were sequenced on an Illumina NextSeq2000 sequencer for 40 million paired end reads with a sequencing depth at read lengths 59bp x 59bp. Protein-coding genes were retained for downstream analysis using a custom list of mouse protein-coding genes generated from NCBI. Differential gene expression analysis was performed using the DESeq2 package (v.1.49.3). A regularized log (rlog) transformation was applied to normalized counts, and sample-level variability was assessed by principal component analysis (PCA). Gene set enrichment analysis (GSEA) was conducted using the clusterProfiler (v.4.17.0) and fgsea (v.1.35.6) R packages. Normalized enrichment scores from GSEA were visualized in custom plots using ggplot2 (v.3.5.2).

### Single-cell RNA-seq analysis

For single-cell RNA sequencing data of non-immune cells over the course of acute pancreatitis, acute pancreatitis was first induced in mice via administration of caerulein as described above. Mice were sacrificed and pancreata harvested at 24 hours, 3 days, and 7 days following the induction of acute pancreatitis. Cell suspensions were sort-enriched on live (Live/Dead Fixable eF780-), nucleated (DRAQ5+), non-haematopoetic (CD45-) cells. For single-cell RNA sequencing of ILCs from untreated mice, cell suspensions were sort-enriched on live, CD45+, CD3-, B220-, Lineage (CD5, CD11b, CD11c, Gr-1, Ter119, CD19, F4/80, FcεR1α)-, CD127+, NK1.1- cells. Single-cell encapsulation was performed using the Chromium Single Cell B Chip Kit (10x Genomics, PN-1000073), followed by library preparation using the Chromium Single Cell 3’ GEM Library & Gel Bead Kit v3 (10x Genomics, PN-1000075). Pooled libraries were then sequenced (paired end 50nt) on a NovaSeq 6000 system.

Initial mapping and counting of 10x Genomics scRNA-seq reads was performed using Cell Ranger v3.1.0. Data was then imported into Seurat (73) for downstream analysis. For scRNA-seq of the non-immune compartment, quality control was performed on individual datasets, filtering for single cells with between 1000 and 6000 expressed genes, and with less than 10% expressed mitochondrial transcripts. Doublets were identified and excluded using the scDblFinder package (74). Subsequently, datasets were integrated in Seurat, and principal components 1 to 15 were provided as input for dimensionality reduction via UMAP with default parameters in Seurat. Cells were clustered using the Louvain method on a shared nearest neighbour graph using default parameters (resolution = 0.3).

Differentially expressed genes for Col15a1+ and Pi16+ fibroblast clusters were identified by first obtaining pseudobulk gene expression for each cluster on a per-sample level (AggregateExpression), then performing differential gene expression testing using the DESeq2 package. Data was visualised as a volcano plot using the EnhancedVolcano package. The top 500 upregulated or downregulated genes ranked by fold-change in gene expression were then used for functional gene ontology enrichment analysis with the clusterProfiler (v4.6.2) package, using the Molecular Signatures Database (MSigDB) C2:Biological Processes gene sets.

For scRNA-seq of ILCs in the untreated pancreas, initial quality control was performed by filtering for single cells with less than 5% expressed mitochondrial transcripts. Data were then log-normalised, and principal components 1 to 25 were provided as input for dimensionality reduction via UMAP with default parameters in Seurat. Cells were clustered using the Louvain method on a shared nearest neighbour graph using default parameters (resolution = 0.2).

For scRNA-seq of lineage traced fibroblasts of *Dpp4*^*CreERT2/+*^*Rosa26*^*lsl-tdTom/lsl-tdTom*^ mice, mice were orthotopically implanted with 2838c3 PDAC tumour cells. Mice were administered 4mg of tamoxifen by oral gavage on days 7-9 and sacrificed on day 21. Tumour single-cell suspensions were sort-enriched on live (Live/Dead Fixable eF780-), CD45-, EpCAM-, CD31-, Pdpn+, Pdgfra+ cells, and separately sorted into tdTomato+ and tdTomato- sort fractions per tumour. Individual sort fractions were loaded for on-chip multiplexing (GEM-X OCM 3’ Chip Kit v4 4-plex), single-cell encapsulation, and library preparation following manufacturer’s instructions (GEM-X Universal 3’ Gene Expression v4 4-plex Reagent Kits). Pooled libraries were then sequenced (paired end 50nt) on a NovaSeqX system. A custom reference was constructed with the GRCm39-2024-A reference genome containing an appended entry for tdTomato and the WRPE of the Ai9 mouse using cellranger mkref, and reads aligned, demultiplexed, counted using cellranger multi (Cell Ranger v9.1.0). Data was subsequently imported into Seurat, subsetting for cells with fewer than 10% mitochondrial transcripts, at least 1000 UMIs, and that express at least 200 genes. Gene expression was log-normalised, and principal component analysis was performed on the top 2000 most variable genes. Individual Seurat objects were then integrated using Harmony (75) through IntegrateLayers, and the first 30 Harmony dimensions used as input for dimensionality reduction via UMAP. Subsequently, cells within the tdTomato- sort fraction that expressed tdTomato transcript and therefore represent likely sort contaminants were reannotated within the tdTomato+ group for downstream analysis. This clustering resulted in the identification of distinct populations mesothelial-derived (originating from *Msln*^*+*^ cells) and fibroblast-derived (originating from *Pdgfra*^*+*^*Pi16*^*+*^*Ly6c1*^*+*^*Dpp4*^*+*^) cells, of which fibroblast-derived cells were further subclustered for downstream analysis.

#### Analysis of publicly available scRNA-seq datasets

Single cell transcriptomes from healthy pancreas were obtained from processed data (1) (fibroXplorer.com) and integrated with fibroblast transcriptomes of untreated or PBS-injected mice, preserving original cluster identities. Pseudotime analysis was subsequently performed using the Monocle 3 package (76), defining Pi16+ fibroblasts as the starting node.

#### SCENIC analysis

Potential gene regulatory networks active in fibroblast subsets over the course of caerulein-induced acute pancreatitis were predicted using the SCENIC package (30). For each time point, 500 Pi16+ fibroblasts and 1500 Col15a1+ fibroblasts were downsampled from the original integrated object. The R implementation of SCENIC was run with default parameters, except for gene regulatory network inference, where GRNBoost was performed in Python due to its superior runtime. Due to the stochastic nature of the gene regulatory network inference step, slightly varying results are produced each run. In line with the developers’ suggestions, the SCENIC pipeline was run several times, and potential regulons were considered ‘high confidence’ if they occurred in >80% of runs. All regulons presented in figures were inferred in >80% of runs.

#### RNA velocity

Counting of spliced and unspliced RNA abundances in single cell transcriptomes was performed using velocyto (77). Subsequently, trajectory inference was performed with scVelo (78), using the generalised dynamical model for transcriptional dynamics. UMAP embeddings obtained from Seurat were imported for visualisation of cell trajectories.

#### Milo

Differential abundance analysis between tdTomato+ and tdTomato- groups within scRNAseq datasets was performed using the miloR (79) package, using parameters of k=20 and d=30 for buildGraph, prop=0.1, k=20, d=30, refined=T for makeNhoods, and d=30 for calcNhoodDistance. As tdTomato+ and tdTomato- cells can be matched by tumour of origin, testNhoods was performed with the argument design = ~ original_tumour + tdTom_grouping.

#### CellRank

Modelling of CAF population fates was performed using CellRank 2 (80). scVelo was used to set up the VelocityKernel, and a combined kernel created from velocity and connectivity kernels (0.8 * vk + 0.2 * ck). 12 macrostates were fitted using the GPCCA estimator, and a macrostate corresponding to the Dpp4^+^ fibroblast population set as the initial macrostate in accordance with the experimental ground truth. Subsequently, terminal macrostates were predicted, and per-1 cell fate probabilities computed.

### Statistics

Analysis for two groups were calculated using an unpaired two-tailed Student’s t-test; comparisons of more than two groups were calculated using a one-way analysis of variance (ANOVA) with Tukey post-analysis. All data were analysed using GraphPad Prism 9 (GraphPad Software). Results with p ≤ 0.05 were considered statistically significant (*), p ≤ 0.01 = **, p ≤ 0.001 = ***, p ≤ 0.0001 = ****.

## Supplementary Material

Reproducibility Checklist

Supp Data 1

Supp Data 2

Supp Figs 1-14

Supp Movie 1

## Data Availability

Transcriptomic data are available at GEO under accession numbers GSE248387 and GSE309073. Raw and processed transcriptomic data from (1) are available at ArrayExpress repository under the accession number E-MTAB-8483, and integrated scRNA-seq objects are available at https://fibroXplorer.com. All other data needed to evaluate the conclusions in the paper are present in the main text or the supplementary materials. No new code or material, other than those listed below, were generated. *Pdgfra*^*mGFP-CreERT2*^ mice are available from Prof. Acton via CancerTools.org under a material transfer agreement with University College London. *Dpp4*^*CreERT2*^ mice are available from Prof. Seale under a material agreement with the University of Pittsburgh. *Il33*^*Cit*^, *Il13*^*tom*^, *Lif*^*fl*^ and *Rora*^*fl*^ mice are available from Prof. McKenzie under a material transfer agreement with the MRC Laboratory of Molecular Biology. *Pi16*^*tm1(icre)Tcp*^ mice are available from Prof. Buechler at the Canadian Mouse Mutant Repository (cmmr.ca; strain AFFG) under a material transfer agreement with the University of Toronto.

## One-Sentence Summary

Pancreatic ILC2 inhabit a *Pi16*^*+*^*Dpp4*^*+*^*Ly6c*^*+*^ fibroblast niche and enforce homeostatic circuits that regulates both steady-state, inflammatory, and cancer associated fibroblast densities.

## References

[1] Buechler MB, Pradhan RN, Krishnamurty AT, Cox C, Calviello AK, Wang AW, Yang YA, Tam L, Caothien R, Roose-Girma M, Modrusan Z (2021). Cross-tissue organization of the fibroblast lineage. Nature.

[2] Davidson S, Coles M, Thomas T, Kollias G, Ludewig B, Turley S, Brenner M, Buckley CD (2021). Fibroblasts as immune regulators in infection, inflammation and cancer. Nat Rev Immunol.

[3] Monteran L, Erez N (2019). The Dark Side of Fibroblasts: Cancer-Associated Fibroblasts as Mediators of Immunosuppression in the Tumor Microenvironment. Front Immunol.

[4] Soliman H, Theret M, Scott W, Hill L, Underhill TM, Hinz B, Rossi FMV (2021). Multipotent stromal cells: One name, multiple identities. Cell Stem Cell.

[5] Meizlish ML, Franklin RA, Zhou X, Medzhitov R (2021). Tissue Homeostasis and Inflammation. Annu Rev Immunol.

[6] Wu D, Molofsky AB, Liang H-E, Ricardo-Gonzalez RR, Jouihan HA, Bando JK, Chawla A, Locksley RM (2011). Eosinophils sustain adipose alternatively activated macrophages associated with glucose homeostasis. Science.

[7] Feuerer M, Herrero L, Cipolletta D, Naaz A, Wong J, Nayer A, Lee J, Goldfine AB, Benoist C, Shoelson S, Mathis D (2009). Lean, but not obese, fat is enriched for a unique population of regulatory T cells that affect metabolic parameters. Nat Med.

[8] Gerbe F, Sidot E, Smyth DJ, Ohmoto M, Matsumoto I, Dardalhon V, Cesses P, Garnier L, Pouzolles M, Brulin B, Bruschi M (2016). Intestinal epithelial tuft cells initiate type 2 mucosal immunity to helminth parasites. Nature.

[9] von Moltke J, Ji M, Liang H-E, Locksley RM (2016). Tuft-cell-derived IL-25 regulates an intestinal ILC2-epithelial response circuit. Nature.

[10] Sanada S, Hakuno D, Higgins LJ, Schreiter ER, McKenzie ANJ, Lee RT (2007). IL-33 and ST2 comprise a critical biomechanically induced and cardioprotective signaling system. J Clin Invest.

[11] Mahlakõiv T, Flamar A-L, Johnston LK, Moriyama S, Putzel GG, Bryce PJ, Artis D (2019). Stromal cells maintain immune cell homeostasis in adipose tissue via production of interleukin-33. Sci Immunol.

[12] Rana BMJ, Jou E, Barlow JL, Rodriguez-Rodriguez N, Walker JA, Knox C, Jolin HE, Hardman CS, Sivasubramaniam M, Szeto A, Cohen ES (2019). A stromal cell niche sustains ILC2-mediated type-2 conditioning in adipose tissue. J Exp Med.

[13] Dahlgren MW, Jones SW, Cautivo KM, Dubinin A, Ortiz-Carpena JF, Farhat S, Yu KS, Lee K, Wang C, Molofsky AV, Tward AD (2019). Adventitial Stromal Cells Define Group 2 Innate Lymphoid Cell Tissue Niches. Immunity.

[14] Molofsky AB, Savage AK, Locksley RM (2015). Interleukin-33 in Tissue Homeostasis, Injury, and Inflammation. Immunity.

[15] Plikus MV, Wang X, Sinha S, Forte E, Thompson SM, Herzog EL, Driskell RR, Rosenthal N, Biernaskie J, Horsley V (2021). Fibroblasts: Origins, definitions, and functions in health and disease. Cell.

[16] Cayrol C, Girard J-P (2022). Interleukin-33 (IL-33): A critical review of its biology and the mechanisms involved in its release as a potent extracellular cytokine. Cytokine.

[17] Öhlund D, Handly-Santana A, Biffi G, Elyada E, Almeida AS, Ponz-Sarvise M, Corbo V, Oni TE, Hearn SA, Lee EJ, Chio IIC (2017). Distinct populations of inflammatory fibroblasts and myofibroblasts in pancreatic cancer. J Exp Med.

[18] Shakiba M, Tuveson DA (2025). Macrophages and fibroblasts as regulators of the immune response in pancreatic cancer. Nat Immunol.

[19] Dalmas E, Lehmann FM, Dror E, Wueest S, Thienel C, Borsigova M, Stawiski M, Traunecker E, Lucchini FC, Dapito DH, Kallert SM (2017). Interleukin-33-Activated Islet-Resident Innate Lymphoid Cells Promote Insulin Secretion through Myeloid Cell Retinoic Acid Production. Immunity.

[20] Dominguez CX, Müller S, Keerthivasan S, Koeppen H, Hung J, Gierke S, Breart B, Foreman O, Bainbridge TW, Castiglioni A, Senbabaoglu Y (2020). Single-cell RNA sequencing reveals stromal evolution into LRRC15+ myofibroblasts as a determinant of patient response to cancer immunotherapy. Cancer Discov.

[21] Chakarov S, Lim HY, Tan L, Lim SY, See P, Lum J, Zhang X-M, Foo S, Nakamizo S, Duan K, Kong WT (2019). Two distinct interstitial macrophage populations coexist across tissues in specific subtissular niches. Science.

[22] Apte MV, Pirola RC, Wilson JS (2012). Pancreatic stellate cells: a starring role in normal and diseased pancreas. Front Physiol.

[23] Nakandakari-Higa S, Walker S, Canesso MCC, van der Heide V, Chudnovskiy A, Kim D-Y, Jacobsen JT, Parsa R, Bilanovic J, Parigi SM, Fiedorczuk K (2024). Universal recording of immune cell interactions in vivo. Nature.

[24] Schluns KS, Kieper WC, Jameson SC, Lefrançois L (2000). Interleukin-7 mediates the homeostasis of naïve and memory CD8 T cells in vivo. Nat Immunol.

[25] Halim TYF, Hwang YY, Scanlon ST, Zaghouani H, Garbi N, Fallon PG, McKenzie ANJ (2016). Group 2 innate lymphoid cells license dendritic cells to potentiate memory TH2 cell responses. Nat Immunol.

[26] Molofsky AB, Nussbaum JC, Liang H-E, Van Dyken SJ, Cheng LE, Mohapatra A, Chawla A, Locksley RM (2013). Innate lymphoid type 2 cells sustain visceral adipose tissue eosinophils and alternatively activated macrophages. J Exp Med.

[27] Vassalli G (2019). Aldehyde Dehydrogenases: Not Just Markers, but Functional Regulators of Stem Cells. Stem Cells Int.

[28] De Martin A, Stanossek Y, Lütge M, Cadosch N, Onder L, Cheng H-W, Brandstadter JD, Maillard I, Stoeckli SJ, Pikor NB, Ludewig B (2023). PI16+ reticular cells in human palatine tonsils govern T cell activity in distinct subepithelial niches. Nat Immunol.

[29] Ansorge HL, Meng X, Zhang G, Veit G, Sun M, Klement JF, Beason DP, Soslowsky LJ, Koch M, Birk DE (2009). Type XIV Collagen Regulates Fibrillogenesis*. J Biol Chem.

[30] Aibar S, González-Blas CB, Moerman T, Huynh-Thu VA, Imrichova H, Hulselmans G, Rambow F, Marine J-C, Geurts P, Aerts J, van den Oord J (2017). SCENIC: single-cell regulatory network inference and clustering. Nat Methods.

[31] Zhang X, Yalcin S, Lee D-F, Yeh T-YJ, Lee S-M, Su J, Mungamuri SK, Rimmelé P, Kennedy M, Sellers R, Landthaler M (2011). FOXO1 is an essential regulator of pluripotency in human embryonic stem cells. Nat Cell Biol.

[32] Sue N, Jack BHA, Eaton SA, Pearson RCM, Funnell APW, Turner J, Czolij R, Denyer G, Bao S, Molero-Navajas JC, Perkins A (2008). Targeted disruption of the basic Krüppel-like factor gene (Klf3) reveals a role in adipogenesis. Mol Cell Biol.

[33] Gupta B, Errington AC, Jimenez-Pascual A, Eftychidis V, Brabletz S, Stemmler MP, Brabletz T, Petrik D, Siebzehnrubl FA (2021). The transcription factor ZEB1 regulates stem cell self-renewal and cell fate in the adult hippocampus. Cell Rep.

[34] Southard KM, Ardy RC, Tang A, O’Sullivan DD, Metzner E, Guruvayurappan K, Norman TM (2025). Comprehensive transcription factor perturbations recapitulate fibroblast transcriptional states. Nat Genet.

[35] Yan M, Komatsu N, Muro R, Huynh NC-N, Tomofuji Y, Okada Y, Suzuki HI, Takaba H, Kitazawa R, Kitazawa S, Pluemsakunthai W (2022). ETS1 governs pathological tissue-remodeling programs in disease-associated fibroblasts. Nat Immunol.

[36] Guan T, Fang F, Su X, Lin K, Gao Q (2020). Silencing PEG3 inhibits renal fibrosis in a rat model of diabetic nephropathy by suppressing the NF-κB pathway. Mol Cell Endocrinol.

[37] Stellato M, Dewenter M, Rudnik M, Hukara A, Özsoy Ç, Renoux F, Pachera E, Gantenbein F, Seebeck P, Uhtjaerv S, Osto E (2023). The AP-1 transcription factor Fosl-2 drives cardiac fibrosis and arrhythmias under immunofibrotic conditions. Communications Biology.

[38] Kishino A, Hayashi K, Hidai C, Masuda T, Nomura Y, Oshima T (2017). XBP1-FoxO1 interaction regulates ER stress-induced autophagy in auditory cells. Sci Rep.

[39] Capdevila J, Tsukui T, Rodríquez Esteban C, Zappavigna V, Izpisúa Belmonte JC (1999). Control of vertebrate limb outgrowth by the proximal factor Meis2 and distal antagonism of BMPs by Gremlin. Mol Cell.

[40] DeGregori J, Johnson DG (2006). Distinct and Overlapping Roles for E2F Family Members in Transcription, Proliferation and Apoptosis. Curr Mol Med.

[41] Sampieri L, Di Giusto P, Alvarez C (2019). CREB3 Transcription Factors: ER-Golgi Stress Transducers as Hubs for Cellular Homeostasis. Front Cell Dev Biol.

[42] Wang I-M, Lin H, Goldman SJ, Kobayashi M (2004). STAT-1 is activated by IL-4 and IL-13 in multiple cell types. Mol Immunol.

[43] Kang R, Lotze MT, Zeh HJ, Billiar TR, Tang D (2014). Cell death and DAMPs in acute pancreatitis. Mol Med.

[44] Mareninova OA, Sung K-F, Hong P, Lugea A, Pandol SJ, Gukovsky I, Gukovskaya AS (2006). Cell death in pancreatitis: caspases protect from necrotizing pancreatitis. J Biol Chem.

[45] Guo HY, Cui ZJ (2018). Extracellular Histones Activate Plasma Membrane Toll-Like Receptor 9 to Trigger Calcium Oscillations in Rat Pancreatic Acinar Tumor Cell AR4-2J. Cells.

[46] Hingorani SR, Wang L, Multani AS, Combs C, Deramaudt TB, Hruban RH, Rustgi AK, Chang S, Tuveson DA (2005). Trp53R172H and KrasG12D cooperate to promote chromosomal instability and widely metastatic pancreatic ductal adenocarcinoma in mice. Cancer Cell.

[47] Boothby IC, Kinet MJ, Boda DP, Kwan EY, Clancy S, Cohen JN, Habrylo I, Lowe MM, Pauli M, Yates AE, Chan JD (2021). Early-life inflammation primes a T helper 2 cell-fibroblast niche in skin. Nature.

[48] Li J, Byrne KT, Yan F, Yamazoe T, Chen Z, Baslan T, Richman LP, Lin JH, Sun YH, Rech AJ, Balli D (2018). Tumor Cell-Intrinsic Factors Underlie Heterogeneity of Immune Cell Infiltration and Response to Immunotherapy. Immunity.

[49] Krishnamurty AT, Shyer JA, Thai M, Gandham V, Buechler MB, Yang YA, Pradhan RN, Wang AW, Sanchez PL, Qu Y, Breart B (2022). LRRC15+ myofibroblasts dictate the stromal setpoint to suppress tumour immunity. Nature.

[50] Lange M, Bergen V, Klein M, Setty M, Reuter B, Bakhti M, Lickert H, Ansari M, Schniering J, Schiller HB, Theis FJ, Pe’er D (2022). CellRank for directed single-cell fate mapping. Nat Methods.

[51] Gong T, Liu L, Jiang W, Zhou R (2019). DAMP-sensing receptors in sterile inflammation and inflammatory diseases. Nat Rev Immunol.

[52] Navina S, Acharya C, DeLany JP, Orlichenko LS, Baty CJ, Shiva SS, Durgampudi C, Karlsson JM, Lee K, Bae KT, Furlan A (2011). Lipotoxicity causes multisystem organ failure and exacerbates acute pancreatitis in obesity. Sci Transl Med.

[53] Papachristou GI, Papachristou DJ, Avula H, Slivka A, Whitcomb DC (2006). Obesity increases the severity of acute pancreatitis: performance of APACHE-O score and correlation with the inflammatory response. Pancreatology.

[54] Šestan M, Raposo B, Rendas M, Brea D, Pirzgalska R, Rasteiro A, Aliseychik M, Godinho I, Ribeiro H, Carvalho T, Wueest S (2025). Neuronal-ILC2 interactions regulate pancreatic glucagon and glucose homeostasis. Science.

[55] Moral JA, Leung J, Rojas LA, Ruan J, Zhao J, Sethna Z, Ramnarain A, Gasmi B, Gururajan M, Redmond D, Askan G (2020). ILC2s amplify PD-1 blockade by activating tissue-specific cancer immunity. Nature.

[56] Alam A, Levanduski E, Denz P, Villavicencio HS, Bhatta M, Alhorebi L, Zhang Y, Gomez EC, Morreale B, Senchanthisai S, Li J (2022). Fungal mycobiome drives IL-33 secretion and type 2 immunity in pancreatic cancer. Cancer Cell.

[57] Stefkovich M, Traynor S, Cheng L, Merrick D, Seale P (2021). Dpp4+ interstitial progenitor cells contribute to basal and high fat diet-induced adipogenesis. Mol Metab.

[58] Horsnell HL, Tetley RJ, De Belly H, Makris S, Millward LJ, Benjamin AC, Heeringa LA, de Winde CM, Paluch EK, Mao Y, Acton SE (2022). Lymph node homeostasis and adaptation to immune challenge resolved by fibroblast network mechanics. Nat Immunol.

[59] Lee BY, Hogg EKJ, Below CR, Kononov A, Blanco-Gomez A, Heider F, Xu J, Hutton C, Zhang X, Scheidt T, Beattie K (2021). Heterocellular OSM-OSMR signalling reprograms fibroblasts to promote pancreatic cancer growth and metastasis. Nat Commun.

[60] Betz UA, Bloch W, van den Broek M, Yoshida K, Taga T, Kishimoto T, Addicks K, Rajewsky K, Müller W (1998). Postnatally induced inactivation of gp130 in mice results in neurological, cardiac, hematopoietic, immunological, hepatic, and pulmonary defects. J Exp Med.

[61] Nussbaum JC, Van Dyken SJ, von Moltke J, Cheng LE, Mohapatra A, Molofsky AB, Thornton EE, Krummel MF, Chawla A, Liang H-E, Locksley RM (2013). Type 2 innate lymphoid cells control eosinophil homeostasis. Nature.

[62] Tsou AM, Yano H, Parkhurst CN, Mahlakõiv T, Chu C, Zhang W, He Z, Jarick KJ, Zhong C, Putzel GG, Hatazaki M (2022). Neuropeptide regulation of non-redundant ILC2 responses at barrier surfaces. Nature.

[63] Kraman M, Bambrough PJ, Arnold JN, Roberts EW, Magiera L, Jones JO, Gopinathan A, Tuveson DA, Fearon DT (2010). Suppression of antitumor immunity by stromal cells expressing fibroblast activation protein-alpha. Science.

[64] Gogoi M, Clark PA, Ferreira ACF, Rodriguez Rodriguez N, Heycock M, Ko M, Murphy JE, Chen V, Luan S-L, Jolin HE, McKenzie ANJ (2024). ILC2-derived LIF licences progress from tissue to systemic immunity. Nature.

[65] Hardman CS, Panova V, McKenzie ANJ (2013). IL-33 citrine reporter mice reveal the temporal and spatial expression of IL-33 during allergic lung inflammation: Innate immunity. Eur J Immunol.

[66] Barlow JL, Bellosi A, Hardman CS, Drynan LF, Wong SH, Cruickshank JP, McKenzie ANJ (2012). Innate IL-13-producing nuocytes arise during allergic lung inflammation and contribute to airways hyperreactivity. J Allergy Clin Immunol.

[67] Schlenner SM, Madan V, Busch K, Tietz A, Läufle C, Costa C, Blum C, Fehling HJ, Rodewald H-R (2010). Fate mapping reveals separate origins of T cells and myeloid lineages in the thymus. Immunity.

[68] Oliphant CJ, Hwang YY, Walker JA, Salimi M, Wong SH, Brewer JM, Englezakis A, Barlow JL, Hams E, Scanlon ST, Ogg GS (2014). MHCII-mediated dialog between group 2 innate lymphoid cells and CD4(+) T cells potentiates type 2 immunity and promotes parasitic helminth expulsion. Immunity.

[69] Colucci F, Soudais C, Rosmaraki E, Vanes L, Tybulewicz VL, Di Santo JP (1999). Dissecting NK cell development using a novel alymphoid mouse model: investigating the role of the c-abl proto-oncogene in murine NK cell differentiation. J Immunol.

[70] Gertsenstein M, Nutter LMJ, Reid T, Pereira M, Stanford WL, Rossant J, Nagy A (2010). Efficient generation of germ line transmitting chimeras from C57BL/6N ES cells by aggregation with outbred host embryos. PLoS One.

[71] Nagy A, Gertsenstein M, Vintersten K, Behringer RR (2002). Manipulating the Mouse Embryo.

[72] Jin S, Guerrero-Juarez CF, Zhang L, Chang I, Ramos R, Kuan C-H, Myung P, Plikus MV, Nie Q (2021). Inference and analysis of cell-cell communication using CellChat. Nat Commun.

[73] Stuart T, Butler A, Hoffman P, Hafemeister C, Papalexi E, Mauck WM, Hao Y, Stoeckius M, Smibert P, Satija R (2019). Comprehensive integration of single-cell data. Cell.

[74] Germain P-L, Lun A, Garcia Meixide C, Macnair W, Robinson MD (2021). Doublet identification in single-cell sequencing data using scDblFinder. F1000Res.

[75] Korsunsky I, Millard N, Fan J, Slowikowski K, Zhang F, Wei K, Baglaenko Y, Brenner M, Loh P-R, Raychaudhuri S (2019). Fast, sensitive and accurate integration of single-cell data with Harmony. Nat Methods.

[76] Cao J, Spielmann M, Qiu X, Huang X, Ibrahim DM, Hill AJ, Zhang F, Mundlos S, Christiansen L, Steemers FJ, Trapnell C (2019). The single-cell transcriptional landscape of mammalian organogenesis. Nature.

[77] La Manno G, Soldatov R, Zeisel A, Braun E, Hochgerner H, Petukhov V, Lidschreiber K, Kastriti ME, Lönnerberg P, Furlan A, Fan J (2018). RNA velocity of single cells. Nature.

[78] Bergen V, Lange M, Peidli S, Wolf FA, Theis FJ (2020). Generalizing RNA velocity to transient cell states through dynamical modeling. Nat Biotechnol.

[79] Dann E, Henderson NC, Teichmann SA, Morgan MD, Marioni JC (2022). Differential abundance testing on single-cell data using k-nearest neighbor graphs. Nat Biotechnol.

[80] Weiler P, Lange M, Klein M, Pe’er D, Theis F (2024). CellRank 2: unified fate mapping in multiview single-cell data. Nat Methods.

