# Supplementary material for: ILC2 regulate a fibroblast progenitor niche in the pancreas
Source: Science. Author manuscript; Available in PMC 2026 Sep 8. (PMC7619465; doi:10.1126/science.aea5113)
Supplement: Supplementary file 2 [file EMS218158-supplement-Supp_Figs_1_14.pdf]

Science
AAAS

# Supplementary Materials for

## ILC2s regulate a fibroblast progenitor niche in the pancreas

Thomas Yip *et al*.

Corresponding authors: Matthew B. Buechler, matthew.buechler@utoronto.ca; Timotheus Y. F. Halim, tim.halim@cruk.cam.ac.uk

*Science* **393**, eaea5113 (2026)
DOI: 10.1126/science.aea5113

**The PDF file includes:**

Figs. S1 to S14
Tables S1 to S4

**Other Supplementary Material for this manuscript includes the following:**

Data S1 and S2
Movie S1
MDAR Reproducibility Checklist

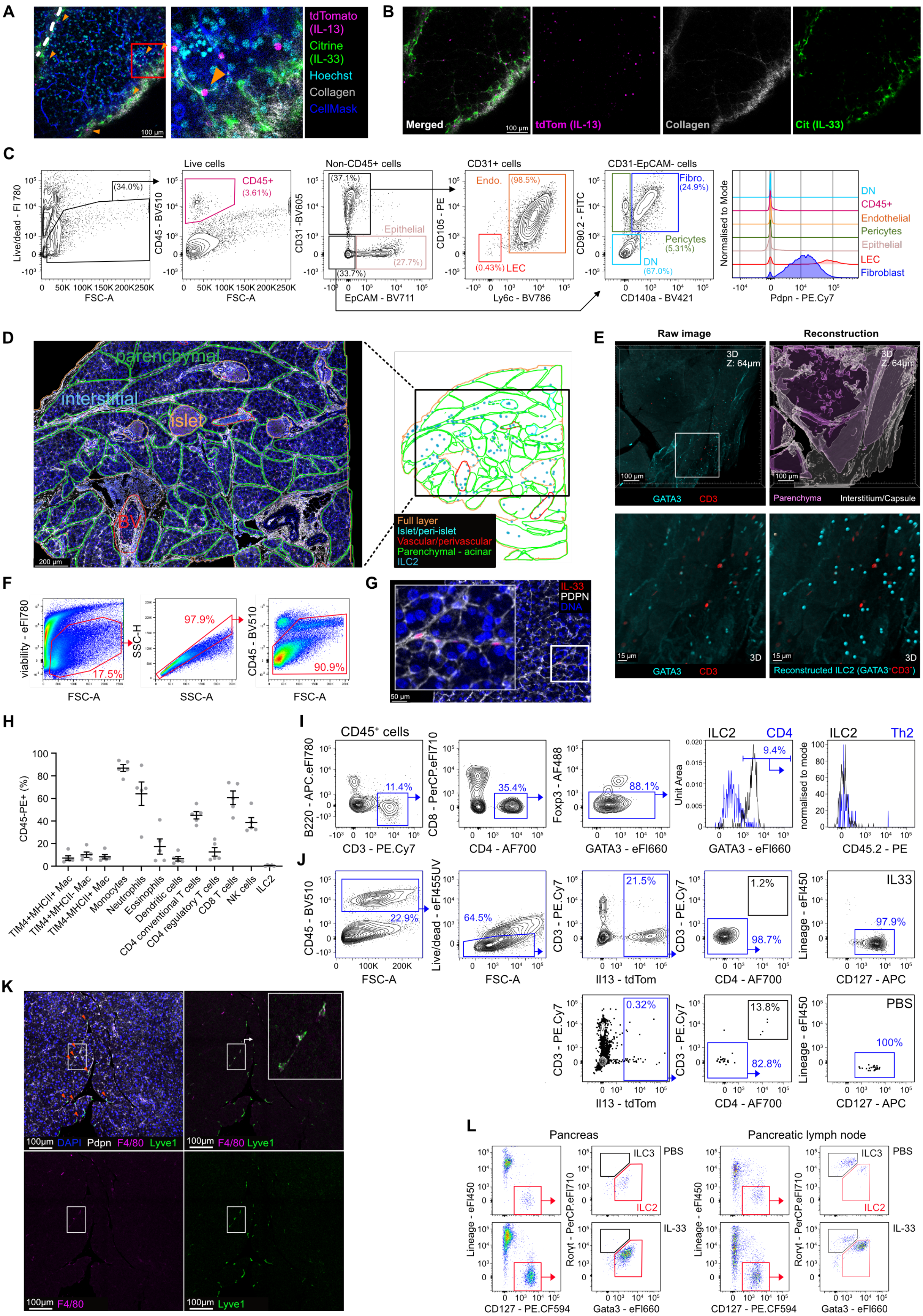

**Fig. S1. ILC2 co-localise with IL-33$^{+}$ fibroblast in an interstitial niche of the pancreas.**

(**A-B**) *Il13*$^{tdTom/+}$*Il33*$^{Cit/+}$ mice were treated with IL-33 on days -3, -2, and sacrificed on day 0. Mice were pre-administered with Hoechst (stains DNA) and CellMask (stains vasculature and parenchymal acinar cells) dyes immediately before sacrifice, and pancreas explants were imaged by 2-photon microscopy immediately after dissection. Dotted line indicates interstitial septae. Zoomed in image shows Il13-tdTom$^{+}$ (orange arrows) cells near Il33-Citrine$^{+}$ cells (**A**). The same image shown without CellMask and Hoechst as merged and individual channels (**B**). Scale bar: 100 μm.

(**C**) Expression of podoplanin (Pdpn) was measured on different cell-types in the pancreas of untreated C57Bl/6 mice.

(**D**) Formalin fixed paraffin embedded (FFPE) pancreata from C57Bl/6 mice, treated as in (A), were sectioned and analysed using multiplex immunofluorescence (IF) imaging. HALO image analysis was performed to identify ILC2 based on Gata3$^{+}$CD3$^{-}$ staining (dark blue, right), followed by measurement of their abundance in the indicated anatomical regions. Scale bar: 200 μm.

(**E**) Representative raw 3D image (left) and its reconstruction (right) of an optically cleared thick section from an untreated C57Bl/6 mouse pancreas stained for GATA3 and CD3. The reconstruction of the tissue and cells was done using the software Imaris. The parenchyma (purple) and interstitium (white) were automatically reconstructed with the 'surface' tool based on tissue autofluorescence. The bottom panel shows the magnification of the indicated ROI and the reconstruction of ILC2s (GATA3$^{+}$CD3$^{-}$) using the 'spots' tool. GATA3$^{+}$CD3$^{+}$ cells were not reconstructed. Scale bar: 100 μm (*top*), 15 μm (*bottom*). *(Same data as Fig. 1D, showing additional and more detailed staining for ILC2 in the capsular region of the pancreas.)*

(**F**) Pancreata were digested and enriched for exocrine cells by size exclusion of islets, followed by flow cytometry analysis of the single-cell suspension. Shown is the pre-gating strategy for live cell, single cells, and non-immune cells.

(**G**) Formalin fixed paraffin embedded (FFPE) pancreata were sectioned and stained for podoplanin (grey), nuclei (blue) and IL-33 (red). Scale bar: 50 μm.

(**H-I**) C57Bl/6 mice were administered intravenously with anti-CD45.2-PE mAb before sacrifice to label circulating immune cells. The indicated immune cells were identified in pancreatic digests by flow cytometry, and CD45.2-PE expression was used to determine the proportion of cells that were near blood vessels (**H**). Pancreatic Th2 cells were detected by CD45$^{+}$CD3$^{+}$CD4$^{+}$CD8$^{-}$Foxp3$^{-}$Gata3$^{+}$ phenotype, which, like Gata3$^{+}$ ILC2 (gated separately, shown in black as comparison), were assessed for CD45.2-PE (**I**). *(Same data as Fig. 1G, showing gating for pancreatic CD4$^{+}$ Th2 cells.)*

(**J**) *Il13*$^{tdTom/+}$ mice were treated with PBS or IL-33 on days -2, -1, and sacrificed on day 0. Pancreas was analysed by flow cytometry to characterise Il13$^{tdTom+}$ cells.

(**K**) C57Bl/6 mouse pancreas was stained for the indicated markers and imaged. Lyve1$^{+}$F4/80$^{+}$ cells (red arrow) were identified. Fibroblasts were identified by podoplanin staining. Scale bar: 100 μm.

(**L**) C57Bl/6 mice were treated with PBS or IL-33 on days -2 and -1 and sacrificed on day 0. Pancreas and pancreatic lymph node cells were analysed by flow cytometry for the presence of ILC2 and ILC3. Cells were pre-gated on Live, $CD45^{+}B220^{-}CD3^{-}NK1.1^{-}CD4^{-}CD8^{-}$ cells *(gating strategy in Fig. 1G)*. Lineage = CD5, CD19, CD11b, CD11c, F4/80, FcεRI, Ter-119, Ly6g.

Dot plots show representative gating strategy; numbers indicate the percent of gated cells. Bar graphs indicate mean (±SEM). Datapoints represent individual mice (**H**). **H**, n=5; data representative of 3 experiments. Imaging data is representative of 2 (**A**, **B**), 3 (**D**), 2 (**E**), and 3 (**K**) independent experiments.

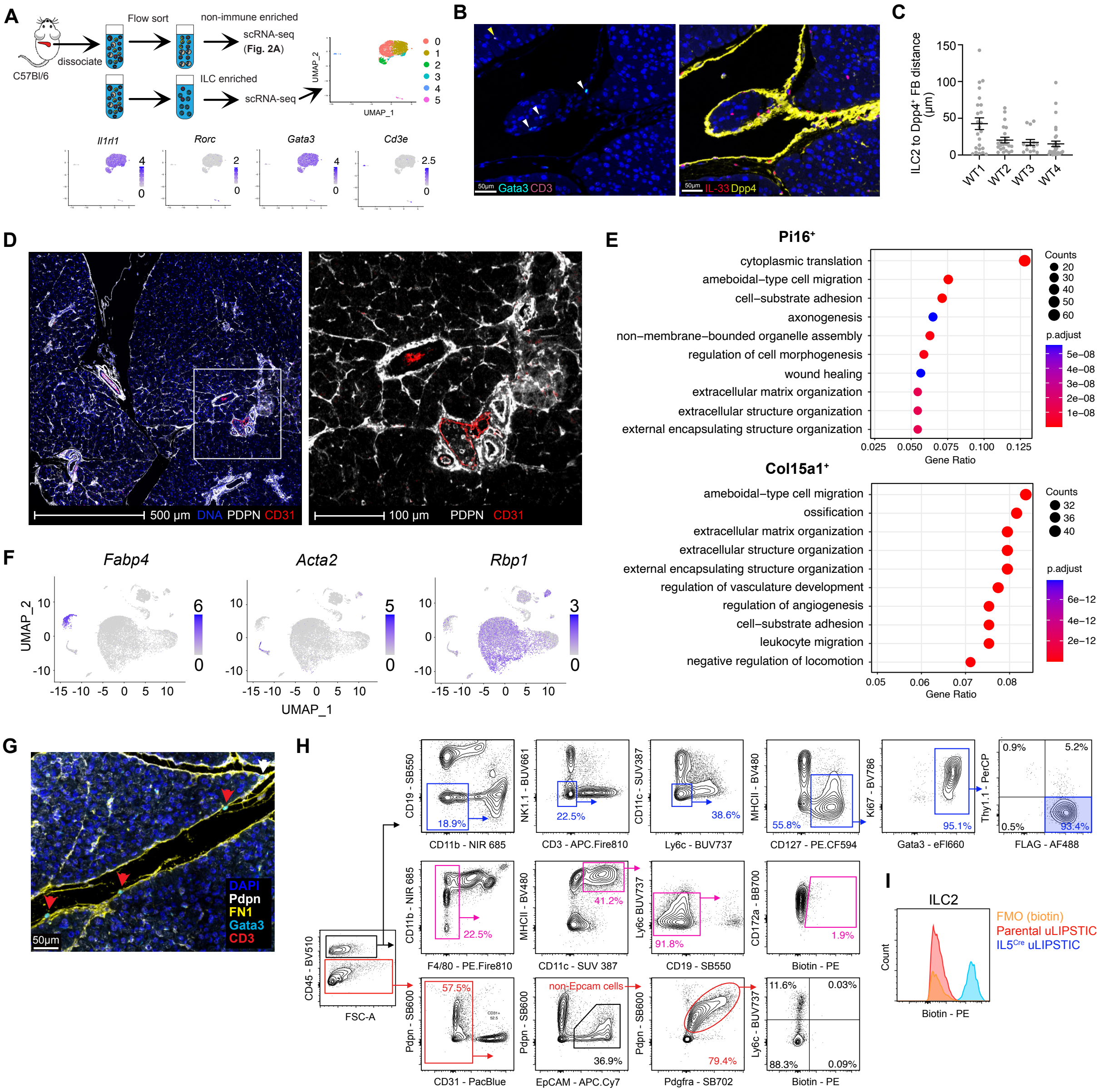


**Fig. S2. Topographical, transcriptomic, and proximity-labelling analysis of the pancreas uncover niche-specific interactions between ILC2 and fibroblast subsets.**

(**A**) Schematic for scRNA-seq workflow. Pancreata from C57Bl/6 mice were digested and enriched for exocrine cells by size exclusion of islets, followed by flow sorting of the single-cell suspension. Non-immune cells were gated on Live, CD45$^-$ cells. ILC-enriched cells were gated on Live, CD45$^+$B220$^-$CD3$^-$NK1.1$^-$CD127$^+$ cells. Single cell transcriptomic data of ILC-enriched cells from untreated C57Bl/6 mice were filtered for high quality events, log-normalised, and clustered into 6 cell clusters (*3368 cells*). Expression of marker genes for ILC2 (*Il1rl1, Gata3*), T cells (*Cd3e*), and ILC3 (*Rorc*) are shown.

(**B**) Formalin fixed paraffin embedded (FFPE) pancreas from untreated C57Bl/6 mice were sectioned and stained for the indicated markers (*related to Fig. 2E*). Arrows indicate examples of Gata3$^+$CD3$^-$ ILC2 (left panel), right panel shows DPP4 and IL-33 staining pattern. Scale bar: 50 µm. *(Same data as Fig. 2E, showing co-expression of DPP4 and IL-33).*

(**C**) Distances between Gata3$^{+}$CD3$^{-}$ (ILC2) and Dpp4$^{+}$Pdpn$^{+}$ fibroblast were calculated on sections of untreated C57Bl/6 mouse pancreas. Datapoint indicate individual cell distances, with 4 mice shown.
(**D**) Formalin fixed paraffin embedded (FFPE) pancreas from untreated C57Bl/6 mice were sectioned and stained for the indicated markers, showing CD31 and podoplanin staining pattern. Scale bar: 500 µm (*left*), 100 µm (*right*).
(**E**) Gene ontology enrichment analysis performed on the top 500 DEGs (ranked on adjusted p-value) for each cluster, on the GO Biological Process gene sets.
(**F**) Feature plot of the indicated genes of non-immune-enriched scRNA-seq dataset of untreated C57Bl/6 mice (*17358 cells from 3 mice*).
(**G**) Formalin fixed paraffin embedded (FFPE) pancreas from C57Bl/6 mice were sectioned and stained for the indicated markers. Scale bar: 50 µm.
(**H-I**) *Il5*$^{tdTom\text{-}iCre/+}$R26$^{\text{uLIPSTIC}}$ mice were treated with PBS or IL-33 on days -2 and -1, and pulsed with biotin-peptide substrate before sacrifice on day 0, followed by flow cytometry analysis of *in vivo* biotinylation of the indicated cell populations (**H**) via physical interaction with donor ILC2s (**I**).

Dot plots show representative gating strategy; numbers indicate the percent of gated cells. Bar graphs indicate mean (±SEM). Imaging data is representative of 4 (**B**), 2 (**D**), and 2 (**G**) independent experiments.

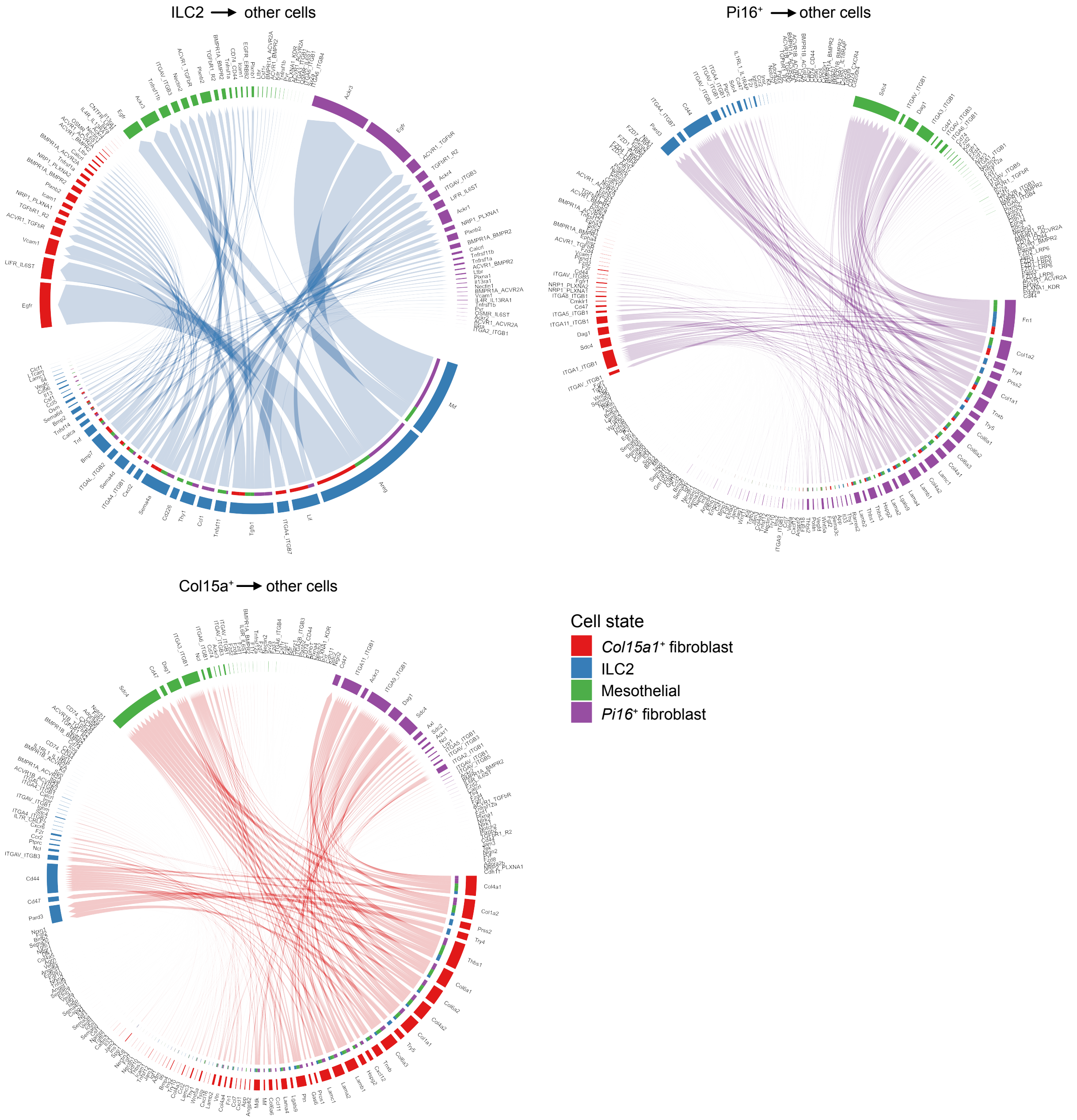


**Fig. S3 Transcriptomic analysis of the pancreas infers interactions between ILC2 and fibroblast subsets.**

CellChat analysis was performed on integrated dataset of ILC- and non-immune-enriched cells from untreated C57Bl/6 mice.

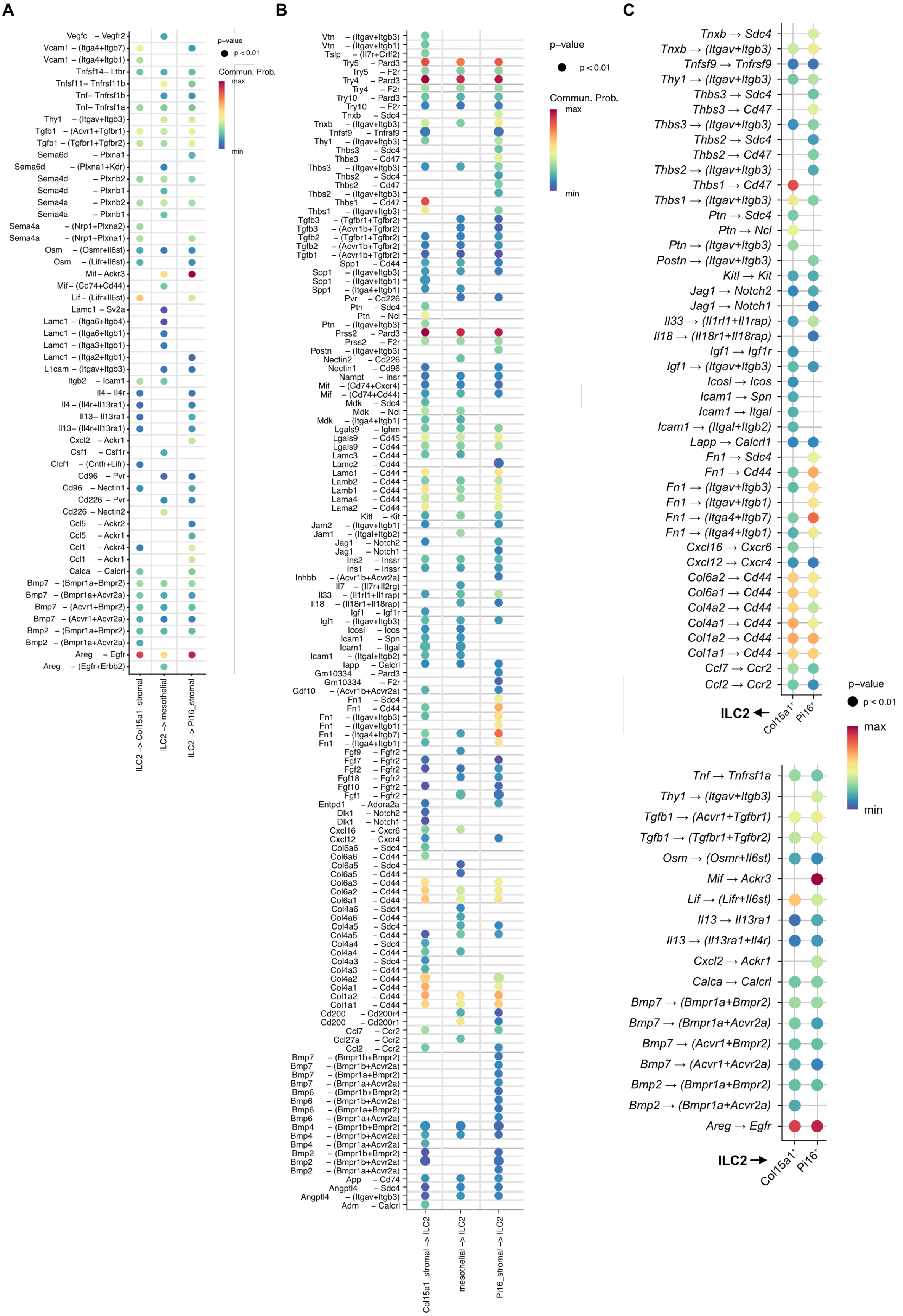


**Fig. S4. Transcriptomic analysis of the pancreas infers interactions between ILC2 and fibroblast subsets.** (**A-C**) CellChat analysis was performed on integrated dataset of ILC- and non-immune-

enriched cells from C57Bl/6 mice focussing on ILC2 to (**A**) and from (**B**) the indicated stromal populations. Selected pathways shown in (**C**).

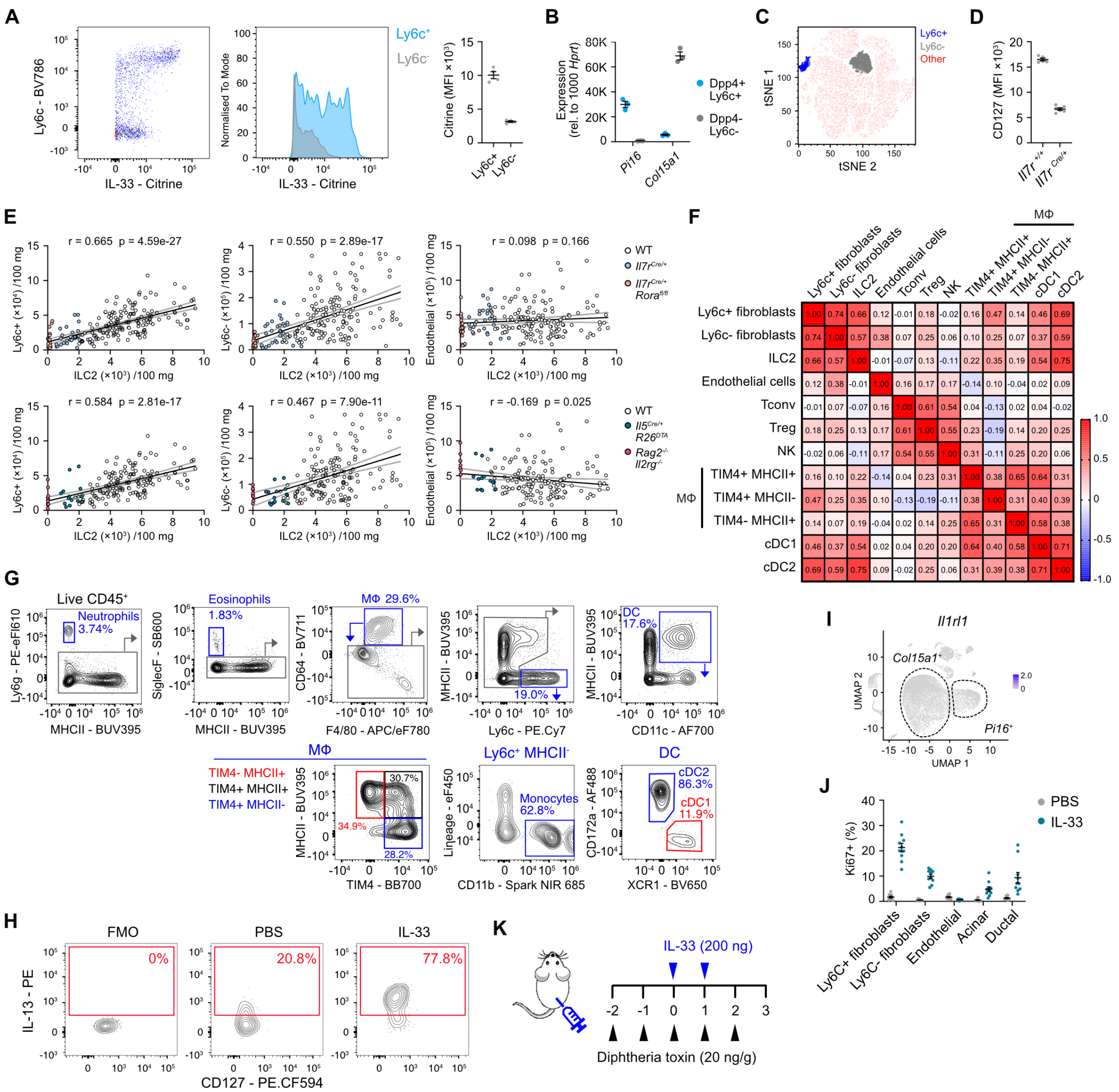


**Fig. S5. ILC2 influence steady-state fibroblast homeostasis and promote *in situ* expansion of putative Dpp4⁺Ly6c⁺ fibroblast progenitors.**

(**A**) Expression of Citrine was measured on pancreatic Ly6c$^+$ and Ly6c$^-$ fibroblasts in untreated *Il33*$^{Cit/+}$ mice *(gating strategy in Fig. 3A)*. Fibroblasts were identified as live CD45$^-$EpCAM$^-$CD31$^-$Pdpn$^+$CD140a$^+$ cells and analysed for Ly6c and Citrine (left dot plot), comparison of Ly6c$^+$ and Ly6c$^-$ fibroblasts (middle, representative histogram), or mean fluorescence intensity (MFI) of several mice.

(**B**) Pancreatic Ly6c$^+$Dpp4$^+$ and Ly6c$^-$Dpp4$^-$ fibroblasts were flow sorted from untreated C57Bl/6 mice, followed by qRT-PCR for the indicated genes.

(**C**) Unsupervised clustering was performed on flow cytometry data (non-immune panel) of untreated C57Bl/6 mice. Two fibroblast clusters were identified, which corresponded to the Ly6c$^+$ and Ly6c$^-$ subsets.

(**D**) Surface expression of CD127 was measured on live CD45$^+$CD3$^-$B220$^-$NK1.1$^-$Lineage$^-$Gata3$^+$ ILC2 in untreated mice of the indicated genotypes.

**(E-F)** Uninflamed pancreata from the indicated mouse genotypes were analysed by flow-cytometry to determine absolute numbers of ILC2, fibroblast subsets and endothelial cells (**E**), and correlations between absolute cell numbers of the indicated immune and non-immune cell populations (**F**).

(**G**) Gating strategy for flow cytometry identification of the indicated myeloid cell populations (neutrophils, eosinophils, TIM4$^{+}$MHCII$^{+}$ macrophages, TIM4$^{+}$MHCII$^{-}$ macrophages, TIM4$^{-}$MHCII$^{+}$ macrophages, monocytes, cDC1s, and cDC2s). Lineage = CD5, CD19, CD3e, NK1.1, B220.

(**H**) C57Bl/6 mice were injected with PBS or IL-33 on days 0 and 1, followed by analysis of intracellular IL-13 staining in pancreatic ILC by flow cytometry on day 2.

(**I**) Expression of *Il1rl1* in the pancreas non-immune dataset of untreated C57Bl/6 mice.

(**J**) C57Bl/6 mice were injected with PBS or IL-33 on days 0 and 1, followed by analysis of intracellular Ki67 staining in the indicated cell types by flow cytometry analysis on day 3.

(**K**) WT (Ctrl) and $Il5^{tdTom\text{-}iCre/tdTom\text{-}iCre}Rosa26^{lsl\text{-}DTR}$ (R5$^{DTR}$) mice were injected with PBS or Diphtheria toxin (DTX) plus IL-33, followed by flow-cytometry analysis of Ly6c$^{+}$ and Ly6c$^{-}$ fibroblasts for intracellular Ki67 expression on day 3 *(See Fig. 3H)*.

Dot plots show representative gating strategy; numbers indicate the percent of gated cells. Bar graphs indicate mean (±SEM). Datapoints represent individual mice (**A**, **B**, **D**, **E** and **J**). **A**, n=3; data representative of at least 3 independent experiments. **D**, n=5,6; data representative of at least 3 independent experiments; Two-tailed Student's t-test. **J**, n=10,10; data pooled from 2 independent experiments.

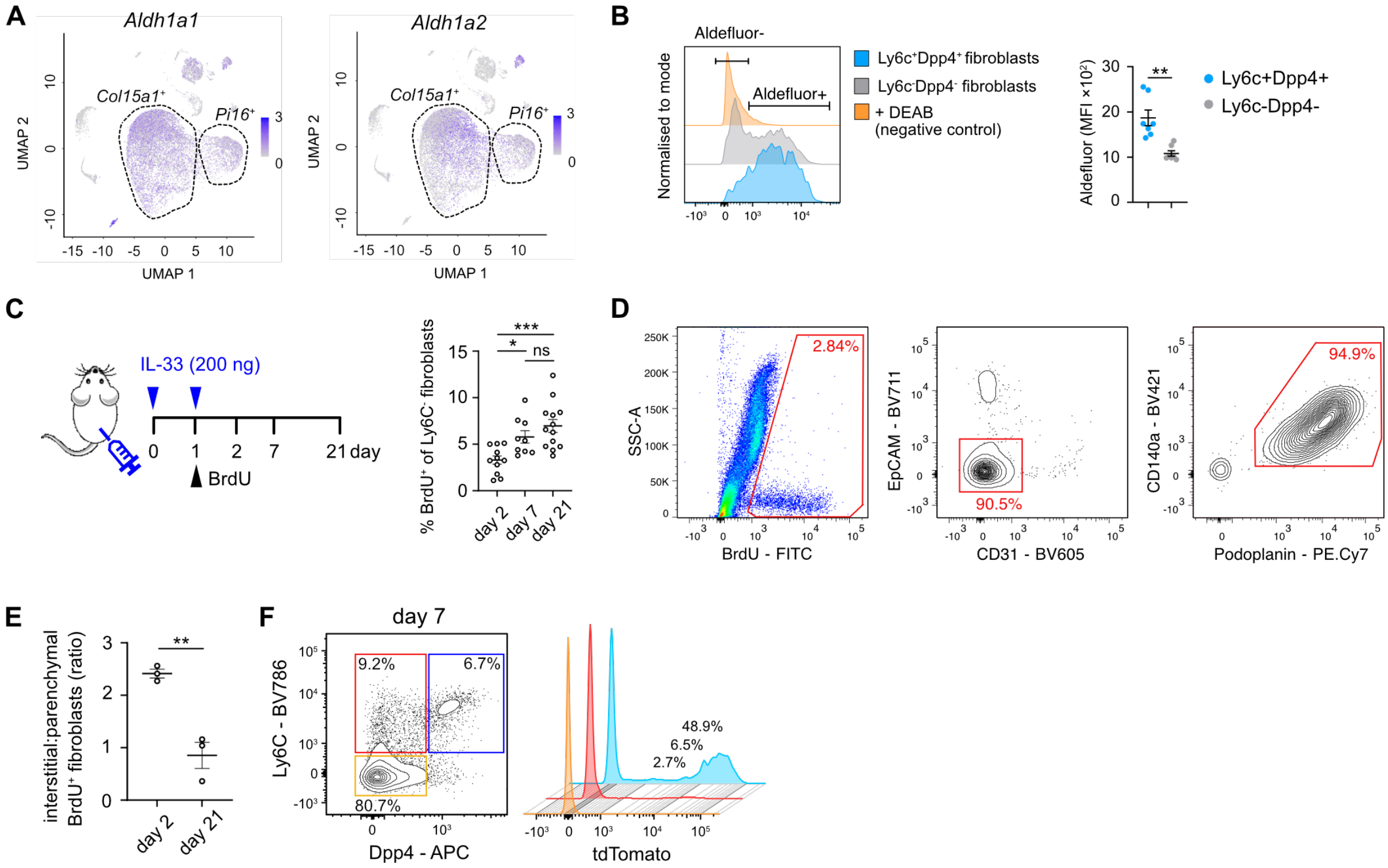


**Fig. S6 – ILC2-induced $Dpp4^{+}Ly6c^{+}$ fibroblast progenitors differentiate and repopulate the intra-parenchymal fibroblast compartment.**

(**A-B**) The expression of the indicated genes was measured in the pancreatic non-immune dataset at steady state (**A**) Fibroblasts were separated into $Ly6c^{+}Dpp4^{+}$ and $Ly6c^{-}Dpp4^{-}$ populations, and aldehyde dehydrogenase activity was measured using the Aldefluor assay by flow cytometry (**B**).

(**C-E**) C57Bl/6 mice were injected with IL-33 and BrdU followed by analysis at the indicated times (**C**, *left diagram*). Flow cytometry analysis on the pancreas was performed on day 2, 7 and 21 to determine the percentages of $BrdU^{+}$ cells amongst $Ly6c^{-}$ fibroblast (**C**, *right*). Non-immune $BrdU^{+}$ cells were primarily $EpCAM^{-}$ $CD31^{-}$ $Pdgfra^{+}$ $Pdpn^{+}$ cells (**D**, representative gating at day 2).

Whole pancreata was also collected and processed as FFPE tissue blocks from some animals for subsequent identification of $BrdU^{+}$ $PDPN^{+}$ fibroblasts in the indicated anatomical regions (**E**); the ratio of interstitial-to-parenchymal numbers was calculated at the indicated time points.

**(F)** *$Dpp4^{CreERT2/+}Rosa26^{lsl\text{-}tdTom}$* mice were treated with tamoxifen on days 0, 1, and 2 and sacrificed on day 7. Representative histogram showing the proportion of $tdTomato^{+}$ cells within the indicated pancreatic fibroblast subsets *(gating strategy in Fig. 3A)*.

Dot plots show representative gating strategy; numbers indicate the percent of gated cells. Bar graphs indicate mean (±SEM). **B**, n=7; data pooled from 2 independent experiments. Two-tailed Student's t-test. **C**, n=11,9,13; data pooled from 4 independent experiments. One-way ANOVA with Tukey's multiple comparisons test. **E**, n=3,3; data representative of 1 experiment. Two-tailed Student's t-test. ns = not significant, * = p ≤ 0.05, ** = p ≤ 0.01, *** = p ≤ 0.001.

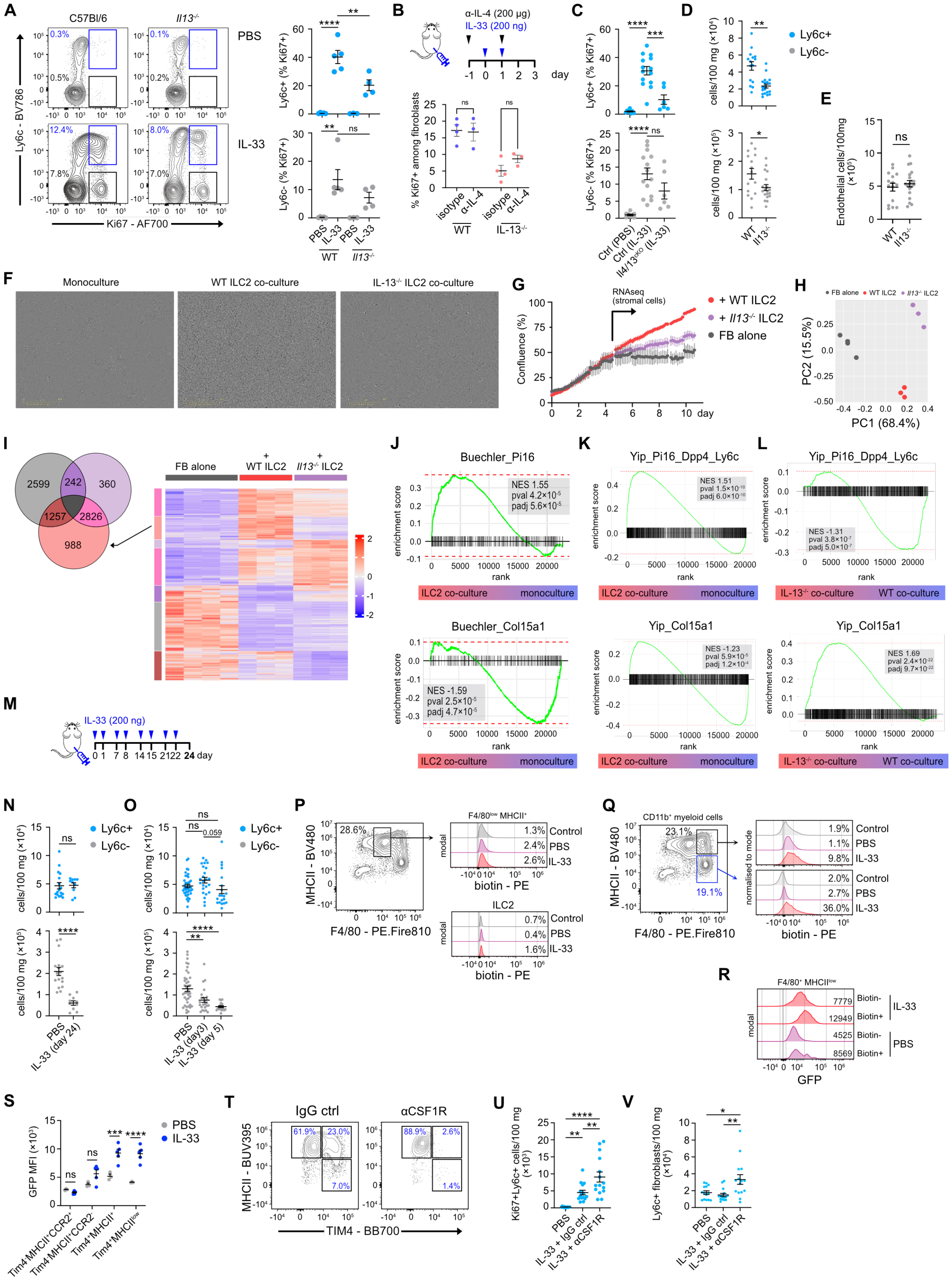

**Fig. S7. ILC2-dependent and independent mechanisms regulate *Pi16*⁺*Dpp4*⁺*Ly6c*⁺ fibroblast homeostasis.**

(**A**) C57Bl/6 or *Il13*$^{-/-}$ mice were injected with PBS or IL-33, followed by flow-cytometry analysis of Ly6c$^{+}$ and Ly6c$^{-}$ fibroblasts for intracellular Ki67 expression on day 3 (representative dot plot shown left, combined data for specific fibroblast subsets shown right).

(**B**) Mice of the indicated genotypes were injected with IL-33 plus anti-IL-4 or isotype control mAbs (top). Expression of Ki67 by fibroblasts was determined using flow cytometry analysis on day 3.

(**C**) Control or *Nmur*$^{iCre\text{-}eGFP}$*Il4/13*$^{fl/fl}$ mice (*Il4/13*$^{cKO}$) were injected with PBS or IL-33, followed by flow-cytometry analysis of Ly6c$^{+}$ and Ly6c$^{-}$ fibroblasts for intracellular Ki67 expression on day 3.

(**D-E**) Control or *Il13*$^{-/-}$ mice analysed for Ly6c$^{+}$ and Ly6c$^{-}$ fibroblasts (**D**) and endothelial cell (**E**) numbers at steady-state.

(**F**) Representative IncuCyte phase-contrast images of mono- or co-cultures of pancreatic fibroblasts plus ILC2 at the day 5 timepoint.

(**G-L**) Pancreatic fibroblasts flow-sorted from C57Bl/6 mice and subsequently cultured alone, or with pancreatic ILC2 from C57Bl/6 (WT) or *Il13*$^{-/-}$ mice. Fibroblast growth kinetics were measured using live imaging of culture confluence (**G**). In a separate experiment, fibroblasts were flow-sorted from cultures at the indicated time-point for bulk-RNA-seq analysis. Individual fibroblast transcriptome samples were visualised in a principal component analysis (PCA) plot, where colours indicate the culture conditions (**H**). Hierarchical clustering of differentially expressed genes (rows) in each condition (columns) revealed clusters of genes that are unique or shared between conditions (rows are colour coded and correspond with Venn diagram that lists numbers of genes in each cluster, *also see Table S4*) (**I**). Differentially expressed genes between C57Bl/6 ILC2 co-cultures and fibroblast monocultures were ranked and probed with signatures of *Pi16*$^{+}$ or *Col15a1*$^{+}$ fibroblast subsets using GSEA (**J-L**).

(**M-N**) C57Bl/6 mice were injected with PBS or rm-IL-33 on the indicated time points, followed by analysis and quantification of fibroblast subsets by flow cytometry analysis on day 24.

(**O**) C57Bl/6 mice were injected on day 0 and 1 with recombinant mouse IL-33 or PBS, followed by quantification of absolute numbers of pancreatic Ly6c$^{+}$ and Ly6c$^{-}$ fibroblasts at the indicated time-points.

(**P-R**) Tamoxifen-treated *Pdgfra*$^{mGFP\text{-}CreERT2/+}$Rosa26$^{\text{uLIPSTIC}}$ or Rosa26$^{\text{uLIPSTIC}}$ (Control) were treated with IL-33 or PBS on day 0 and 1, and pulsed with biotin-peptide substrate prior to flow analysis on day 2. Biotin expression on CD45$^{+}$CD11c$^{-}$Ly6c$^{-}$Ly6g$^{-}$ (pre-gated) CD11b$^{+}$F4/80$^{low}$MHCII$^{+}$ cells (**P**, *top right*), or CD45$^{+}$CD19$^{-}$CD11b$^{-}$CD3$^{-}$NK1.1$^{-}$CD127$^{+}$Gata3$^{+}$ ILC2 (**P**, *bottom right*). Biotin expression on CD45$^{+}$CD11c$^{-}$Ly6c$^{-}$Ly6g$^{-}$ (pre-gated) CD11b$^{+}$F4/80$^{+}$MHCII$^{high}$ macrophages (**Q,** *top right*) and CD11b$^{+}$F4/80$^{+}$MHCII$^{low}$ macrophages (**Q,** *bottom right***)**. GFP signal was measured for biotin-positive and biotin-negative CD45$^{+}$CD11c$^{-}$Ly6c$^{-}$Ly6g$^{-}$CD11b$^{+}$F4/80$^{+}$MHCII$^{low}$ macrophages from IL-33 and PBS treated mice (**R**).

(**S**) *Pdgfra*$^{mGFP\text{-}CreERT2/+}$ mice were treated with IL-33 or PBS on days 0 and 1, followed by analysis on day 2 of the indicated CD45$^{+}$CD11c$^{-}$Ly6c$^{-}$Ly6g$^{-}$CD11b$^{+}$F4/80$^{+}$ macrophage subsets *(gating strategy in Fig. S5G)* for GFP signal.

(**T-V**) C57Bl/6 mice were injected with anti-Csf1r or isotype control mAb on days -4, -2, and 1, and IL-33 on days 0 and 1, followed by analysis on day 3 for CD11b$^+$F4/80$^+$ macrophage subsets (**T**, *gating strategy in Fig. S5G*), Ki67$^+$Ly6c$^+$ fibroblasts (**U**), and Ly6c$^+$ fibroblasts (**V**).

Dot plots show representative gating strategy; numbers indicate the percent of gated cells. Bar graphs indicate mean (±SEM). **A**, n=5,5,4,4; representative data of 2 independent experiments. Two-way ANOVA with Šidák's multiple comparisons test. **B**, n=4,3,4,3; data representative of 1 experiment. Two-way ANOVA with Šidák's multiple comparisons test. **C**, n=8,14,6; data pooled from 2 independent experiments. One-way ANOVA with Tukey's multiple comparisons test. **D**, **E**, n=15,19; data pooled from 3 independent experiments. Two-tailed Student's t-test. **N**, n=17,10; data pooled from 2 independent experiments. Two-tailed Student's t-test. **O**, n=40,24,20; data pooled from 9 independent experiments. One-way ANOVA with Tukey's multiple comparisons test. **S**, n=3,5; data representative of 2 independent experiments. Two-way ANOVA with Šidák's multiple comparison test. **U**, **V**, n=14,15,15; data pooled from 3 independent experiments. One-way ANOVA with Tukey's multiple comparisons test. ns = not significant, * = $p \leq 0.05$, ** = $p \leq 0.01$, *** = $p \leq 0.001$, **** = $p \leq 0.0001$.

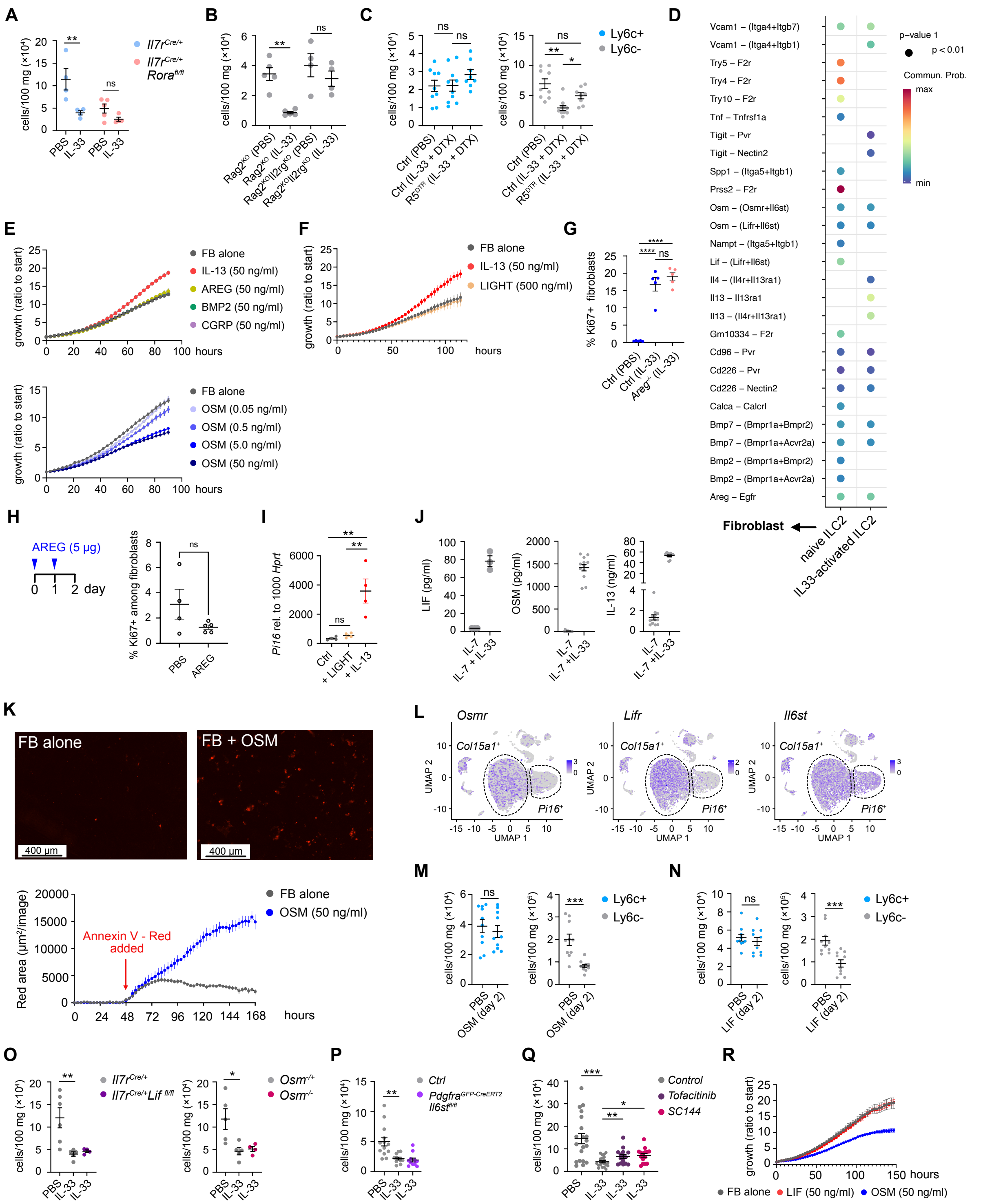


**Fig. S8. IL-33 activated ILC2 indirectly constrain *Col15a1*$^{+}$*Eng*$^{+}$*Ly6c*$^{-}$ fibroblast numbers.**

(**A**) Mice of the indicated genotypes were injected with PBS or IL-33 on days 0 and 1, followed by quantification of the absolute number of pancreatic Ly6c$^{-}$ fibroblasts on day 3.

(**B**) *Rag2*$^{-/-}$, or *Rag2*$^{-/-}$*Il2rg*$^{-/-}$ mice were injected with PBS or rm-IL-33 *(as indicated in Fig. S7M)*, followed by analysis of abundance of Ly6c$^{-}$ fibroblasts by flow cytometry analysis on day 24.

(**C**) *Il5*$^{tdTom\text{-}iCre/tdTom\text{-}iCre}$*Rosa26*$^{lsl\text{-}DTR}$ (R5$^{DTR}$) or control mice were treated with PBS or IL-33 plus Diphtheria toxin (*see Fig. S5K),* followed by quantification of absolute numbers of pancreatic Ly6c$^{+}$ and Ly6c$^{-}$ fibroblasts on day 3.

(**D**) Fibroblast bulk transcriptomic data from *in vitro* monocultures and pancreatic ILC2 bulk transcriptomic datasets from PBS-treated or IL-33-treated mice were used to infer two-way communication mechanisms using CellChat.

**(E-F)** Pancreatic fibroblasts were cultured with the indicated molecules, and growth was monitored over time using live imaging; individual cultures were normalised to their starting confluence.

(**G**) WT or *Areg*$^{-/-}$ mice were injected with PBS or rm-IL-33 on days 0 and 1, followed by analysis of intracellular Ki67 staining among fibroblasts by flow cytometry analysis on day 3.

(**H**) C57Bl/6 mice were injected with PBS or rmAREG on days 0 and 1, followed by analysis of intracellular Ki67 staining among fibroblasts by flow cytometry analysis on day 2.

(**I**) Pancreatic fibroblasts were cultured with rm-IL-13 (50 ng/ml) or rm-LIGHT (500 ng/ml) *(related to Fig. S8F)*, expression of the indicated genes analysed by RNA extraction and qPCR.

(**J**) Pancreatic ILC2 were flow-sorted from mice injected with IL-33 for *in vivo* expansion of ILC2 and subsequently cultured *in vitro* for 5 days with IL-7 or IL-7 plus IL-33. Supernatants were analysed for LIF, OSM, and IL-13 concentration by ELISA.

(**K**) Pancreatic fibroblasts were cultured with rm-OSM (50 ng/ml) for 48 hours. Media was washed and replaced with fresh media containing OSM and Incucyte Annexin V-Red dye for visualization and quantification of apoptotic cell death.

(**L**) Oncostatin M receptor (*Osmr*), Lif receptor (*Lifr*) and gp130 (*Il6st*) gene expression as a feature-plot of untreated pancreas; fibroblast sub-clusters are identified by dotted line.

(**M-N**) C57Bl/6 mice were injected on day 0 and 1 with recombinant mouse OSM (**M**) or LIF (**N**) followed by quantification of absolute numbers of pancreatic Ly6c$^{+}$ and Ly6c$^{-}$ fibroblasts on day 2.

(**O-P**) Mice of the indicated genotypes were administered PBS or rm-IL-33 on days 0 and 1, followed by quantification of absolute numbers of pancreatic Ly6c$^{-}$ fibroblasts on day 5. In (P) mice were also treated with tamoxifen on days -14 to -12.

(**Q**) C57Bl/6 mice were administered PBS or rm-IL-33 on days 0 and 1, alongside daily administration of the indicated inhibitors or vehicle control from days 0 to 4, followed by quantification of absolute numbers of pancreatic Ly6c$^{-}$ fibroblasts on day 5.

(**R**) Pancreatic fibroblasts were cultured with rm-LIF or rm-OSM (50 ng/ml), and growth was monitored over time using live imaging; individual cultures were normalised to their starting confluence.

Bar graphs indicate mean (±SEM). **A**, n=4,4,5,4; data representative of 2 independent experiments. Two-way ANOVA with Šidák's multiple comparison test. **B**, n=5,5,4,4; data from 1 experiment. One-way ANOVA with Tukey's multiple comparisons test. **C**, n=9,10,8; data pooled from 2 independent experiments. One-way Brown-Forsythe and Welch ANOVA with Dunnett's T3 multiple comparisons

test. **G**, n=5,5,5; data from 1 experiment. One-way ANOVA with Tukey's multiple comparisons test. **H**, n=4,5; data from 1 experiment. Two-tailed Student's t-test. **I**, n=4,4,4; data from 1 experiment. One-way ANOVA with Tukey's multiple comparisons test. **J**, n=3,3 (LIF); data from 1 experiment. n=12,12 (OSM and IL-13); pooled from 3 independent experiments. **M**, n=10,10; data pooled from 2 independent experiments. Two-tailed Student's t-test. **N**, n=10,10; data pooled from 2 experiments. Two-tailed Student's t-test. **O**, n=6,5,4 (left); data from 1 experiment. One-way ANOVA with Tukey's multiple comparisons test. n=5,5,4 (right); data from 1 experiment. One-way ANOVA with Tukey's multiple comparisons test. **P**, n=13,11,11; data pooled from 2 independent experiments. One-way ANOVA with Tukey's multiple comparisons test. **Q**, n=20,20,15,15; data pooled from 4 independent experiments. Brown-Forsythe and Welch ANOVA test. ns = not significant, * = $p \leq 0.05$, ** = $p \leq 0.01$, *** = $p \leq 0.001$, **** = $p \leq 0.0001$.

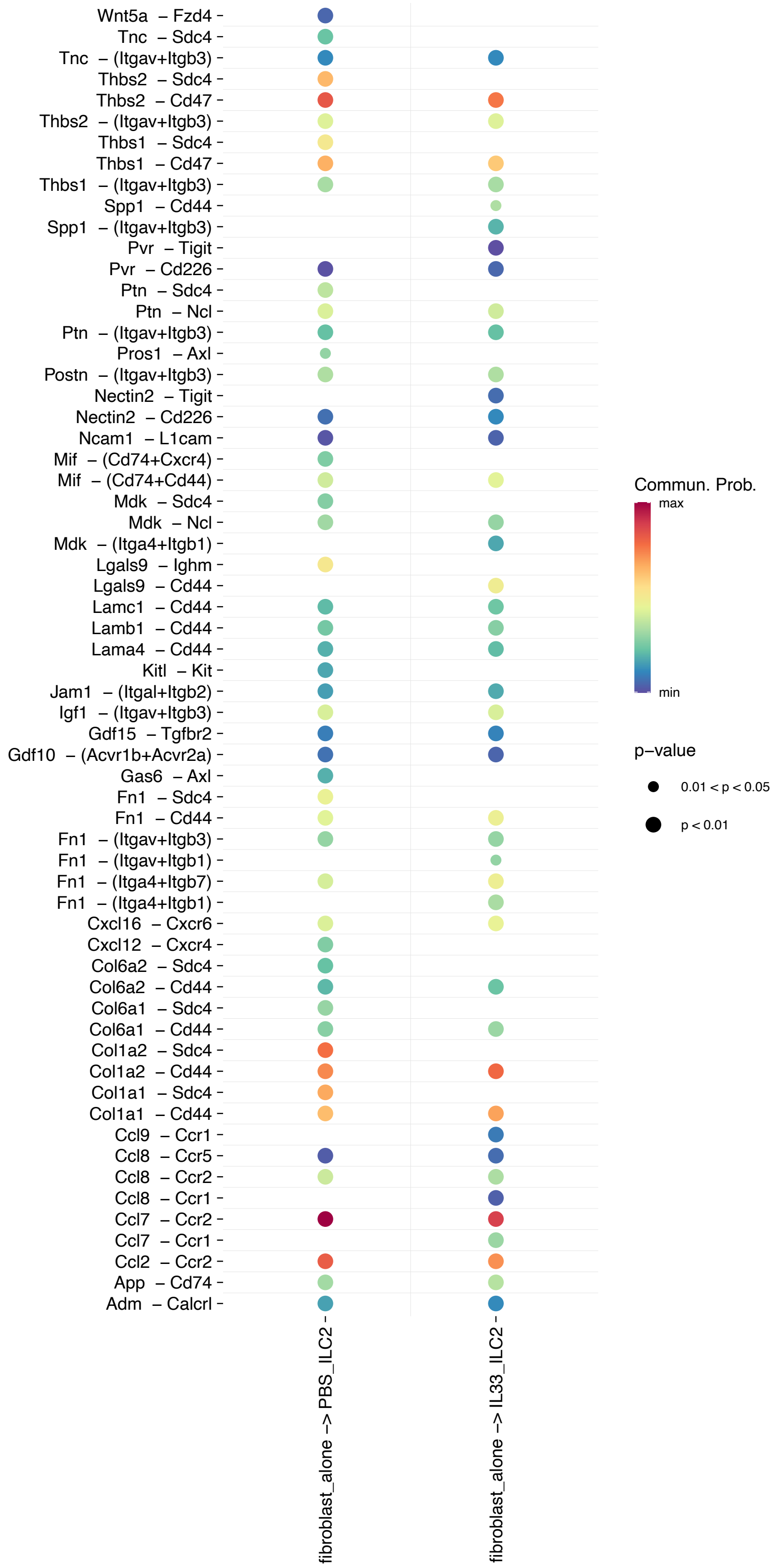


**Fig. S9. CellChat analysis on ex vivo ILC2 – fibroblast cultures.**

Fibroblast bulk transcriptomic data from *in vitro* monocultures and pancreatic ILC2 bulk transcriptomic datasets from PBS-treated or IL-33-treated mice were used to infer two-way communication mechanisms using CellChat.

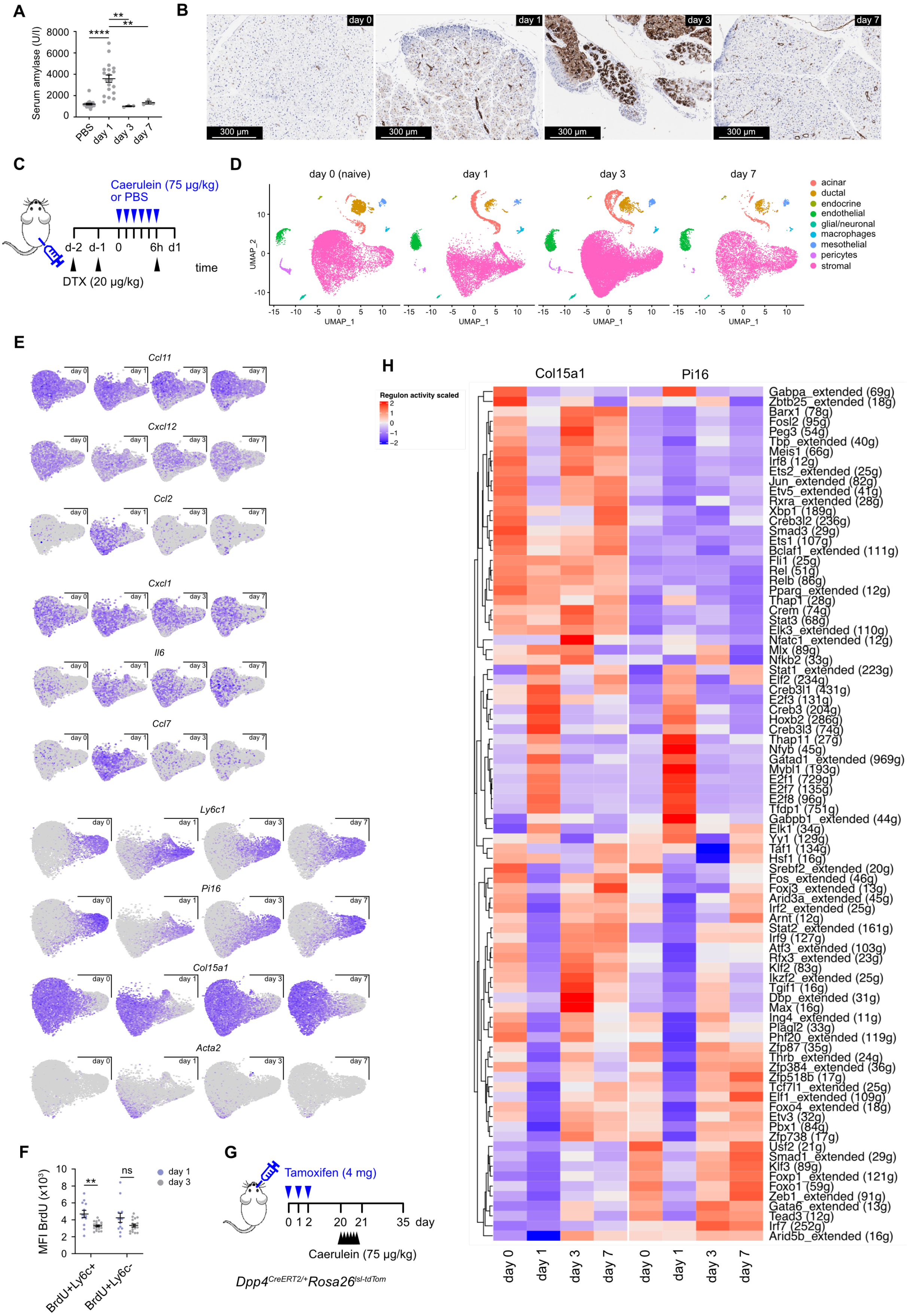

**Fig. S10. ILC2 promote the expansion of fibroblast progenitors in acute pancreatitis.**

(**A-B**) Mice were injected intraperitoneally with caerulein or PBS as indicated in *Fig. 5A*, followed by sacrifice on day 1, 3 or 7. Serum amylase activity was measured in PBS (control) or day 1, 3, and 7 caerulein-treated groups (**A**). Pancreata from untreated (day 0) or caerulein-treated (day 1, 3, and 7) were fixed and stained for Cytokeratin 19 by IHC as a measure of acinar-to-ductal metaplasia (**B**).

(**C**) $Il5^{tdTom\text{-}icre/tdTom\text{-}iCre}Rosa26^{lsl\text{-}DTR}$ or $Il5^{tdTom\text{-}icre/tdTom\text{-}iCre}$ mice were administered caerulein and diphtheria toxin (DTX) at the indicated time points for flow cytometry analysis of proliferating fibroblasts.

(**D-E**) Non-immune cells from untreated or caerulein treated C57Bl/6 mice were isolated using flow sorting at the indicated timepoints, followed by 10X scRNA-seq analysis; unsupervised clustering was performed on the integrated dataset, followed by identification of the indicated cell types (shown at the indicated timepoints, **D**; *17358, 9288, 24976, 6840* cells respectively). The fibroblast cluster was subsequently assessed for expression of the indicated genes at individual timepoints (**E**).

(**F**) Mice were treated with caerulein, and BrdU then administered 12 hours after pancreatitis induction. BrdU incorporation was assayed by flow cytometry. Mean fluorescence intensity (MFI) of BrdU was measured for $Ly6c^+$ and $Ly6c^-$ fibroblast subsets for each time point.

(**G**) $Dpp4^{CreERT2/+}Rosa26^{lsl\text{-}tdTom}$ were administered tamoxifen on days 0, 1, and 2, and acute pancreatitis induced on day 20 by repeated hourly administration of caerulein. Mice were sacrificed on day 35.

(**H**) $Pi16^+$ and $Col15a1^+$ fibroblast clusters were analysed for regulon activity at the indicated times using SCENIC.

Bar graphs indicate mean (±SEM). **A**, n=20,19,3,3; data pooled from 6 independent experiments. One-way ANOVA with Tukey's multiple comparisons test. **F**, n=17,18; data pooled from 3 independent experiments. Two-way ANOVA with Šidák's multiple comparisons test. ns = not significant, ** = $p \leq 0.01$, **** = $p \leq 0.0001$.

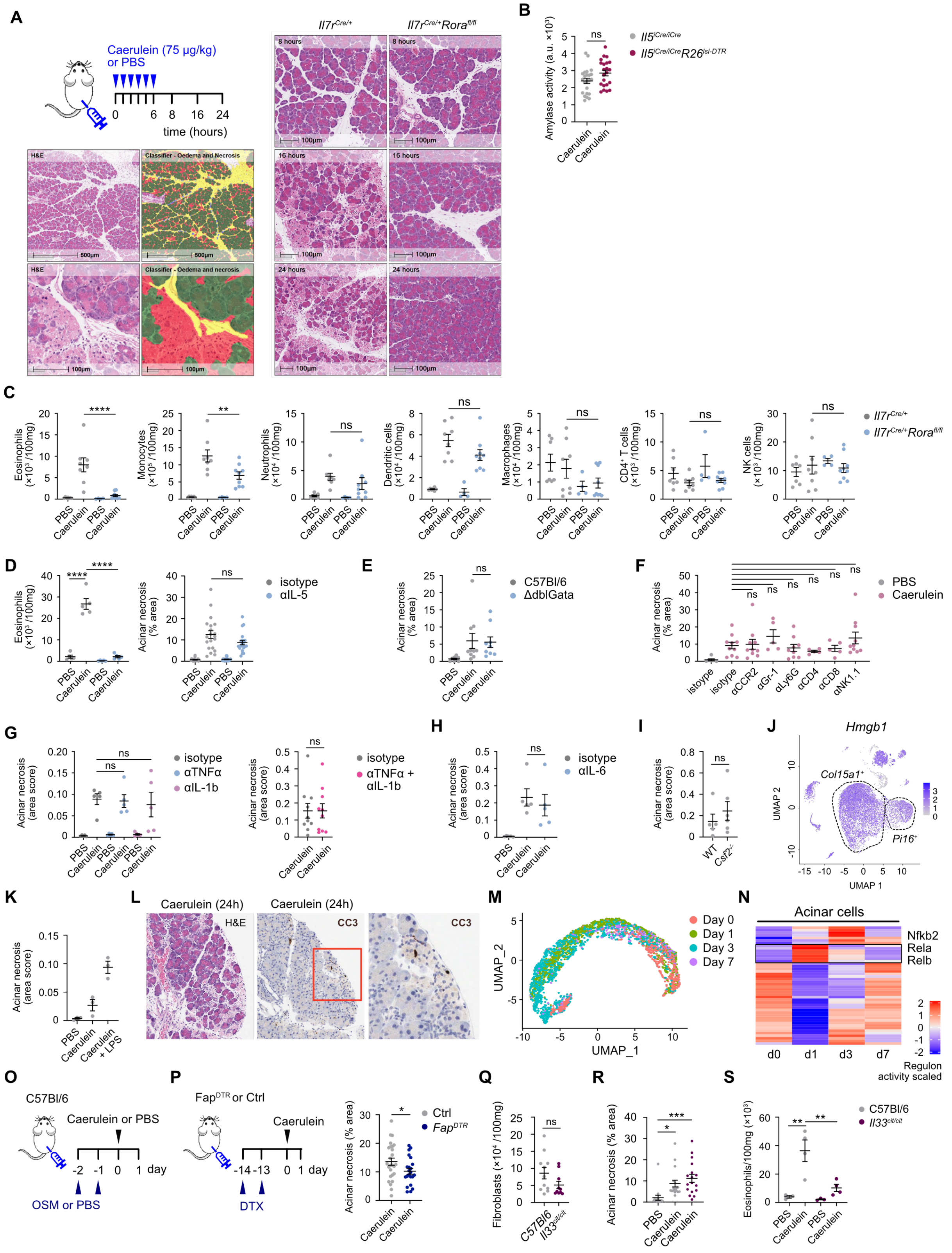

**Fig. S11. Steady-state fibroblast density set an inflammatory threshold for acute injury.**

(**A**) Mice were injected intraperitoneally with caerulein or PBS as indicated, followed by sacrifice at 8-, 16- or 24-hour timepoints (*top left*). Pancreata were formalin fixed and paraffin embedded, followed by H&E staining; representative images of caerulein treated *Il7r*$^{Cre/+}$ (control) and *Il7r*$^{Cre/+}$*Rora*$^{fl/fl}$ (ILC2-deficient) mice at the indicated timepoints (*right*). Disease severity was measured by automated image

analysis of whole tissue sections using HALO software (*bottom left*); areas of healthy tissue (green), acinar necrosis (red) or tissue edema (yellow) were determined and quantified, resulting in measures of percent acinar necrosis (or acinar necrosis area score). Two representative examples are shown. Scale bar: 500 µm (*left, top*) or 100 µm (*left, bottom and right panels)*.

(**B**) $Il5^{tdTom\text{-}icre/tdTom\text{-}iCre}Rosa26^{lsl\text{-}DTR}$ or $Il5^{tdTom\text{-}icre/tdTom\text{-}iCre}$ mice were administered caerulein and diphtheria toxin (*See Fig. S10C*) for measurement of serum amylase activity at 24 hours post-injury.

(**C**) Mice of the indicated genotypes were treated with PBS or caerulein as in (A) and sacrificed at 24 hours, followed by quantification of the indicated immune cells using flow cytometry.

(**D**) C57Bl/6 mice were treated with PBS or caerulein as in (A) while receiving anti-IL-5 or isotype control mAb (day -9, -4 and -1); mice were sacrificed at 24 hours, followed by quantification of pancreatic eosinophils by flow cytometry (*left*), or histological analysis for acinar cell necrosis (*right*).

(**E**) Mice of the indicated genotypes were treated with PBS or caerulein as in (A) and sacrificed at 24 hours, followed by histological analysis for acinar cell necrosis.

(**F**) C57Bl/6 mice were treated with caerulein as in (A) while receiving the indicated depleting or neutralising antibody or isotype control mAb (day -1, 0); mice were sacrificed at 24 hours, histological analysis for acinar cell necrosis.

(**G**) C57Bl/6 mice were treated with PBS or caerulein as in (A) while receiving the indicated neutralizing antibody alone (*left*), in combination (*right*), or isotype control mAb (day -1, 0); mice were sacrificed at 24 hours, histological analysis for acinar cell necrosis.

(**H**) C57Bl/6 mice were treated with caerulein as in (A) while receiving anti-IL-6 or isotype control mAb (day -1, 0); mice were sacrificed at 24 hours, histological analysis for acinar cell necrosis.

(**I**) Mice of the indicated genotypes were treated with caerulein as in (A) and sacrificed at 24 hours, followed by histological analysis for acinar cell necrosis.

(**J**) The expression of *Hmgb1* was measured in the pancreatic non-immune dataset at steady state.

(**K**) C57Bl/6 mice were treated with caerulein as in (A) +/- LPS (10 mg/kg); mice were sacrificed at 24 hours, histological analysis for acinar cell necrosis.

(**L**) C57Bl/6 mice were treated with caerulein; mice were sacrificed at 24 hours followed by staining of serial sections with H&E or anti-cleaved caspase 3 (CC3) IHC.

(**M-N**) Acinar cells were identified in our acute pancreatitis sc-RNA-seq time-course experiment and reclustered (**M**, *2988 cells*) and subsequently analysed for regulon activity at the indicated times (**N**).

(**O-P**) Fibroblasts were acutely depleted prior to acute pancreatitis induction via administration of recombinant OSM (**O**), or via DT administration to $Fap^{DTR}$ mice or littermate control, and histological assessment of pancreatic damage as indicated by acinar cell necrosis (**P**).

(**Q-S**) C57Bl/6 and $Il33^{cit/cit}$ mice were analysed for steady state fibroblast numbers in the pancreas (**Q**) or were treated with PBS or caerulein as in (A) and sacrificed at 24 hours, followed by histological analysis for acinar cell necrosis (**R**), or quantification of pancreatic eosinophils using flow cytometry analysis (**S**).

Bar graphs indicate mean (±SEM). **B**, n=23,22; data pooled from 3 independent experiments. Two-tailed Student's t-test. **C**, n=7,8,4,9; data pooled from 2 independent experiments. One-way ANOVA with Tukey's multiple comparisons test. **D**, n=5,5,5,5 (left) from 1 experiment. n=20,20,20,20 (right); data pooled from 4 independent experiments. One-way ANOVA with Tukey's multiple comparisons test. **E**, n=8,10,9; data pooled from 2 independent experiments. One-way ANOVA with Tukey's multiple comparisons test. **F**, n=6,10,9,5,5,5,10,9; data pooled from 2 independent experiments (untreated, isotype, αNK1.1, αCCR2 and αLy6G) or one experiment (αGR1, αCD4 and αCD8). **G**, n=5,5,5,5,5,5 (left); data from one experiment. One-way ANOVA with Tukey's multiple comparisons test. n=10,10 (right); data pooled from 2 independent experiments. Two-tailed Student's t-test. **H**, n=5,5,5; data from one experiment. One-way ANOVA with Tukey's multiple comparisons test. **I**, n=5,6 data from one experiment. Student's t-test. **K**, n=3,3,3; data from one experiment. One-way ANOVA with Tukey's multiple comparisons test. **P**, n=26,22; data pooled from 5 independent experiments using male mice. **Q**, n=11,10; data pooled from 2 independent experiments. Student's t-test. **R**, n=14,17,17; data pooled from 4 independent experiments. One-way ANOVA with Tukey's multiple comparisons test. **S**, n=3,4,3,4; data representative of 2 independent experiments. One-way ANOVA with Tukey's multiple comparisons test. ns = not significant, * = $p \leq 0.05$, ** = $p \leq 0.01$, *** = $p \leq 0.001$, **** = $p \leq 0.0001$.

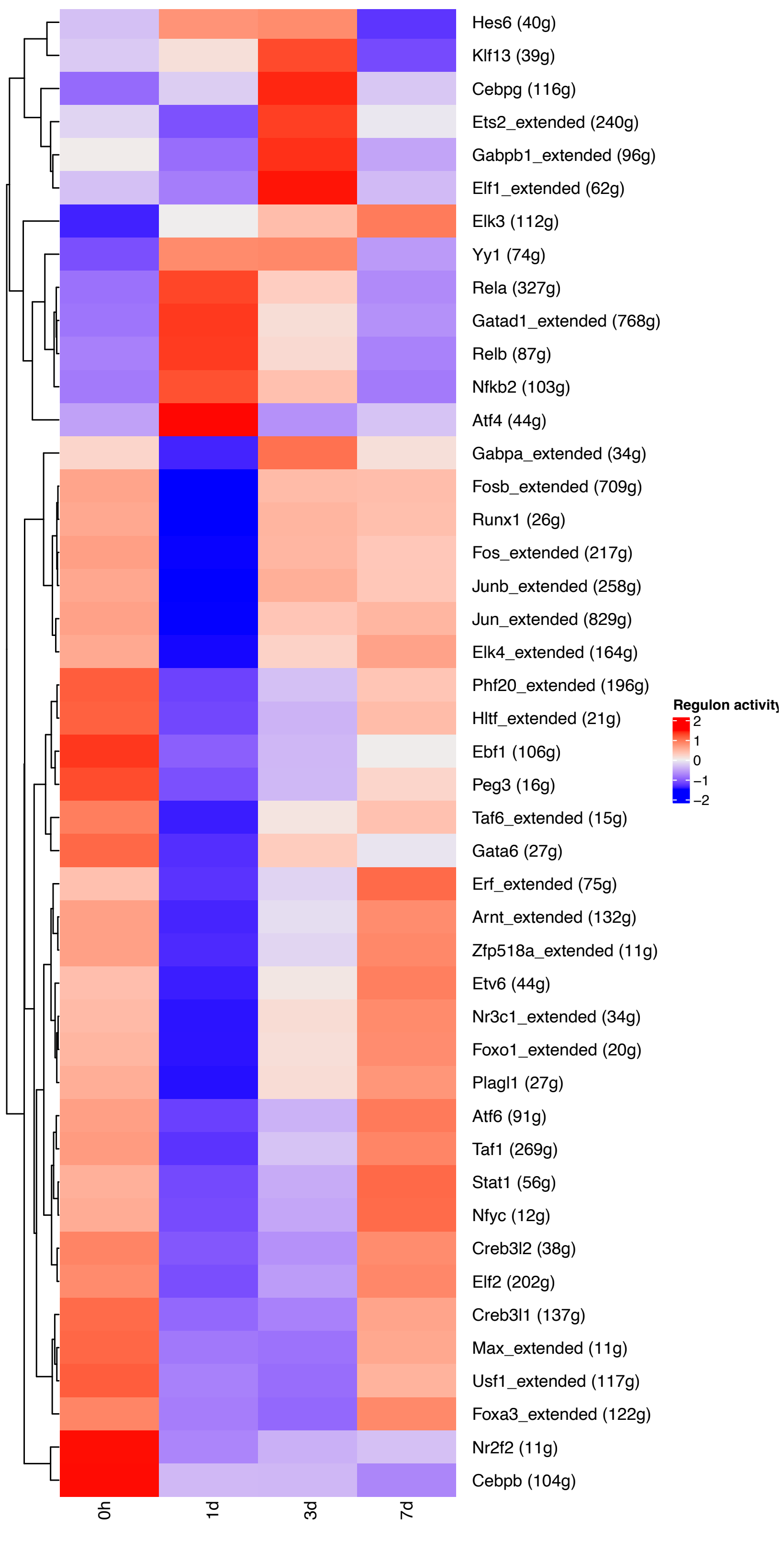


**Fig. S12. SCENIC analysis of acinar epithelial cells.**

The acinar cell clusters were analysed for regulon activity at the indicated times using SCENIC.

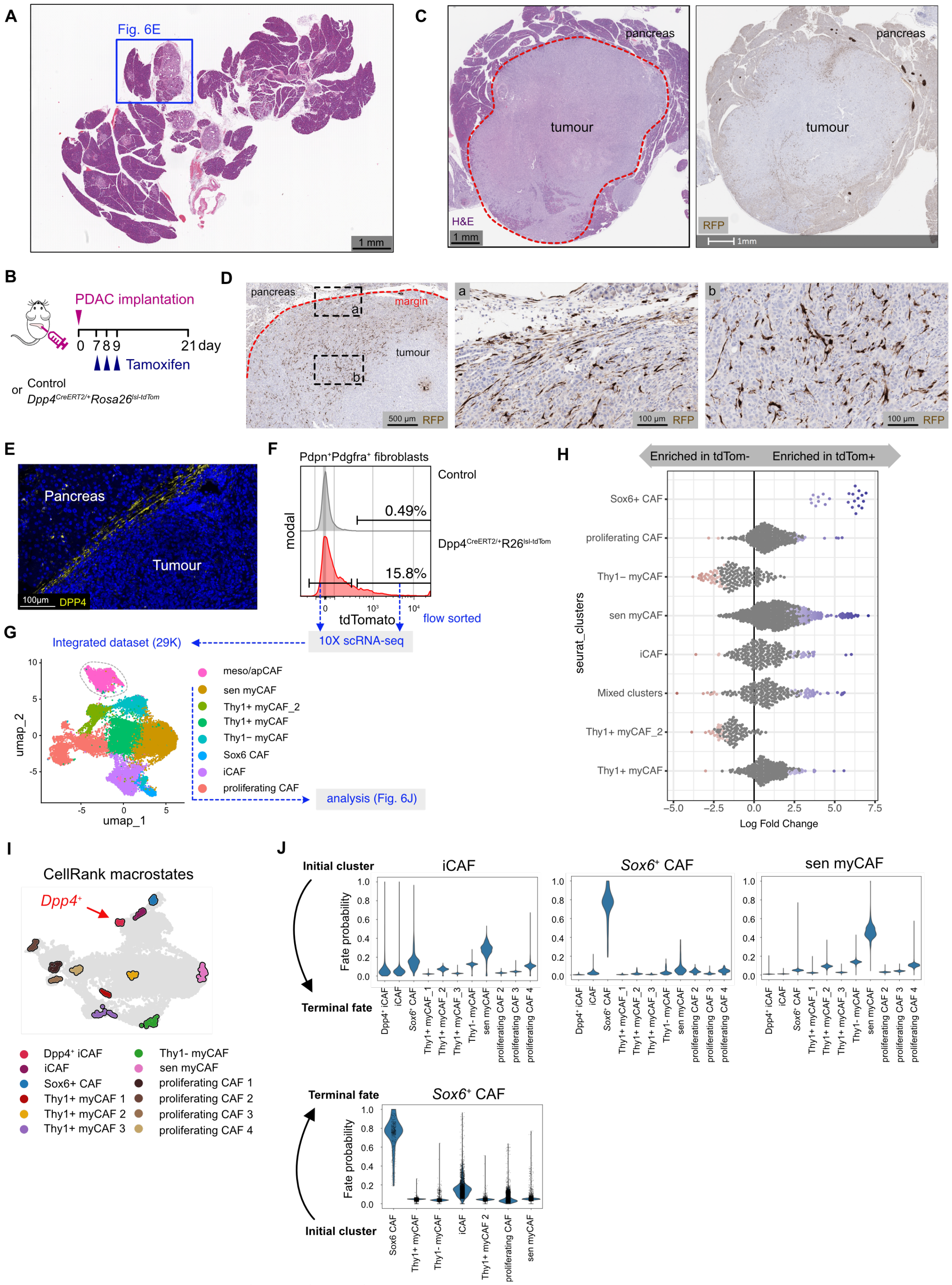

**Fig. S13. The ILC2-*Pi16*$^{+}$*Dpp4*$^{+}$*Ly6c*$^{+}$ fibroblast niche expands in pre-cancer and contributes to distinct CAF lineages in PDAC.**

**(A)** Three-month-old *Ptfa1*$^{Cre/+}$*Kras*$^{lsl\text{-}G12D}$ (KC) mouse FFPE pancreas was sectioned and stained for H&E.

(**B-E**) *Dpp4*$^{CreERT2/+}$*Rosa26*$^{lsl\text{-}tdTom}$ mice were orthotopically implanted with KPCY-derived tumour cells (2384c5) (*47*) on day 0, treated with tamoxifen on days 7, 8, and 9, and sacrificed on day 21 (**B**). Tumour-bearing pancreas was dissected for fixation, sectioning and histological (H&E, left) or immunohistochemistry (anti-RFP, right) analysis (**C**). Anti-RFP IHC staining at the tumour margin (*a*) and tumour core (*b*) (**D**). Immunofluorescence imaging of Dpp4 protein (yellow) and nuclei (blue) at the tumour margin (**E**).
(**F-J**) Tumours from *Dpp4*$^{CreERT2/+}$*Rosa26*$^{lsl\text{-}tdTom}$ mice (*treated as in Fig. S13B*) were harvested on day 21, followed by processing and staining for flow sorting. Live, $CD45^-EpCAM^-CD31^-Pdpn^+Pdgfra^+$ fibroblasts were assessed for tdTomato signal (using implanted WT mice as negative staining control), and both tdTomato-negative and tdTomato-positive fractions were purified and submitted for 10X scRNA-seq analysis (**F**). QC'ed data from both fractions was integrated, followed by unsupervised clustering and identification of cancer-associated fibroblast subsets (*26958 cells*). A distinct cluster of antigen-presenting (ap)CAF, which derive from mesothelial cells *(18)*, was excluded. All other CAFs were subsequently re-clustered and used for downstream analysis (**G**, *24428 cells*). Milo analysis was performed on fibroblast clusters to assess differential cellular neighbourhood abundance among Dpp4 lineage-traced (tdTom-positive) and non-lineage-traced (tdTomato-negative) cells (**H**). CellRank was used to infer cell-fate dynamics between the identified clusters or 'macrostates' (**I**), which predicted how specific clusters (iCAF, $Sox6^+$ CAF, and sen_myCAF, **J**, *top row*) contribute to the indicated terminal fate. Conversely, we could also infer the relative contribution of clusters towards a terminal $Sox6^+$ CAF fate (**J**, *bottom*).

Histogram show representative gating strategy; numbers indicate the percent of gated cells. Imaging data is representative of 5 mice (**A, C**), 5 mice (**D**), and 2 mice (**E**).

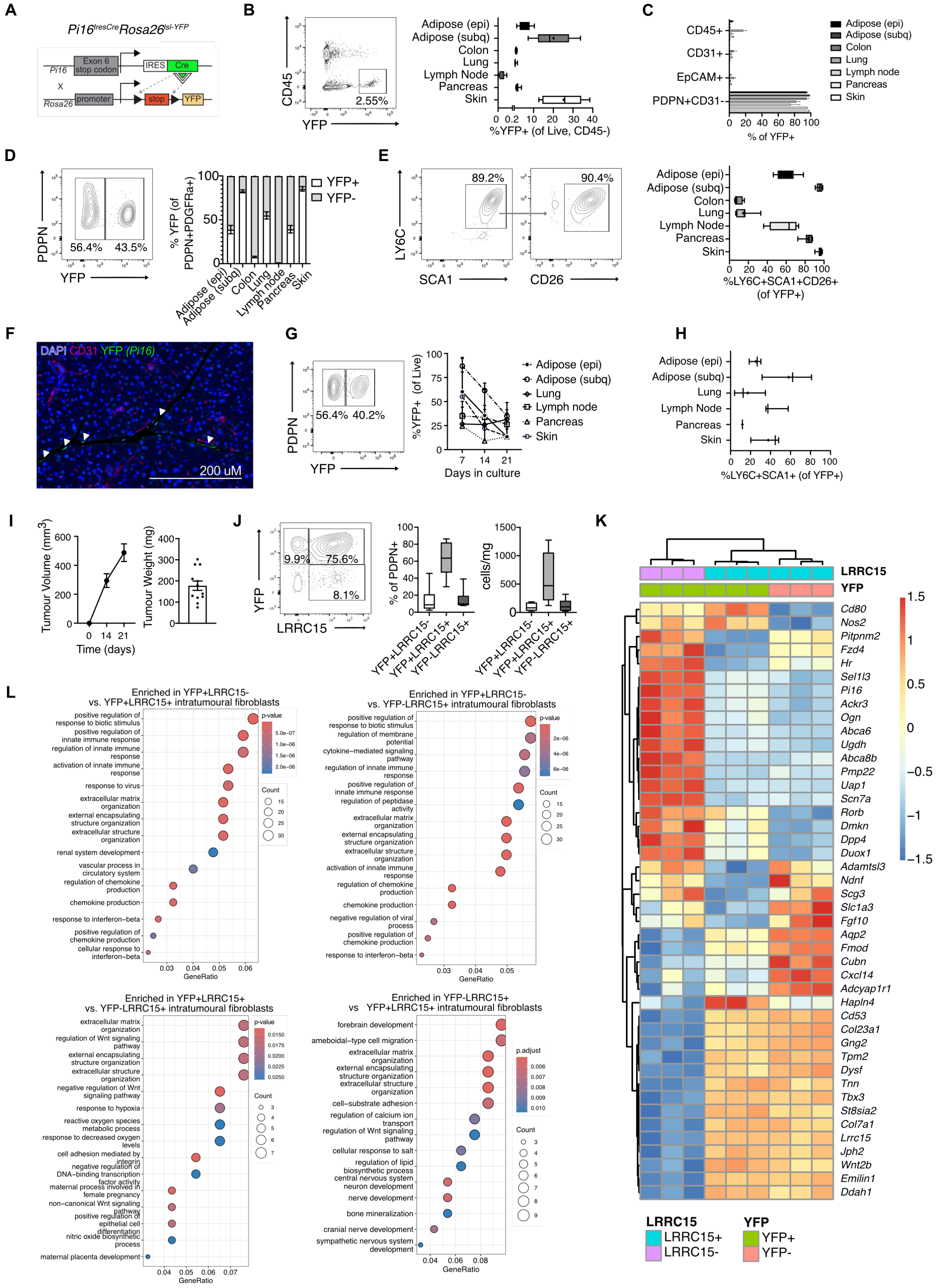

**Fig. S14.** ***Pi16***$^{+}$ **fibroblasts serve as progenitors for CAFs in a subcutaneous PDAC model.** (**A**) Schematic of *Pi16*$^{IresCre/+}$*Rosa26*$^{lsl\text{-}YFP}$ mouse model.

(**B**) Representative flow cytometry of *ex vivo* pancreatic $YFP^+$ cells, enumerated as percent of all live cells across tissues (Adipose - epididymal and subcutaneous (subq), Colon, Lung, Lymph Node, Pancreas, and Skin) shown right.

(**C**) Proportion of $YFP^+$ cells *ex vivo* that are $CD45^+$, $CD31^+$, $EpCAM^+$, or $Pdpn^+CD31^-$ across tissues.

(**D**) Representative flow of *ex vivo* pancreatic fibroblasts, enumerated proportion of fibroblasts (gated $CD45^-EpCAM^-CD31^-Pdpn^+Pdgfra^+$) that are $YFP^+$ across tissues shown right.

(**E**) Representative flow of pancreatic $YFP^+$ fibroblasts that are enriched for a $Ly6c^+Sca1^+CD26^+$ phenotype, enumerated across tissues shown right.

(**F**) Formalin fixed paraffin embedded (FFPE) $Pi16^{IresCre/+}Rosa26^{lsl\text{-}YFP}$ pancreata were sectioned, stained with primary antibodies against CD31 (Red) and GFP and subsequently stained with a A647-conjugated (Green) antibody specific to GFP primary antibody, then counterstained with DAPI (Blue). Scale bar: 200 μm. $GFP^+$ cells highlighted by arrows (**G**) Representative flow of *in vitro* pancreatic fibroblasts (gated $CD45^-EpCAM^-CD31^-PDPN^+$) at day 21 in culture, quantified across tissues at day 7, 14, and 21 shown right.

(**H**) Proportion of $YFP^+$ fibroblasts at day 21 that express Ly6C and Sca1 by flow cytometry.

(**I**) Volume (day 0, 14, and 21) and weight (day 21) of implanted subcutaneous KPC tumours.

(**J**) Representative *ex vivo* flow cytometry in KPC subcutaneous tumour implanted in $Pi16^{IresCre/+}Rosa26^{lsl\text{-}YFP}$ mouse of intratumoral fibroblasts (gated as $CD45^-CD24^-EpCAM^-CD31^-PDPN^+$) subgated by YFP and LRRC15, quantified as % of $PDPN^+$ (left) and cells per milligram of tissue (right).

(**K**) Expression of genes significantly enriched in FACS-sorted $YFP^+LRRC15^-$, $YFP^+LRRC15^+$, and $YFP^+LRRC15^-$ intratumoral fibroblast populations by bulk RNAseq.

(**L**) GSEA bubble plots showing top 15 enriched GO BP terms in intratumoral $YFP^+LRRC15^-$, $YFP^+LRRC15^+$, and $YFP^+LRRC15^-$ fibroblast populations.

Dot plots show representative gating strategy; numbers indicate the percent of gated cells. (**B-E**) n=4-9, pooled from 5 independent experiments; (**G-H**) n=3 for all tissues, except pancreas (n=1), pooled from 3 independent experiments; (**I**) n=9, across 3 independent experiments; (**J**) n=6, across 2 independent experiments. (**D**, **I**) Mean ± s.e.m. (**B**, **C**, **E**, **H**, **J**) centre lines in boxplots show median; box spans interquartile range, whiskers denote 10th– 90th percentile

| **Experiment** | **Genetic background** | **Effect on fibroblast numbers before AP** | **Effect on acinar cell damage after AP** | **Other notable effects** |
|---|---|---|---|---|
| **Constitutive** ILC2 deletion **(Fig. 6A, 6B)** | *Il7r*$^{Cre/+}$*Rora*$^{fl/fl}$ | Numbers reduced | Damage reduced | Reduced eosinophil influx |
| **Temporal** ILC2 deletion **(Fig. S11B)** | *Il5*$^{Cre/Cre}$*R26*$^{lsl\text{-}DTR}$ | No change | No change | Reduced acute fibroblast proliferation |
| Germline *Il13* deletion **(Fig. 6C)** | *Il13*$^{-/-}$ mice | Numbers reduced | Damage reduced | |
| OSM pre-treatment **(Fig. 6D)** | WT mice | Numbers reduced | Damage reduced | |
| Temporal fibroblast deletion **(Fig. S11P)** | *Fap*$^{DTR}$ mice | Numbers reduced | Damage reduced | Caveat: DT-induced deletion causes transient inflammation |
| Eosinophil deficient mice **(Fig. S11D, S11E)** | ΔdblGata mice | No change | No change | |
| Germline *Csf2* deletion **(Fig. S11I)** | *Csf2*$^{-/-}$ mice | No change | No change | |
| Germline *Il33* deletion **(Fig. S11R)** | *Il33*$^{-/-}$ mice | No change | No change | Reduced acute ILC2 activation & reduced eosinophil influx, and fibroblast proliferation |
| Other immune cell ablations or cytokine neutralization **(Fig. S11D, S11F-H)** | WT mice | No change | No change | NK, CD8, neutrophil, monocytes, TNFα, IL-1β do not affect acinar cell damage |

**Table S1: Summary of experiments showing effects on fibroblast density acute pancreatitis (AP) induced acinar cell damage.**

| **Primer ID** | **Sequence** | **Paired with** | **Rxn** |
|---|---|---|---|
| Pi16_5lr_F | ATCTCCACTATCTCTGATCCTTTGC | IRES_R1 | 5lr-QC |
| IRES_R1 | cttccttcacgacattcaacagac | Pi15_5lr_F1 | 5lr-QC |
| Pi16_3lr_R1 | TTCAATGGGTTAGGGTTCCTAGAAG | ERT2_F2 | 3lr-QC |
| ERT2_F2 | CAGGCACATGAGTAACAAAGGC | Pi16_3lr_R1 | 3lr-QC |
| Pi16_F9 | CCGACTGGGATTTGAAGAATTCTG | huACTB_R2 | sr1-QC |
| huACTB_R2 | catcctaggtgtggacatctcttg | Pi16_F9 | sr1-QC |
| Pgk1-polyA_F | gcagcctctgttccacatacacttca | Pi16_R7 | sr2-QC |
| Pi16_R7 | GAGGCTAAGATCCTCTGTCCTTAAG | huACTB_R1 | sr2-QC |

**Table S2: Primers used for quality control of *Pi16* targeted ES cells.**

| Protein | Manufacturer | Catalogue # | Antigen retrieval | Concentration |
|---|---|---|---|---|
| Podoplanin | BioLegend | 127402 | Citrate pH6 | 1:100 |
| IL-33 | abcam | ab187060 | Citrate pH6 | 1:250 |
| Dpp4 | abcam | ab187048 | Tris-EDTA pH9 | 1:500 |
| Endoglin | R&D Systems | AF1320-SP | Citrate pH6 | 1:100 |
| Fibronectin | abcam | ab2413 | Citrate pH6 | 1:100 |
| Gata3 | abcam | ab199428 | Tris-EDTA pH9 | 1:100 |
| CD3 | abcam | ab11089 | Citrate pH6 | 1:200 |
| BrdU | abcam | ab6326 | Citrate pH6 | 1:200 |
| F4/80 | Cell Signaling Technology | 70076S | Citrate pH6 | 1:200 |
| Lyve-1 | Thermo Scientific | 14-0443-82 | Citrate pH6 | 1:100 |
| Alpha smooth muscle actin | abcam | ab5694 | Citrate pH6 | 1:100 |
| RFP | abcam | ab124754 | Citrate pH6 | 1:500 |
| CD31 | Cell Signaling Technology | 77699S | Citrate pH6 | 1:250 |

**Table S3: List of antibodies and conditions used for multiplex immunofluorescence experiments.**

| **Primary antibody** | **Manufacturer** | **Catalogue #** | **Host species** | **Concentration** |
|---|---|---|---|---|
| Anti-Gata3 | Abcam | ab199428 | Rabbit | 1:200 |
| Anti-CD3 | Biolegend | 14-0033-85 | Syrian Hamster | 1:200 |
| Secondary antibody | Manufacturer | Catalogue # | Host species | Concentration |
| Anti-Rabbit AF555 | Abcam | ab150074 | Donkey | 1:500 |
| Anti-Syrian Hamster AF647 | Jackson ImmunoResearch | 107-605-142 | Goat | 1:500 |

**Table S4: List of antibodies and conditions used for cleared tissue imaging experiments.**

*See TIFF file: Movie S1*

**Movie S1:** 3D microscopy of optically clarified untreated C57Bl/6 pancreas, stained for CD3 (red) and Gata3 (blue). Imaging analysis identified the parenchyma (purple) and interstitial or capsular (white) niches, and Gata3$^{+}$CD3$^{-}$ ILC2 in the interstitium / capsular niche (cyan) or in the parenchyma (light pink). Representative of two independent experiments.

*See Excel file: Data S1*

**Data S1:** List of genes found to be differentially expressed between *Pi16*$^{+}$ and *Col15a1*$^{+}$ fibroblasts in untreated mice, as determined from per sample pseudobulk aggregate expression and subsequent DESeq2 differential expression testing, and filtering for genes with padj < 0.05.

*See Excel file: Data S2*

**Data S2:** List of genes associated with clusters of differentially expressed genes obtained from ILC2-fibroblast coculture experiments, as determined by hierarchical clustering (*related to Fig. S7H*).
